# The role of acetyl-coA carboxylase2 in head and neck squamous cell carcinoma

**DOI:** 10.7717/peerj.7037

**Published:** 2019-06-11

**Authors:** Kun Li, Chengcheng Zhang, Lei Chen, Pingping Wang, Yang Fang, Junwei Zhu, Shuo Chen, Juan Du, Bing Shen, Kaile Wu, Yehai Liu

**Affiliations:** 1 Department of Otorhinolaryngology, Head and Neck Surgery, The First Affiliated Hospital of Anhui Medical University, Hefei, Anhui, China; 2 Department of Urology, The First Affiliated Hospital of Anhui Medical University, Hefei, Anhui, China; 3 Department of Physiology, School of Basic Medical Sciences, Anhui Medical University, Hefei, Anhui, China

**Keywords:** Head and neck squamous cell carcinoma, Acetyl-CoA carboxylase2, Apoptosis, Survival time

## Abstract

**Background:**

Acetyl-CoA carboxylase (ACC) plays an important role in the metabolism of various cancer cells, but its role in head and neck squamous cell carcinoma (HNSCC) is uncertain. Therefore, in the present study, we explored the role of ACC2 in HNSCC.

**Methods:**

Western blot and immunohistochemistry assays were used to determine ACC2 protein expression levels in laryngocarcinoma and adjacent normal tissues derived from patients with laryngocarcinoma. ACC2 expression was knocked down in the hypopharyngeal cancer cell line FaDu to determine its effect on apoptosis. Lipid oil red staining was used to test the change of intracellular lipid.

**Results:**

The results showed that the ACC2 protein was highly expressed in laryngocarcinoma and that the ACC2 expression level was positively associated with the clinical cancer stage and negatively associated with the degree of laryngocarcinoma cell differentiation. Kaplan–Meier analyses indicated that compared with patients having low levels of ACC2, those with high ACC2 levels had a decreased 5-year survival rate. The results of western blot and terminal deoxynucleotidyl transferase dUTP nick-end labeling assays showed that knockdown of ACC2 accelerated apoptosis in FaDu cells. Furthermore, knockdown of ACC2 significantly reduced the intracellular lipid levels in FaDu cells.

**Conclusion:**

These findings suggest that ACC2 may be an important prognostic marker for patients with HNSCC and that ACC2 may be a potential target in the treatment of HNSCC.

## Introduction

Approximately 62,000 new head and neck cancer diagnoses and more than 13,000 deaths associated with this cancer occur in the United States every year (Kabarriti et al., 2018). Although treatment of head and neck squamous cell carcinoma (HNSCC) has greatly advanced, including through surgery, chemotherapy, immunotherapy, and radiation therapies, and early detection has substantially improved, the overall 5-year survival rate remains only 40–60% owing to uncontrolled invasion and metastasis (Gregoire et al., 2010; Elzakra et al., 2017; Xiang et al., 2015). In addition, the initial symptoms accompanying head and neck tumors, such as enlargement of lymph nodes, are common to many ailments and do not necessarily evoke immediate suspicion of HNSCC among patients or their physicians, leading to missed opportunities for early diagnosis. Later occurring symptoms, including difficultly in opening the mouth, swallowing, or breathing as well as poor digestion, lead to deterioration of patient health, and adverse reactions to treatment may further contribute to the poor prognosis of patients with HNSCC (Kabarriti et al., 2018). According to previous reports, several biomarkers of HNSCC, including SDF-1, CXCR4, HMGA2, CDKN2A, and TRPP2 (De-Colle et al., 2017; Palumbo et al., 2018; Chen et al., 2018; Wu et al., 2016), may potentially be used as therapeutic targets to overcome HNSCC. However, the currently identified biomarkers of HNSCC are challenged by a lack of specificity, sensitivity, or timeliness. Although previous study has demonstrated that SDF-1/CXCR4 axis may be a potentially therapeutic target for HNSCC (De-Colle et al., 2017), other studies shown that high CXCR4 is related to poor overall survival in the univariate but not in multivariate analysis (Albert et al., 2012) and SDF-1 expression is not statistically significantly associated with overall survival in multivariated analysis (Rave-Fränk et al., 2016). Moreover, De-Colle et al. (2017) pointed that the prognostic role of SDF-1 and CXCR4 is conflicting. Beside HNSCC, abnormal CXCR4 expression has also been found in many tumors, including breast, ovarian, prostate, oesophageal, bladder, colo-rectal, pancreatic, stomach (Balkwill, 2012). Therefore, novel biomarkers and treatment strategies for HNSCC are urgently needed to improve both diagnosis and patient prognosis.

The liver kinase B1–AMP-activated protein kinase–acetyl-CoA carboxylase (LKB1-AMPK-ACC) pathway is a major signaling pathway engaged in cell energy and lipid metabolism (Tanaka et al., 2017; Shackelford & Shaw, 2009). AMPK is directly activated by upstream LKB1, and the two proteins form a complex leading to the phosphorylation and inactivation of ACC, which plays an important role in the synthesis of fatty acids and regulation of energy metabolism (Hou et al., 2008; Santamarina et al., 2015). The LKB1-AMPK-ACC signaling pathway is reported to be involved in the proliferation of breast and ovarian cancers (Mauro et al., 2018; Rattan et al., 2011). Previous studies have demonstrated that inhibition of ACC reduces proliferation and lipogenesis in human glioblastoma cells (Jones et al., 2017). In addition, ACC is highly expressed and involved in carcinogenesis in malignant melanoma and other malignant neoplasms (Li et al., 2016; Kapur et al., 2005; Saab et al., 2018). There are two isoforms of ACCs: ACC1 (ACCα) and ACC2 (ACCβ), ACC1 is enriched in lipogenic tissues, such as the liver, adipose, and lactating mammary gland, while ACC2 is highly expressed in oxidative tissues, such as the skeletal muscle and heart, regulating fatty acid β-oxidation (Zu et al., 2013). Even one study reported that the expression level of phosphorylated ACC is significantly increased in the tissues of patients with HNSCC (Su et al., 2014), however, the functional role of ACC in HNSCC is currently unclear.

In the literature, the study in ACC2 is very little and its function is not well demonstrated. In the present study, the expression levels of ACC2 in HNSCC and normal tissues were compared using immunohistochemistry and western blot assays. Associations of ACC2 expression level with clinical stage, degree of cancer cell differentiation, and survival of patients with HNSCC were also investigated to explore the role of ACC2 in the development of HNSCC. In addition, FaDu cells, a hypopharyngeal cancer cell line, were transfected with ACC2 siRNA to determine whether knockdown of ACC2 enhanced cell apoptosis. We further used oil red staining to test whether ACC2 knockdown affected intracellular lipids in FaDu cells. The ultimate goal of these experiments was to determine whether development of compounds targeting ACC2 as therapeutic targets for HNSCC would be warranted.

## Materials and Methods

### Patients and tissue specimen preparation

All tissue samples (carcinoma and para-carcinoma tissues) from patients diagnosed as having laryngocarcinoma were collected at the First Affiliated Hospital of Anhui Medical University from 2012 to 2013. No distant metastases were found in any patient before surgery, and these patients were followed-up for 5 years after surgery to determine the survival rate (Table 1). Specimens were collected after receiving written informed consent from the participating patient. This study was approved by the Ethics Committee of Anhui Medical University (Ethical Application Ref: 20150192). The procedures were performed in accordance with the Declaration of Helsinki and good clinical practice.

**Table 1:** Patient characteristics.

Variable	Total	Cancer cell differentiation			*P*
		High differentiation	Middle differentiation	Low differentiation	
Ages, years					0.3179
>60	29	9	7	13	
≤60	22	7	10	5	
Sex					0.2343
Male	50	16	18	16	
Female	1	0	0	1	
Smoking					0.6418
Ever	45	14	17	14	
Never	6	2	1	3	
Alcohol					0.6976
Ever	39	13	14	12	
Never	12	4	3	5	
TNM stage					<0.0001
I or II	24	13	10	1	
III	27	3	8	16	
Survival status					0.0007
Alive	31	15	10	6	
Deceased	20	1	8	11	
ACC tumor expression					<0.0001
Low (≤45)	32	16	11	5	
High (>45)	19	0	7	12	

**Note:**

The table is showing that characteristics of patients with laryngeal carcinoma (*n* = 51). *P* = 0.3179 for ages > 60 vs. ages ≤ 60. *P* = 0.2343 for male vs. female. *P* = 0.6418 for ever smoke vs. Never smoke. *P* = 0.6976 for ever alcohol vs. Never alcohol. *P* < 0.0001 for stages I and II vs. stage III. *P* = 0.0007 for alive vs. deceased. *P* < 0.0001 for low ACC expression vs. high ACC expression.

### Cell culture, transfection, and regents

The FaDu cells and NP69 cells were purchased from American Type Culture Collection. The FaDu cells or NP69 cells were cultured in minimum essential medium Eagle’s (Mod.) or 1,640 medium (Wisent, USA) supplemented with 10% fetal bovine serum (Gibco, USA) and antibiotics (100 KU/L penicillin and 100 mg/L streptomycin) in an incubator at 37 °C with 5% CO_2_. The FaDu and NP69 cells were transiently transfected with ACC2 siRNA (sense, CCUGCCUACUUUCUUCUAUTT; antisense, AUAGAAGAAAGUAGGCAGGTT) (GenePharma, Shanghai, China) using Lipofectamine 3000 (Invitrogen, Carlsbad, CA, USA) following the manufacturer’s instructions and were cultured for another 48 h before being used in experiments.

### Western blot assay

The protein levels in FaDu cells or NP69 cells or in specimens were assessed by western blot assay. Briefly, the cells from different treatment groups or specimens were treated with RIPA lysis buffer (strong; Sigma, Ronkonkoma, NY, USA) on ice. The samples were then centrifuged at 4 °C and 12,000×*g* for 20 min. The protein was extracted from the supernatant, and the remaining supernatant was mixed with loading buffer using a ratio of 4:1 at 100 °C for 10 min. An equal amount of protein (30 μg) was loaded onto a gel for sodium dodecyl sulfate polyacrylamide gel electrophoresis and then transferred to a polyvinylidene fluoride membrane (Millipore, Burlington, MA, USA). The membranes were blocked with 5% nonfat milk for 1 h at room temperature and then incubated in 5% nonfat milk containing primary antibodies at 4 °C overnight. The membranes were incubated with the secondary antibody for 1 h at room temperature. Visualization of the secondary antibody was accomplished using an ECL detection system (Shanghai Peiqing Technology Co., Ltd, Shanghai, China). The optical density (OD) of each protein band was measured using the free software Image J (National Institutes of Health, Bethesda, MD, USA) and was normalized to β-tubulin (Biosharp, Hefei, China), which had been located in the same lane. The following primary antibodies were used in this study: rabbit anti-ACC2, rabbit anti-Bax, rabbit anti-Bcl-2, and rabbit anti-Caspase 3 (all antibodies were from Cell Signaling Technology, Danvers, MA, USA).

### Immunohistochemistry

Human laryngeal carcinoma tissues from patients were obtained during clinical surgery. Specimens were fixed with 4% paraformaldehyde and embedded in paraffin. The specimens were cut into five μm-thick sections, deparaffinized, and dehydrated. Antigen retrieval was accomplished by heating the sections in citrate buffer in a microwave oven for 15 min. The sections were then incubated with hydrogen peroxide (3%) for 30 min to destroy endogenous peroxidase activity. Subsequently, the sections were incubated with primary antibody to ACC2 (#3661; Cell Signaling Technology, Danvers, MA, USA) overnight at 4 °C prior to incubation with an anti-rabbit secondary antibody (Biosharp, Hefei, China). After being treated first with horseradish peroxidase and then with 3,3′-diaminobenzidine, the sections were counterstained with hematoxylin, dehydrated, cleared, and mounted. For the negative control group, the primary antibody was omitted. Images of stained sections were captured using a light microscope and analyzed with Image Pro Plus 5.1 (Media Cybernetics, Rockville, MD, USA) software.

### TUNEL analysis

The terminal deoxynucleotidyl transferase dUTP nick-end labeling (TUNEL) assay was conducted using a TUNEL kit (Vazyme, Najing, China) following the manufacturer’s instructions. Images were captured using fluorescence microscopy and analyzed with Image J software.

### Cell proliferation assay

The proliferation of FaDu and NP69 cells was tested by CCK8 Cell Counting Kit (Santa Cruz Biotechnology, Dallas, TX, USA). The cells were seeded on 96-well plates and transfected with ACC2 siRNA and scrambled siRNA, respectively. Incubated for 48 h in a 37 °C 5% CO_2_ incubator. Then, 10 μL of CCK8 solution was added to each well. After following incubation for 2 h, the absorbance of each well was measured at 450 nm. The data were expressed as OD.

### Lipids oil red stain

The intracellular lipids stain is following the manufacturer’s instructions of Oil Red O staining kit (Jiancheng Biotech, Nanjing, People’s Republic of China). All specimens were washed with 40% isopropyl alcohol and distilled water. Images were captured using an light microscope and analyzed with Image J software.

### Statistical analysis

SigmaPlot software was used to analyze all data. Data are expressed as means ± SEM. Two-tailed, unpaired Student’s *t*-tests were used to compare the results between groups. Values of *P* < 0.05 were considered statistically significant.

## Results

### Increased ACC2 expression in patients with laryngocarcinoma

**Figure 1:**
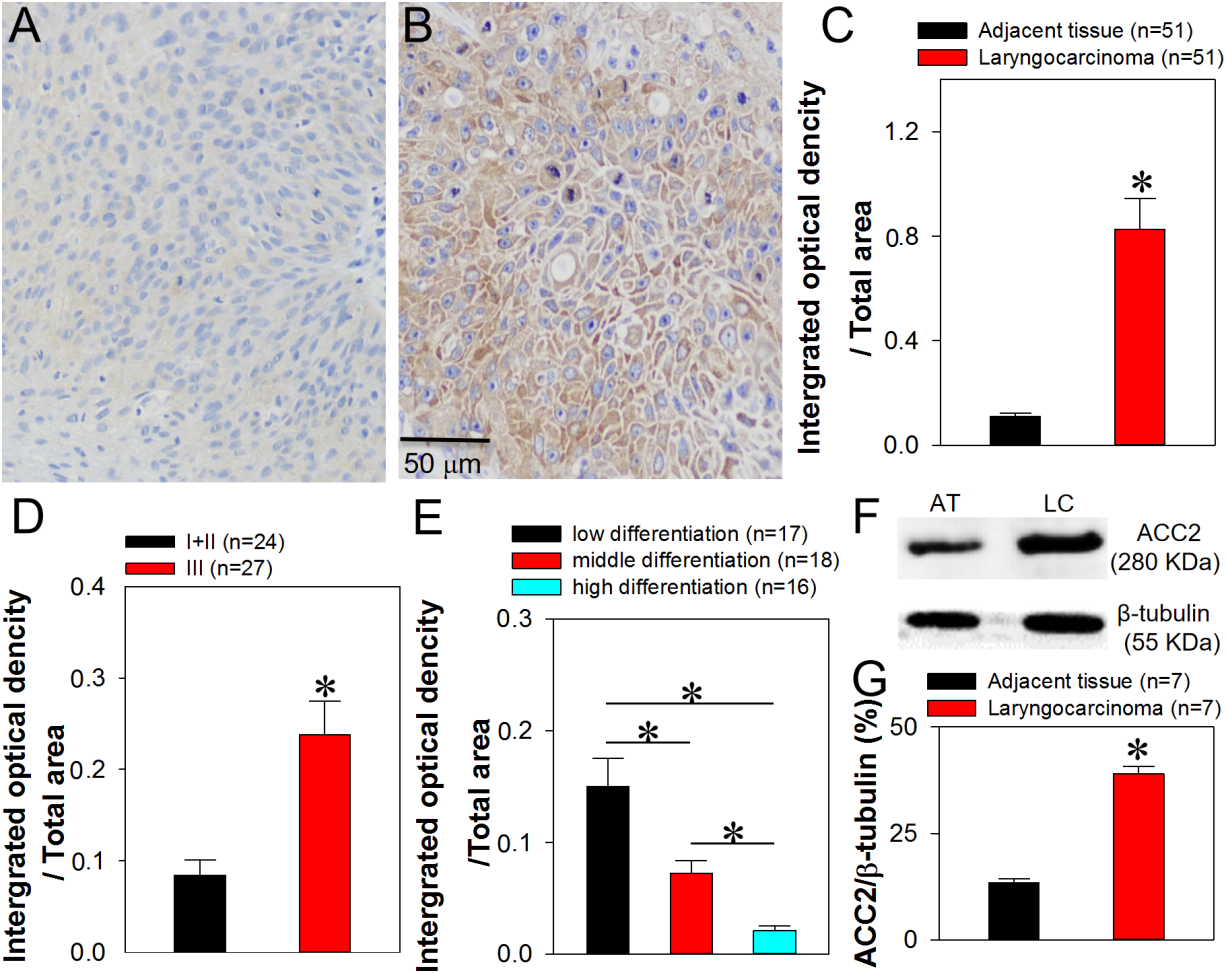
Increased ACC2 expression in head and neck squamous cell carcinoma tissue. (A–C) Representative immunohistochemical images (A and B) and summary data (C) showing ACC2 immunostaining in the normal tissue adjacent (A) to laryngocarcinoma tissue (B) (expressed as integrated optical density/total area). Values represent the mean ± SEM (*n* = 51). *P < 0.05. (D and E) Relationship between ACC2 expression level and clinical cancer stage (D) or degree of cell differentiation (E) in laryngocarcinoma and adjacent normal tissues. Values represent the mean ± SEM (*n* = 16–27). *P < 0.05. (F–G) Representative images (F) and western blot summary data (G) showing ACC2 expression in laryngocarcinoma tissue (LC) and the adjacent normal tissue (AT). Values represent the mean ± SEM (*n* = 7). *P < 0.05.

We demonstrated that the p-ACC expression level was significantly higher in laryngocarcinoma tissue than that in the adjacent tissue (Fig. S1), which was consistent with Su et al. (2014) Findings. Importantly, we used immunohistochemical analysis to evaluate ACC2 expression levels in laryngocarcinoma tissues obtained during surgery as clinical specimens from patients with laryngocarcinoma (Table 1). The ACC2 expression pattern differed between laryngocarcinoma and the adjacent normal tissues (Figs. 1A and 1B). In addition, the ACC2 expression level, represented as an integrated optical density (IOD) value, was significantly higher in laryngocarcinoma tissue than that in the adjacent tissue (Fig. 1C; Table 1). The results of western blot analyses of these tissues were consistent with this finding, that is, ACC2 protein expression levels in laryngocarcinoma tissue were significantly than higher than those in the adjacent normal tissue (Figs. 1F and 1G).

The laryngocarcinoma specimens used in immunohistochemical analysis were then analyzed to determine whether an association existed between the level of ACC2 expression and the clinical cancer stage or the degree of cell differentiation. Each patient’s clinical stage was classified as one of three stages (I, II, or III, with III being more advanced) based on the tumor-lymph node-metastasis (TNM) classification system. As shown in Fig. 1D, the ACC2 expression level for patients with stage III laryngocarcinoma was significantly higher (represented as a higher IOD value) than that for patients with stage I and II. Regarding a relationship between the degree of cell differentiation and the level of ACC2 expression, our results indicated that the ACC2 expression level was inversely associated with the degree of differentiation of the laryngocarcinoma cells, that is, the more poorly differentiated the cancer cells, the higher the ACC2 expression level (Fig. 1E). In summary, our findings indicated that ACC2 protein was highly expressed in laryngocarcinoma tissue and that the ACC2 expression level was significantly higher in patients with a more advance clinical stage and was significantly lower in patients having well-differentiated laryngocarcinoma cells.

### ACC2 expression level association with patient survival

**Figure 2:**
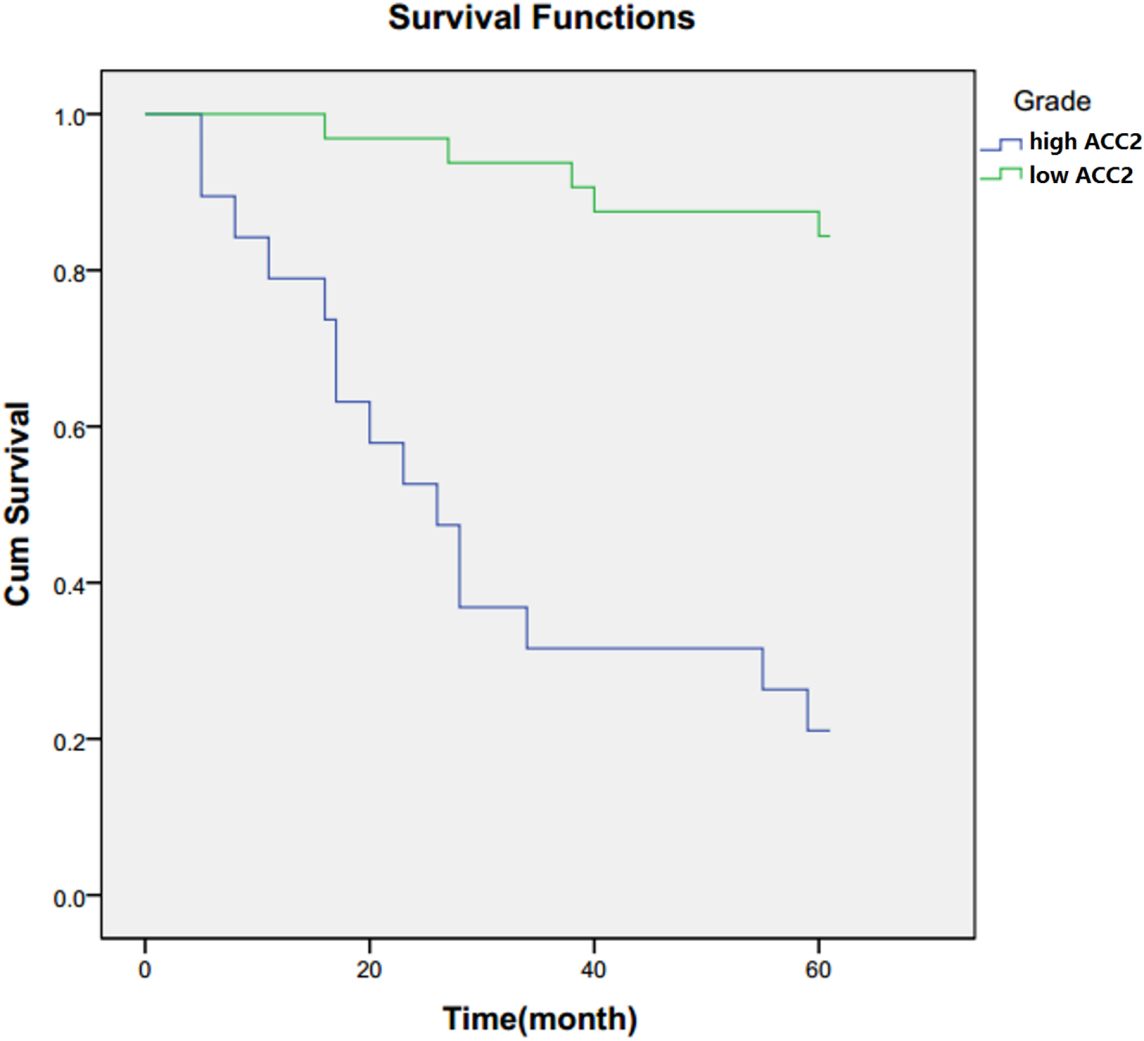
Association of patient survival with ACC2 expression level. Kaplan–Meier curves of 5-year survival among patients with laryngocarcinoma based on the expression level of ACC2. The green line represents the group of patients with low ACC2 expression, and the blue line represents the group of patients with high ACC2 expression.

Immunohistochemistry was performed to determine the ACC2 expression level in laryngocarcinoma tissue. The patients were divided into two groups, low ACC2 and high ACC2, based on the level of ACC2 expression. A total of 5-year patient survival data were obtained and the Kaplan–Meier method was used to investigate the association of survival time with ACC2 expression. As shown in Fig. 2, patients in the low ACC2 expression group survived longer than those in high ACC2 expression group. This result that ACC2 overexpression is associated with reduced 5-year survival in patients is consistent with our finding that ACC2 is higher in tissue from patients with a more advanced stage of laryngocarcinoma.

### Knockdown of ACC2 decreases intracellular lipids levels and enhances FaDu cell apoptosis

**Figure 3:**
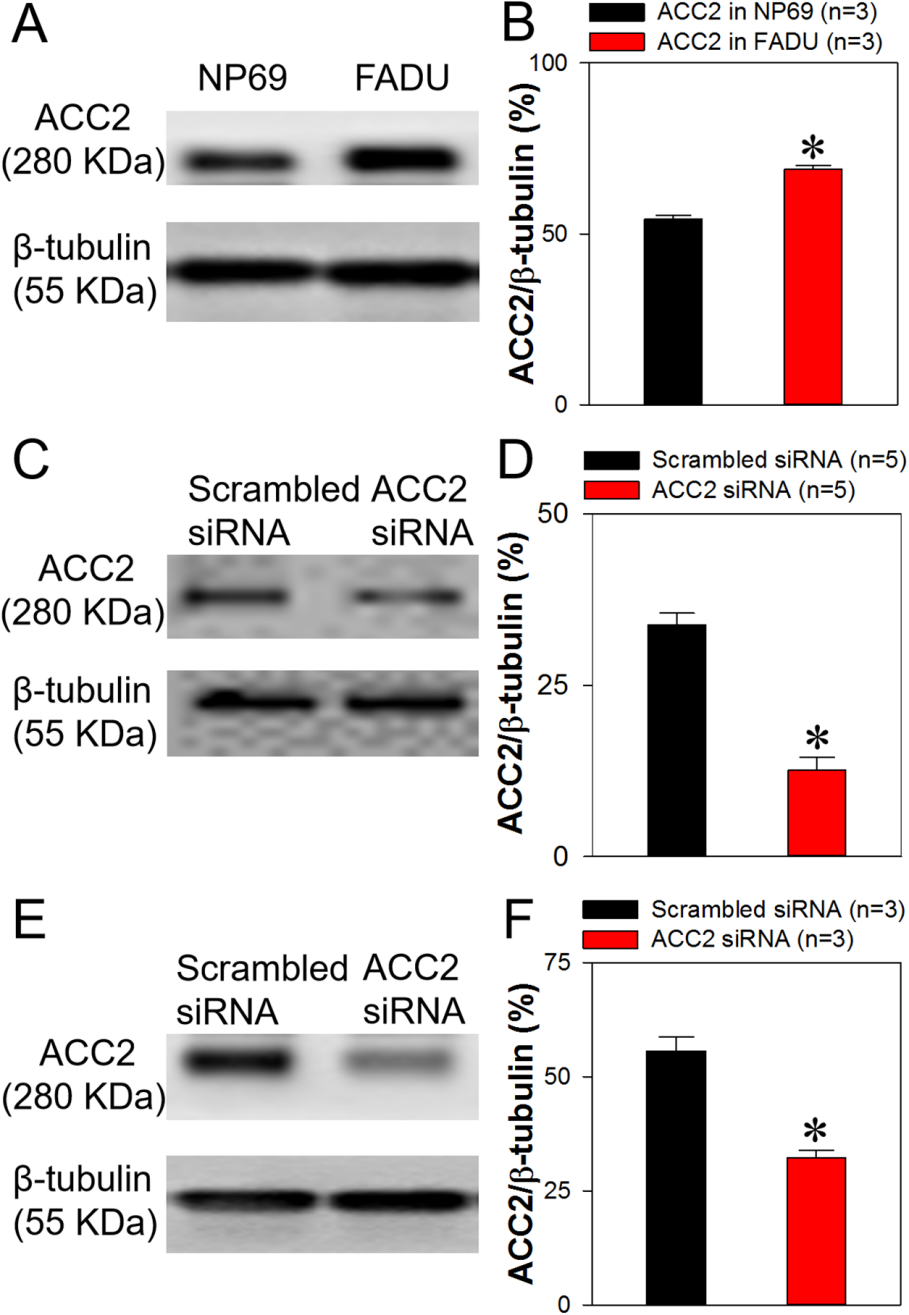
ACC2 expression levels and effect of ACC2 siRNA on expression of ACC2 in FaDu cells. (A and B) Representative images (A) and summary data (B) showing ACC2 expression in FaDu and NP69 cells. (C–F) Representative images (C and E) and summary data (D and F) showing ACC2 expression in FaDu (C and D) and NP69 (E and F) cells transfected with scrambled siRNA or ACC2 siRNA. Values represent the mean ± SEM (*n* = 3–5). *P < 0.05.

**Figure 4:**
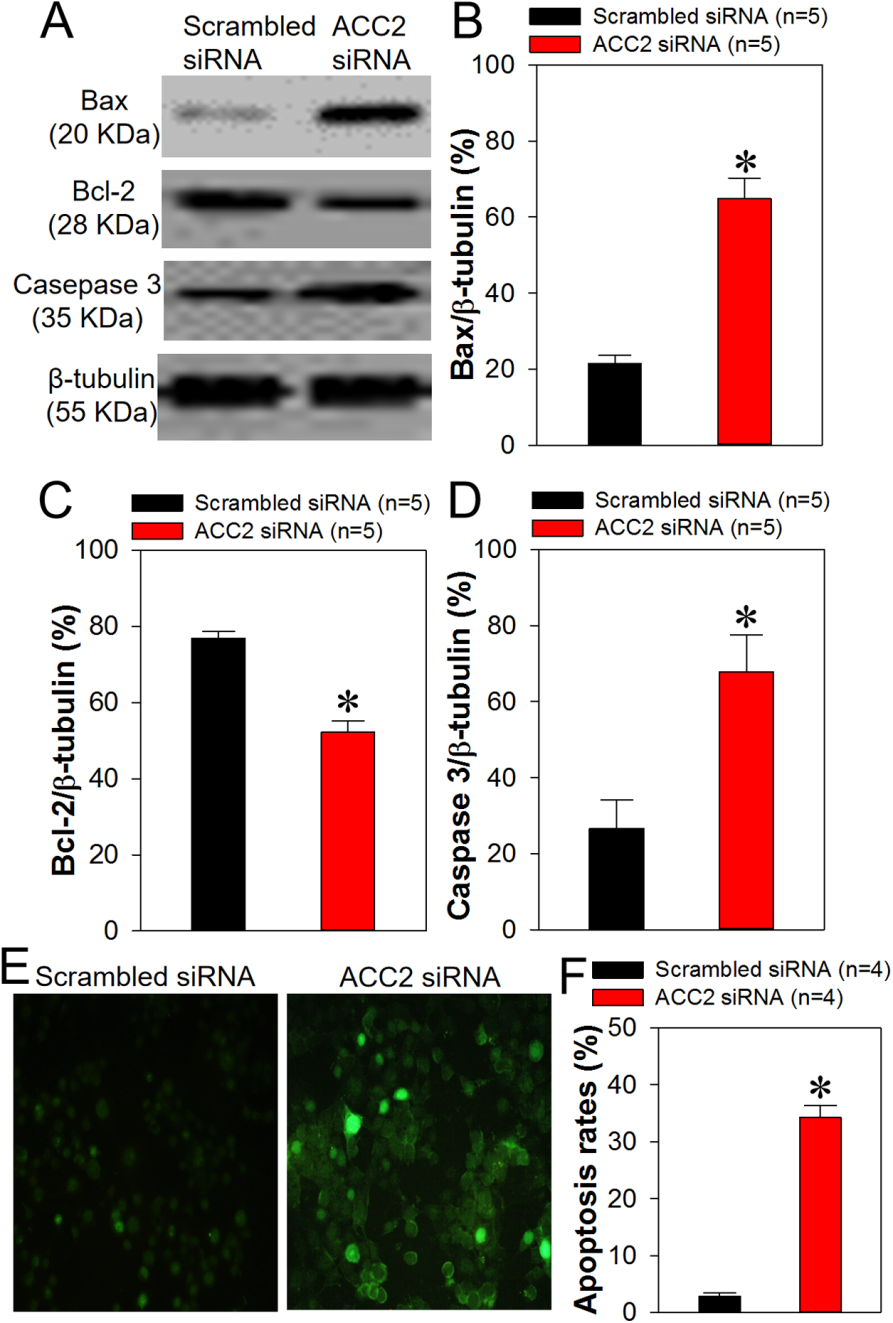
Role of ACC2 in apoptosis of FaDu cells. (A–D) Representative images (A) and summary data (B–D) showing bax, bcl-2, and caspase 3 expression levels in FaDu cells transfected with scrambled siRNA or ACC2 siRNA. (E and F) Representative images (E) and summary data (F) showing apoptotic FaDu cells transfected with scrambled siRNA or ACC2 siRNA. The change in apoptosis was evaluated using TUNEL staining. Images of TUNEL-positive cells were captured by a fluorescence microscope (magnification, 200×). Values represent the mean ± SEM (*n* = 4–5). *P < 0.05.

**Figure 5:**
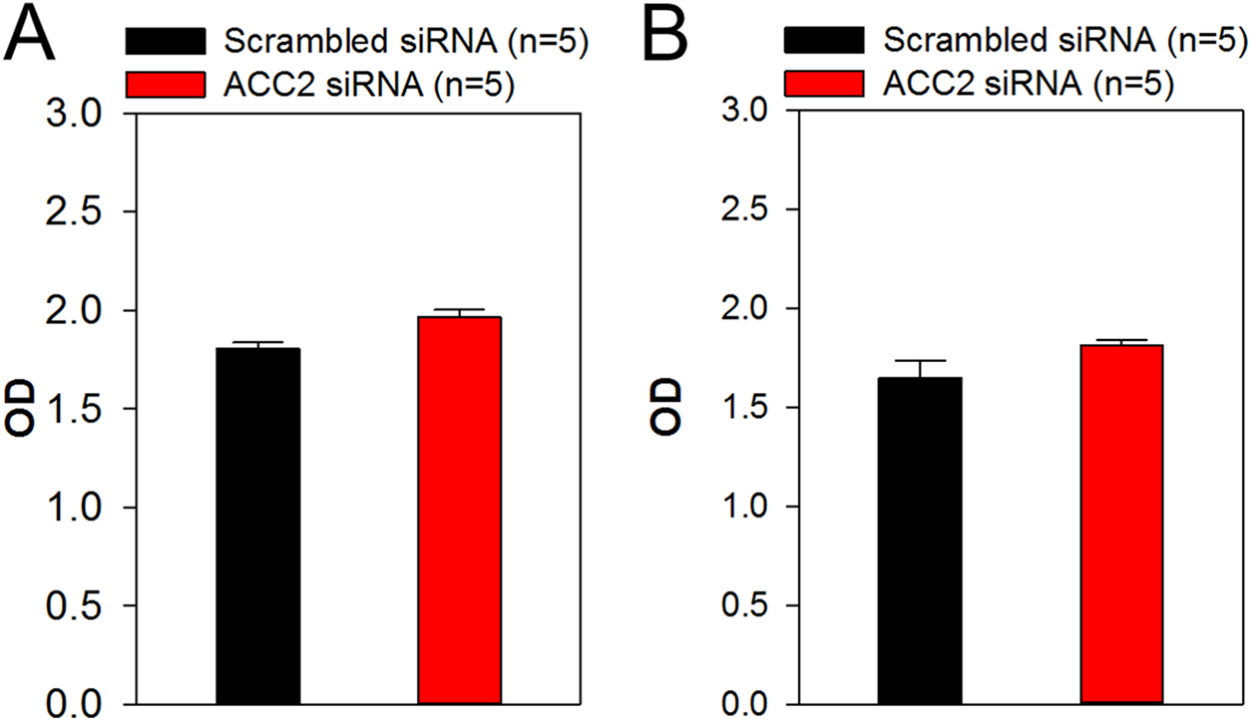
Effect of ACC2 siRNA on proliferation of FaDu and NP69 cell. (A and B) Summary data showing the proliferation of FaDu and NP69 cells transfected with scrambled siRNA or ACC2 siRNA for 24 h (expressed as optical density, OD). Values represent the mean ± SEM (*n* = 5). *P* > 0.05 for ACC2 siRNA vs. scrambled siRNA.

**Figure 6:**
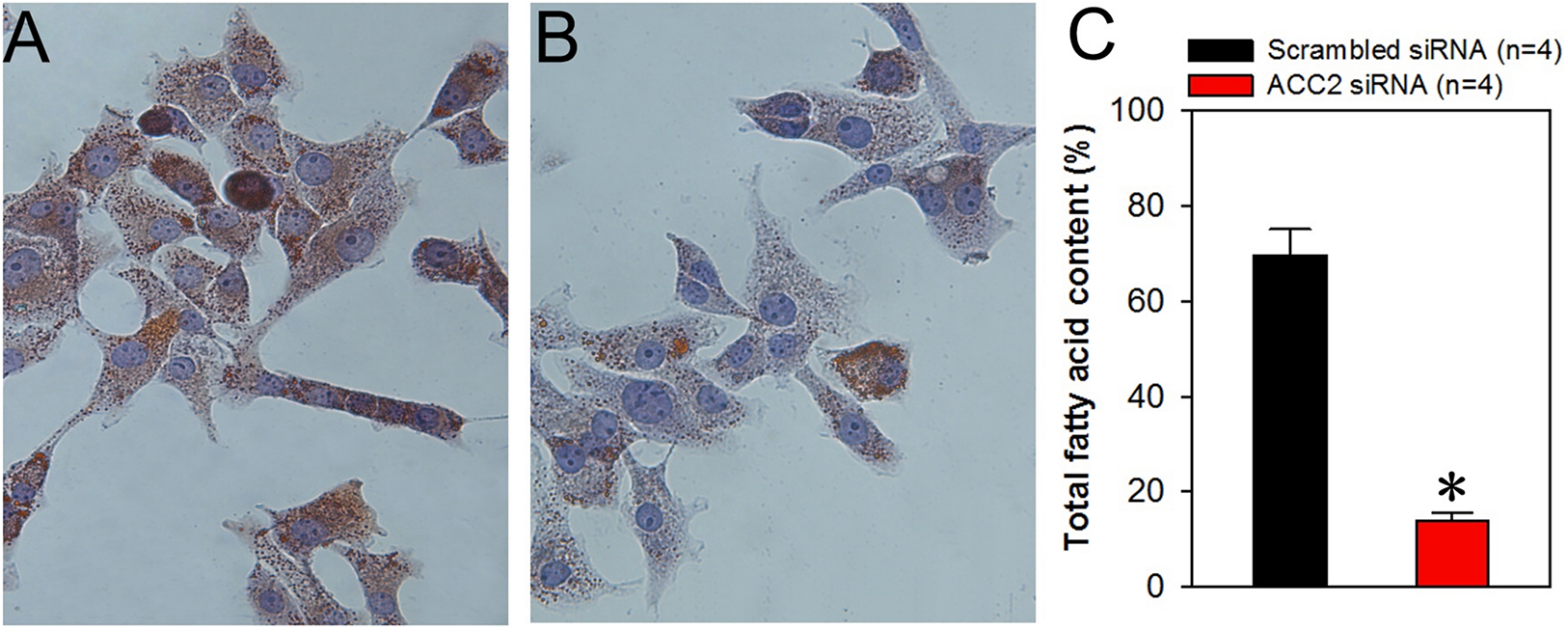
Role of ACC2 in lipids synthesis of FaDu cells. (A–C) Representative images (A and B) and summary data (C) showing the level of lipids in FaDu cells transfected with scrambled siRNA (A) and ACC2 siRNA (B). The change in intracellular lipids was evaluated using oil red stain. Images of intracellular lipids were captured by a light microscope (magnification, 400×). Values represent the mean ± SEM (*n* = 4). *P < 0.05.

Because we demonstrated in the present study that ACC2 is highly expressed in laryngocarcinoma and the prognosis for patients with laryngocarcinoma is poor owing to its rapid metastasis and growth, we next explored whether ACC2 affects apoptosis and proliferation in HNSCC. The FaDu cell line was derived from a hypopharyngeal carcinoma, a type of HNSCC. The NP69 cell line was derived from human nasopharyngeal epithelial cells, which was used as control cell line. Western blot analyses results showed that the expression level of ACC2 in FaDu cells was significantly higher than that in NP69 cells (Figs. 3A and 3B). Next, we used western blot analyses to detect protein expression levels for ACC2, bax, caspase 3, and bcl-2 in FaDu cells transfected with scrambled or ACC2 siRNAs. Our results indicated that the expression levels of ACC2 and bcl-2 proteins were markedly and significantly decreased, whereas those for bax and caspase 3 proteins were significantly increased, in FaDu cells transfected with ACC2 siRNA compared with those transfected with scrambled siRNA (Figs. 3C, 3D and 4A–4D). To examine apoptosis, we performed a TUNEL staining experiment. The results showed that more green fluorescence (representing apoptotic cells) was observed in FaDu cells transfected with ACC2 siRNA than in FaDu cells transfected with scrambled siRNA (Fig. 4E). Knockdown of ACC2 markedly increased apoptosis rates in FaDu and NP69 cells (Fig. 4F; Fig. S2). Additionally, we tested the effect of knockdown of ACC2 on the proliferation in Fadu and NP69 cells via ACC2 siRNA. Our results showed that ACC2 siRNA did not significantly affect the proliferation of FaDu and NP69 cells (Figs. 3C–3F, 5A and 5B). To investigate the cause of FaDu cell apoptosis induced by ACC2 knockdown, intracellular lipids levels were detected. The results showed that the lipids levels of cells transfected with ACC2 siRNA were significantly reduced, compared to those of cells transfected with scrambled siRNA (Fig. 6). Therefore, we demonstrated that knockdown of ACC2 accelerated FaDu cell apoptosis, possibly by interfering with intracellular lipids synthesis.

## Discussion

In the present study, we explored the role of ACC2 in laryngocarcinoma based on patient cancer stage, degree of cancer cell differentiation, patient survival time, and cell apoptosis. Our major findings were as follows: (1) ACC2 was highly expressed in laryngocarcinoma vs. adjacent normal tissues; (2) tissue from patients with a more advance clinical laryngocarcinoma stage expressed more ACC2 than that from patients in earlier clinical stages. The ACC2 expression level was inversely associated with the degree of differentiation of laryngocarcinoma cells; (3) high ACC expression was associated with decreased 5-year survival after surgery among patients with laryngocarcinoma; (4) ACC2 was highly expressed in FaDu cells compared to NP69 cells. (5) Knockdown of ACC2 in FaDu cells enhanced cell apoptosis but not significantly affected cell proliferation. (6) Knockdown of ACC2 in FaDu cells significantly decreased intracellular lipids levels. Taken together, our results suggest that ACC2 may play an important role in HNSCC apoptosis and indicate that the prognosis of patients with laryngocarcinoma is associated with ACC2 expression level. Therefore, ACC2 may be a potential therapeutic target in the treatment of HNSCC.

Laryngocarcinoma is one of the most common malignant neoplasms of the head and neck (Mouawad et al., 2014). In recent years, the incidence of laryngocarcinoma has increased, and this increase may be related to air pollution (Bobdey, Jain & Balasubramanium, 2015; Josyula et al., 2015). Despite receiving comprehensive treatment for laryngocarcinoma, the 5-year survival rate for patients is low and the prognosis of patients with advanced stage laryngocarcinoma is still poor. Therefore, creating new treatments for laryngocarcinoma is important. The association of cancer cell differentiation with clinicopathological characteristics is shown in Table 1. Multivariable analysis indicated a significant association between cancer cell differentiation and TNM stage, survival status, and ACC2 expression, but indicated no significant association between high or middle or low differentiation and ages, gender, smoking, and drinking. Therefore, expression level of ACC2 is closely related to the prognosis of tumor.

Several studies have shown that the LKB1-AMPK-ACC signaling pathway plays a non-negligible role in the development of tumors, including breast, prostate, ovarian, and hepatocellular carcinomas (Yang et al., 2018; Galdieri et al., 2016; Jeong et al., 2017). Acetyltransferase has also been shown to have an important role in the occurrence of some tumors (Liu et al., 2018; Dou et al., 2018; Zhang et al., 2018). ACC is a substrate for acetyltransferases, key enzymes in fatty acid synthesis. Low ACC expression results in decreased intracellular lipid synthesis (Olsen et al., 2010). In addition, ACC plays a particular role in the development and progression of cancer through its involvement in the intracellular homeostasis of fatty acids (Beckers et al., 2007). Previous studies have indicated that ACC is highly expressed in various tumor tissues, including malignant melanoma and other malignant neoplasms (Li et al., 2016; Kapur et al., 2005; Saab et al., 2018). Therefore, inhibition of ACC may be a new target for the treatment of malignant tumors. In previous study from other group (Su et al., 2014), the result showed that overexpressed p-ACC is important for evaluating HNSCC prognosis. In this study, we also demonstrated that the expression level of p-ACC was significantly increased in HNSCC tissues. More importantly, we also provide new evidence that ACC2 expression level is markedly higher in laryngocarcinoma tissue than in the adjacent normal tissue and higher in a late vs. an early clinical stage. Moreover, we also showed that the ACC2 expression level is inversely associated with the degree of laryngocarcinoma cell differentiation, and negatively associated with the 5-year survival rate. Our data using FaDu cells also demonstrated that transfection with ACC2 siRNA significantly decreased intracellular lipid levels and enhanced cell apoptosis. Taken together, our findings indicate that ACC2 may be involved in the diagnosis, development, progression, and prognosis of HNSCC.

## Conclusions

We demonstrated that ACC2 expression levels were higher in laryngocarcinoma tissues than in normal tissues, higher in later than in earlier clinical stages of laryngocarcinoma, and inversely associated with the degree of laryngocarcinoma cell differentiation. Higher ACC2 expression was associated with reduced five-survival rates in patients with laryngocarcinoma. Knockdown of ACC2 in FaDu cells enhanced apoptosis and decreased intracellular lipids levels, which suggested that knockdown of ACC2 enhanced FaDu cell apoptosis, possibly by interfering with intracellular lipid synthesis. Thus, we propose that the development of ACC2-based targeted therapy in the treatment of HNSCC is warranted.

## Supplemental Information

10.7717/peerj.7037/supp-1 Supplemental Information 1 ACC expression in laryngocarcinoma by immunohistochemistry and Western blots. The data is showing that ACC expression in laryngocarcinoma by immunohistochemistry and westernblots. The 12-L-C-1 is represented by 12- laryngocarcinoma-cancer-1 while 12-L-N-1 is represented by 12-laryngocarcinoma-normal tissue-1.

10.7717/peerj.7037/supp-2 Supplemental Information 2 Uncropped blots (Figure 3, Figure 4) and TUNEL images. The data is showing full-length uncropped blots (Figure 3, Figure 4) and apoptosis in FADU by TUNEL assays.

10.7717/peerj.7037/supp-3 Supplemental Information 3 NP69 cell apoptosis results. Images showing apoptotic NP69 cells transfected with scrambled siRNA or ACC2 siRNA.

10.7717/peerj.7037/supp-4 Supplemental Information 4 p-ACC expression in HNSCC (immunohistochemical analysis). Immunohistochemical images showing p-ACC immunostaining in the normal tissue adjacent to laryngocarcinoma tissue.

10.7717/peerj.7037/supp-5 Supplemental Information 5 Oil red lipid stain results. Images showing the level of lipids in FaDu cells transfected with scrambled siRNA and ACC2 siRNA.

10.7717/peerj.7037/supp-6 Supplemental Information 6 p-ACC expression in HNSCC (WB analysis). Western blot images showing p-ACC expression in laryngocarcinoma tissue and the adjacent normal tissue.

10.7717/peerj.7037/supp-7 Supplemental Information 7 Numerical data. Numerical data including immunohistochemistry, westernblot, CCK8, apoptosis rate, and survival data.

10.7717/peerj.7037/supp-8 Supplemental Information 8 Numerical data of revising figures. Numerical data containing NP69 cell apoptosis results, Oil red lipid stain results, p-ACC expression in HNSCC (immunohistochemical analysis), and p-ACC expression in HNSCC (WB analysis).

10.7717/peerj.7037/supp-9 Supplemental Information 9 Increased p-ACC expression in head and neck squamous cell carcinoma tissue. (A-C) Representative immunohistochemical images (A and B) and summary data (C) showing p-ACC immunostaining in the normal tissue adjacent (A) to laryngocarcinoma tissue (B) (expressed as integrated optical density/total area). D–E. Representative images (D) and western blot summary data (E) showing p-ACC expression in laryngocarcinoma tissue (LC) and the adjacent normal tissue (AT). Values represent the mean ± SEM (n = 4-18). *P < 0.05.

10.7717/peerj.7037/supp-10 Supplemental Information 10 Role of ACC2 in apoptosis of NP69 cells. (A-C) Representative images (A and B) and summary data (C) showing apoptotic NP69 cells transfected with scrambled siRNA or ACC2 siRNA. The change in apoptosis was evaluated using TUNEL staining. Images of TUNEL-positive cells were captured by a fluorescence microscope. Values represent the mean ± SEM (n = 4). *P < 0.05..

10.7717/peerj.7037/supp-11 Supplemental Information 11 Uncropped blots (Figures 3 and Figures 4). The data is showing ACC expression levels, effect of ACC siRNA on expression of ACC in FaDu cells and role of ACC in apoptosis of FaDu cells.

## Additional Information and Declarations

### Data Availability

The following information was supplied regarding data availability: Raw data is available in the Supplemental Files.

## References

[ref-1] Albert S, Hourseau M, Halimi C, Serova M, Descatoire V, Barry B, Couvelard A, Riveiro ME, Tijeras-Raballand A, De Gramont A, Raymond E, Faivre S (2012). Prognostic value of the chemokine receptor CXCR4 and epithelial-tomesenchymal transition in patients with squamous cell carcinoma of the mobile tongue. Oral Oncology.

[ref-2] Balkwill FR (2012). The chemokine system and cancer. Journal of Pathology.

[ref-3] Beckers A, Organe S, Timmermans L, Scheys K, Peeters A, Brusselmans K, Verhoeven G, Swinnen JV (2007). Chemical inhibition of acetyl-CoA carboxylase induces growth arrest and cytotoxicity selectively in cancer cells. Cancer Research.

[ref-4] Bobdey S, Jain A, Balasubramanium G (2015). Epidemiological review of laryngeal cancer: an Indian perspective. Indian Journal of Medical and Paediatric Oncology.

[ref-5] Chen WS, Bindra RS, Mo A, Hayman T, Husain Z, Contessa JN, Gaffney SG, Townsend JP, Yu JB (2018). CDKN2A copy number loss is an independent prognostic factor in HPV-negative head and neck squamous cell carcinoma. Frontiers in Oncology.

[ref-6] De-Colle C, Monnich D, Welz S, Boeke S, Sipos B, Fend F, Mauz P-S, Tinhofer I, Budach V, Jawad JA, Stuschke M, Balermpas P, Rödel C, Grosu A-L, Abdollahi A, Debus J, Bayer C, Belka C, Pigorsch S, Combs SE, Lohaus F, Linge A, Krause M, Baumann M, Zips D, Menegakis A (2017). SDF-1/CXCR4 expression in head and neck cancer and outcome after postoperative radiochemotherapy. Clinical and Translational Radiation Oncology.

[ref-7] Dou C, Liu Z, Tu K, Zhang H, Chen C, Yaqoob U, Wang Y, Wen J, Van Deursen J, Sicard D, Tschumperlin D, Zou H, Huang WC, Urrutia R, Shah VH, Kang N (2018). P300 acetyltransferase mediates stiffness-induced activation of hepatic stellate cells into tumor-promoting myofibroblasts. Gastroenterology.

[ref-8] Elzakra N, Cui L, Liu T, Li H, Huang J, Hu S (2017). Mass spectrometric analysis of SOX11-binding proteins in head and neck cancer cells demonstrates the interaction of SOX11 and HSP90α. Journal of Proteome Research.

[ref-9] Galdieri L, Gatla H, Vancurova I, Vancura A (2016). Activation of AMP-activated protein kinase by metformin induces protein acetylation in prostate and ovarian cancer cells. Journal of Biological Chemistry.

[ref-10] Gregoire V, Lefebvre JL, Licitra L, Felip E, EHNS-ESMO-ESTRO Guidelines Working Group (2010). Squamous cell carcinoma of the head and neck: EHNS-ESMO-ESTRO clinical practice guidelines for diagnosis, treatment and follow-up. Annals of Oncology.

[ref-11] Hou X, Xu S, Maitland-Toolan KA, Sato K, Jiang B, Ido Y, Lan F, Walsh K, Wierzbicki M, Verbeuren TJ, Cohen RA, Zang M (2008). SIRT1 regulates hepatocyte lipid metabolism through activating AMP-activated protein kinase. Journal of Biological Chemistry.

[ref-12] Jeong A, Kim J-H, Lee H-J, Kim SH (2017). Reactive oxygen species dependent phosphorylation of the liver kinase B1/AMP activated protein kinase/acetyl-CoA carboxylase signaling is critically involved in apoptotic effect of lambertianic acid in hepatocellular carcinoma cells. Oncotarget.

[ref-13] Jones JE, Esler WP, Patel R, Lanba A, Vera NB, Pfefferkorn JA, Vernochet C (2017). Inhibition of acetyl-CoA carboxylase 1 (ACC1) and 2 (ACC2) reduces proliferation and de novo lipogenesis of EGFRvIII human glioblastoma cells. PLOS ONE.

[ref-14] Josyula S, Lin J, Xue X, Rothman N, Lan Q, Rohan TE, Hosgood HD (2015). Household air pollution and cancers other than lung: a meta-analysis. Environmental Health.

[ref-15] Kabarriti R, Bontempo A, Romano M, McGovern KP, Asaro A, Viswanathan S, Kalnicki S, Garg MK (2018). The impact of dietary regimen compliance on outcomes for HNSCC patients treated with radiation therapy. Supportive Care in Cancer.

[ref-16] Kapur P, Rakheja D, Roy LC, Hoang MP (2005). Fatty acid synthase expression in cutaneous melanocytic neoplasms. Modern Pathology.

[ref-17] Li W, Zhang C, Du H, Huang V, Sun B, Harris JP, Richardson Q, Shen X, Jin R, Li G, Kevil CG, Gu X, Shi R, Zhao Y (2016). Withaferin A suppresses the up-regulation of acetyl-coA carboxylase 1 and skin tumor formation in a skin carcinogenesis mouse model. Molecular Carcinogenesis.

[ref-18] Liu J, Zhu JL, Zhang YL, Bai Y (2018). Expression pattern of FAM135B and K (lysine) acetyltransferase 5 in esophageal squamous cell carcinoma in Uygur patients. Journal of Southern Medical University.

[ref-19] Mauro L, Naimo GD, Gelsomino L, Malivindi R, Bruno L, Pellegrino M, Tarallo R, Memoli D, Weisz A, Panno ML, Andò S (2018). Uncoupling effects of estrogen receptor α on LKB1/AMPK interaction upon adiponectin exposure in breast cancer. FASEB Journal.

[ref-20] Mouawad F, Gros A, Rysman B, Bal-Mahieu C, Bertheau C, Horn S, Sarrazin T, Lartigau E, Chevalier D, Bailly C, Lansiaux A, Meignan S (2014). The antitumor drug F14512 enhances cisplatin and ionizing radiation effects in head and neck squamous carcinoma cell lines. Oral Oncology.

[ref-21] Olsen AM, Eisenberg BL, Kuemmerle NB, Flanagan AJ, Morganelli PM, Lombardo PS, Swinnen JV, Kinlaw WB (2010). Fatty acid synthesis is a therapeutic target in human liposarcoma. International Journal of Oncology.

[ref-22] Palumbo A, De Martino M, Esposito F, Fraggetta F, Neto PN, Valverde Fernandes P, Santos IC, Dias FL, Nasciutti LE, Meireles Da Costa N, Fusco A, Ribeiro Pinto LF (2018). HMGA2, but not HMGA1, is overexpressed in human larynx carcinomas. Histopathology.

[ref-23] Rattan R, Giri S, Hartmann LC, Shridhar V (2011). Metformin attenuates ovarian cancer cell growth in an AMP-kinase dispensable manner. Journal of Cellular and Molecular Medicine.

[ref-24] Rave-Fränk M, Tehrany N, Kitz J, Leu M, Weber HE, Burfeind P, Schliephake H, Canis M, Beissbarth T, Reichardt HM, Wolff HA (2016). Prognostic value of CXCL12 and CXCR4 in inoperable head and neck squamous cell carcinoma. Strahlentherapie und Onkologie.

[ref-25] Saab J, Santos-Zabala ML, Loda M, Stack EC, Hollmann TJ (2018). Fatty acid synthase and acetyl-CoA carboxylase are expressed in nodal metastatic melanoma but not in benign intracapsular nodal nevi. American Journal of Dermatopathology.

[ref-26] Santamarina AB, Oliveira JL, Silva FP, Carnier J, Mennitti LV, Santana AA, De Souza GH, Ribeiro EB, Oller do Nascimento CM, Lira FS, Oyama LM (2015). Green tea extract rich in epigallocatechin-3-gallate prevents fatty liver by AMPK activation via LKB1 in mice fed a high-fat diet. PLOS ONE.

[ref-27] Shackelford DB, Shaw RJ (2009). The LKB1–AMPK pathway: metabolism and growth control in tumour suppression. Nature Reviews Cancer.

[ref-28] Su YW, Lin YH, Pai MH, Lo AC, Lee YC, Fang IC, Lin J, Hsieh RK, Chang YF, Chen CL (2014). Association between phosphorylated AMP-activated protein kinase and acetyl-CoA carboxylase expression and outcome in patients with squamous cell carcinoma of the head and neck. PLOS ONE.

[ref-29] Tanaka M, Kita T, Yamasaki S, Kawahara T, Ueno Y, Yamada M, Mukai Y, Sato S, Kurasaki M, Saito T (2017). Maternal resveratrol intake during lactation attenuates hepatic triglyceride and fatty acid synthesis in adult male rat offspring. Biochemistry and Biophysics Reports.

[ref-30] Wu K, Shen B, Jiang F, Xia L, Fan T, Qin M, Yang L, Guo J, Li Y, Zhu M, Du J, Liu Y (2016). TRPP2 enhances metastasis by regulating epithelial-mesenchymal transition in laryngeal squamous cell carcinoma. Cellular Physiology and Biochemistry.

[ref-31] Xiang C, Lv Y, Wei Y, Wei J, Miao S, Mao X, Gu X, Song K, Jia S (2015). Effect of EphA7 silencing on proliferation, invasion and apoptosis in human laryngeal cancer cell lines Hep-2 and AMC-HN-8. Cellular Physiology and Biochemistry.

[ref-32] Yang L, He Z, Yao J, Tan R, Zhu Y, Li Z, Guo Q, Wei L (2018). Regulation of AMPK-related glycolipid metabolism imbalances redox homeostasis and inhibits anchorage independent growth in human breast cancer cells. Redox Biology.

[ref-33] Zhang X, Carlisle SM, Doll MA, Martin RCG, States JC, Klinge CM, Hein DW (2018). High N-acetyltransferase 1 5α-androstane-3β, expression is associated with estrogen receptor expression in breast tumors, but is not under direct regulation by estradiol, 17β-diol, or dihydrotestosterone in breast cancer cells. Journal of Pharmacology and Experimental Therapeutics.

[ref-34] Zu X, Zhong J, Luo D, Tan J, Zhang Q, Wu Y, Liu J, Cao R, Wen G, Cao D (2013). Chemical genetics of acetyl-CoA carboxylases. Molecules.

