# Supplementary figures and images for: The role of acetyl-coA carboxylase2 in head and neck squamous cell carcinoma
Source: PeerJ. 2019 Jun 11;7:e7037. doi: 10.7717/peerj.7037 (PMC6568254; doi:10.7717/peerj.7037)

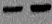

Supplement: Supplemental Information 1 — The data is showing that ACC expression in laryngocarcinoma by immunohistochemistry and westernblots. The 12-L-C-1 is represented by 12- laryngocarcinoma-cancer-1 while 12-L-N-1 is represented by 12-laryngocarcinoma-normal tissue-1. [file peerj-07-7037-s001.zip › Raw data 1/ACC expression in laryngocarcinoma/ACC(adjacent tissue-laryngocarcinoma)-2.png]

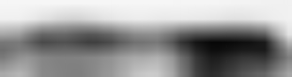

Supplement: Supplemental Information 1 — The data is showing that ACC expression in laryngocarcinoma by immunohistochemistry and westernblots. The 12-L-C-1 is represented by 12- laryngocarcinoma-cancer-1 while 12-L-N-1 is represented by 12-laryngocarcinoma-normal tissue-1. [file peerj-07-7037-s001.zip › Raw data 1/ACC expression in laryngocarcinoma/ACC(adjacent tissue-laryngocarcinoma)-3.png]

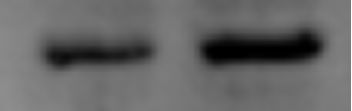

Supplement: Supplemental Information 1 — The data is showing that ACC expression in laryngocarcinoma by immunohistochemistry and westernblots. The 12-L-C-1 is represented by 12- laryngocarcinoma-cancer-1 while 12-L-N-1 is represented by 12-laryngocarcinoma-normal tissue-1. [file peerj-07-7037-s001.zip › Raw data 1/ACC expression in laryngocarcinoma/ACC(adjacent tissue-laryngocarcinoma)-1.png]

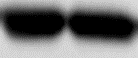

Supplement: Supplemental Information 1 — The data is showing that ACC expression in laryngocarcinoma by immunohistochemistry and westernblots. The 12-L-C-1 is represented by 12- laryngocarcinoma-cancer-1 while 12-L-N-1 is represented by 12-laryngocarcinoma-normal tissue-1. [file peerj-07-7037-s001.zip › Raw data 1/ACC expression in laryngocarcinoma/β-tubulin(adjacent tissue-laryngocarcinoma)-2.png]

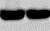

Supplement: Supplemental Information 1 — The data is showing that ACC expression in laryngocarcinoma by immunohistochemistry and westernblots. The 12-L-C-1 is represented by 12- laryngocarcinoma-cancer-1 while 12-L-N-1 is represented by 12-laryngocarcinoma-normal tissue-1. [file peerj-07-7037-s001.zip › Raw data 1/ACC expression in laryngocarcinoma/β-tubulin(adjacent tissue-laryngocarcinoma)-3.png]

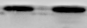

Supplement: Supplemental Information 1 — The data is showing that ACC expression in laryngocarcinoma by immunohistochemistry and westernblots. The 12-L-C-1 is represented by 12- laryngocarcinoma-cancer-1 while 12-L-N-1 is represented by 12-laryngocarcinoma-normal tissue-1. [file peerj-07-7037-s001.zip › Raw data 1/ACC expression in laryngocarcinoma/β-tubulin(adjacent tissue-laryngocarcinoma)1.tif]

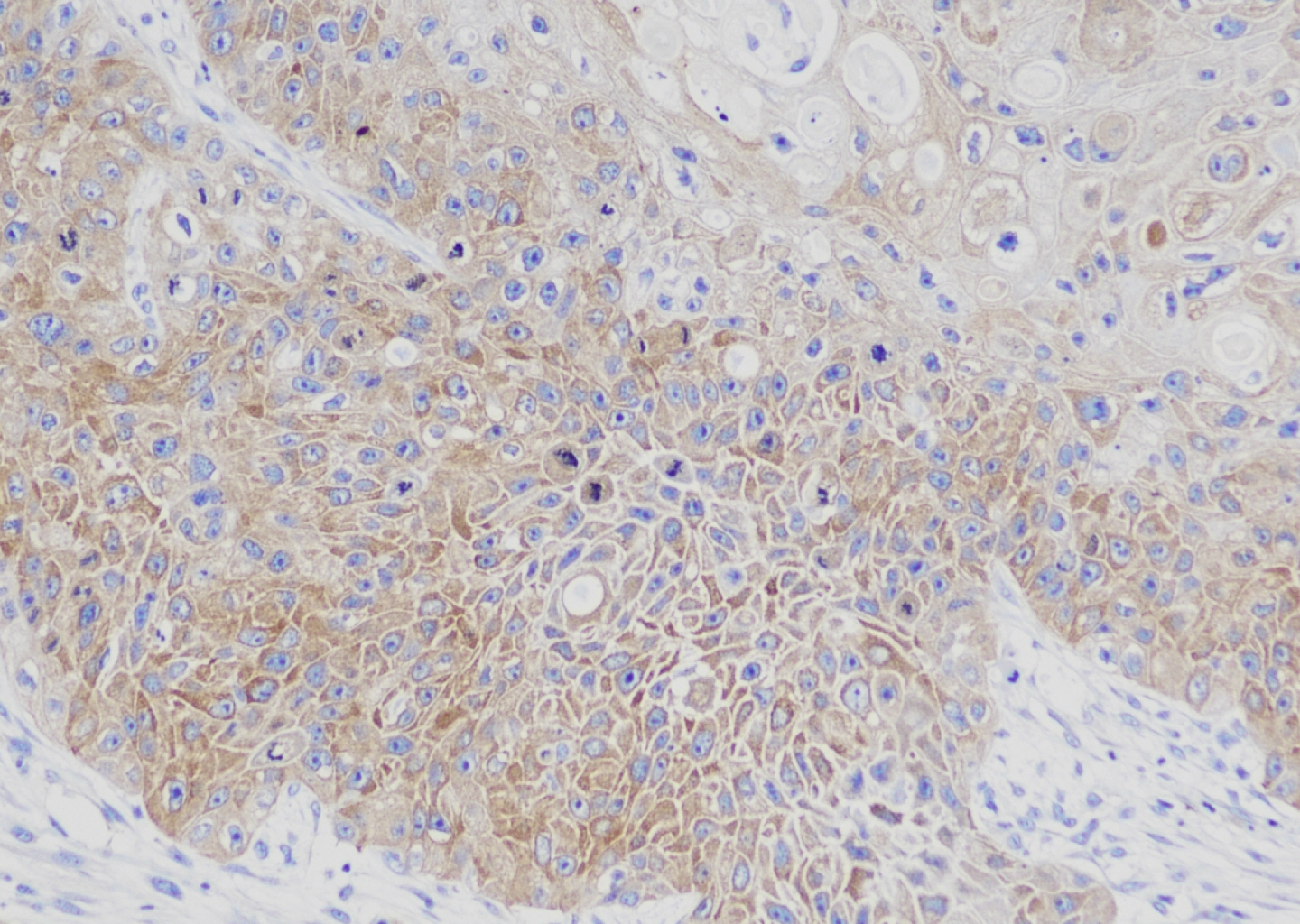

Supplement: Supplemental Information 1 — The data is showing that ACC expression in laryngocarcinoma by immunohistochemistry and westernblots. The 12-L-C-1 is represented by 12- laryngocarcinoma-cancer-1 while 12-L-N-1 is represented by 12-laryngocarcinoma-normal tissue-1. [file peerj-07-7037-s001.zip › Raw data 1/immunohistochemical images/12-L-C1 ACC.jpg]

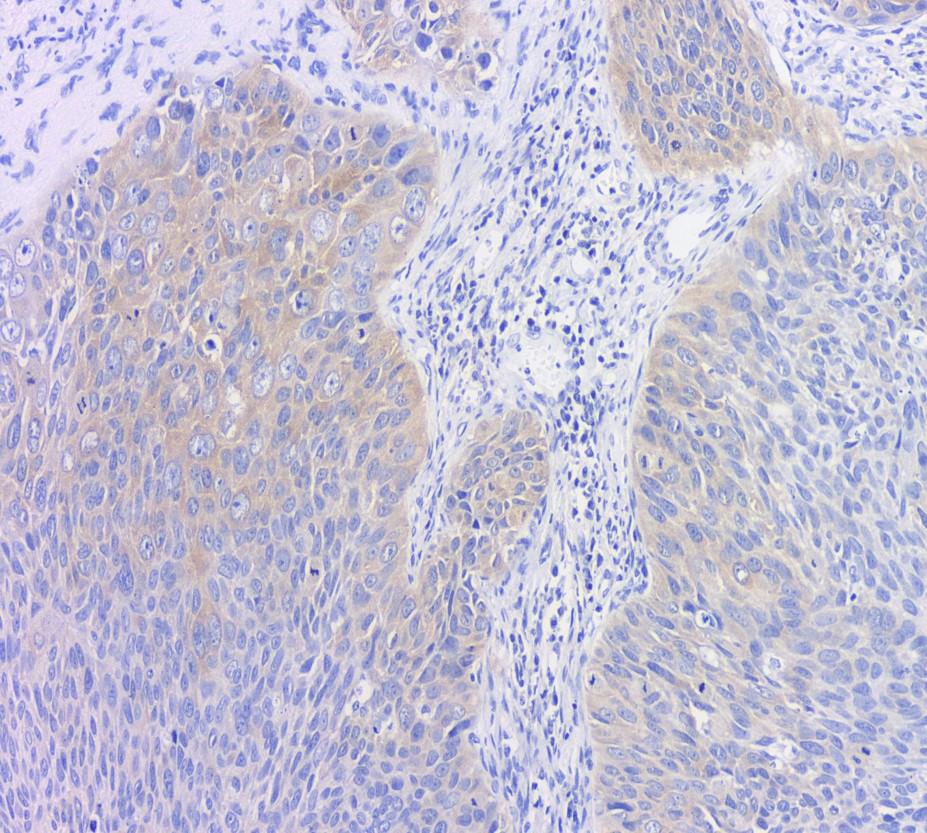

Supplement: Supplemental Information 1 — The data is showing that ACC expression in laryngocarcinoma by immunohistochemistry and westernblots. The 12-L-C-1 is represented by 12- laryngocarcinoma-cancer-1 while 12-L-N-1 is represented by 12-laryngocarcinoma-normal tissue-1. [file peerj-07-7037-s001.zip › Raw data 1/immunohistochemical images/12-L-C2 ACC.jpg]

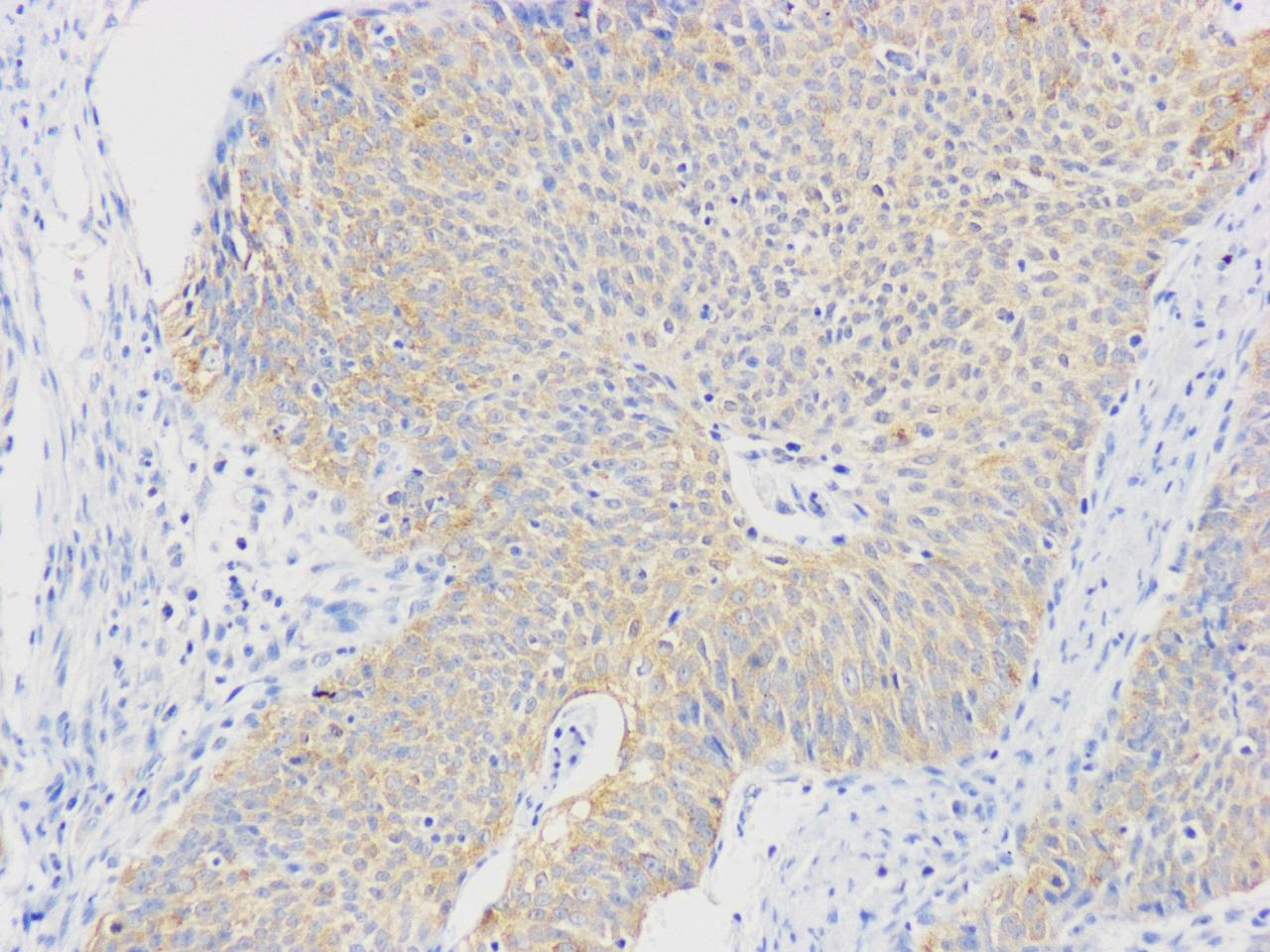

Supplement: Supplemental Information 1 — The data is showing that ACC expression in laryngocarcinoma by immunohistochemistry and westernblots. The 12-L-C-1 is represented by 12- laryngocarcinoma-cancer-1 while 12-L-N-1 is represented by 12-laryngocarcinoma-normal tissue-1. [file peerj-07-7037-s001.zip › Raw data 1/immunohistochemical images/12-L-C3 ACC.jpg]

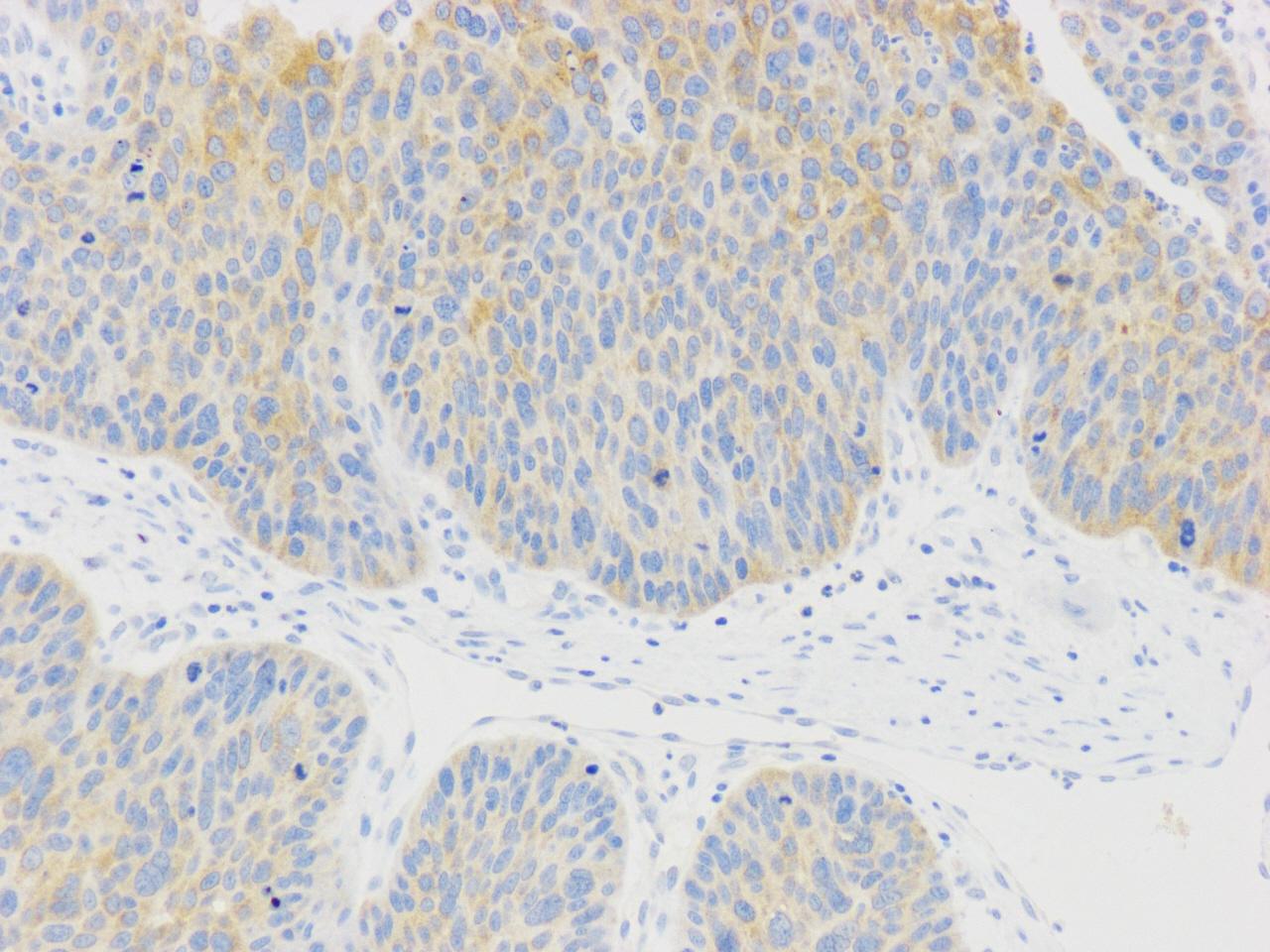

Supplement: Supplemental Information 1 — The data is showing that ACC expression in laryngocarcinoma by immunohistochemistry and westernblots. The 12-L-C-1 is represented by 12- laryngocarcinoma-cancer-1 while 12-L-N-1 is represented by 12-laryngocarcinoma-normal tissue-1. [file peerj-07-7037-s001.zip › Raw data 1/immunohistochemical images/12-L-C4 ACC.jpg]

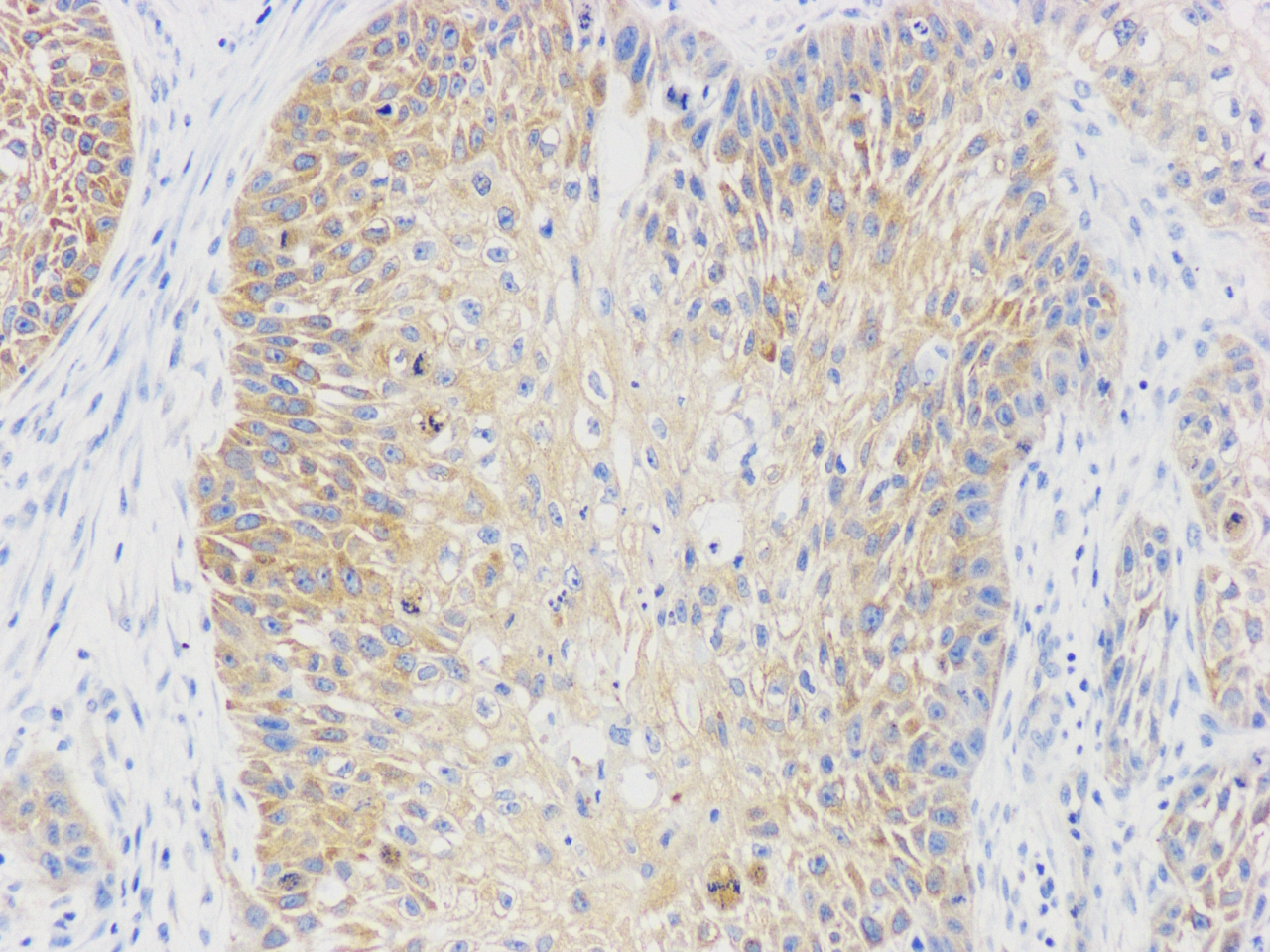

Supplement: Supplemental Information 1 — The data is showing that ACC expression in laryngocarcinoma by immunohistochemistry and westernblots. The 12-L-C-1 is represented by 12- laryngocarcinoma-cancer-1 while 12-L-N-1 is represented by 12-laryngocarcinoma-normal tissue-1. [file peerj-07-7037-s001.zip › Raw data 1/immunohistochemical images/12-L-C5 ACC.jpg]

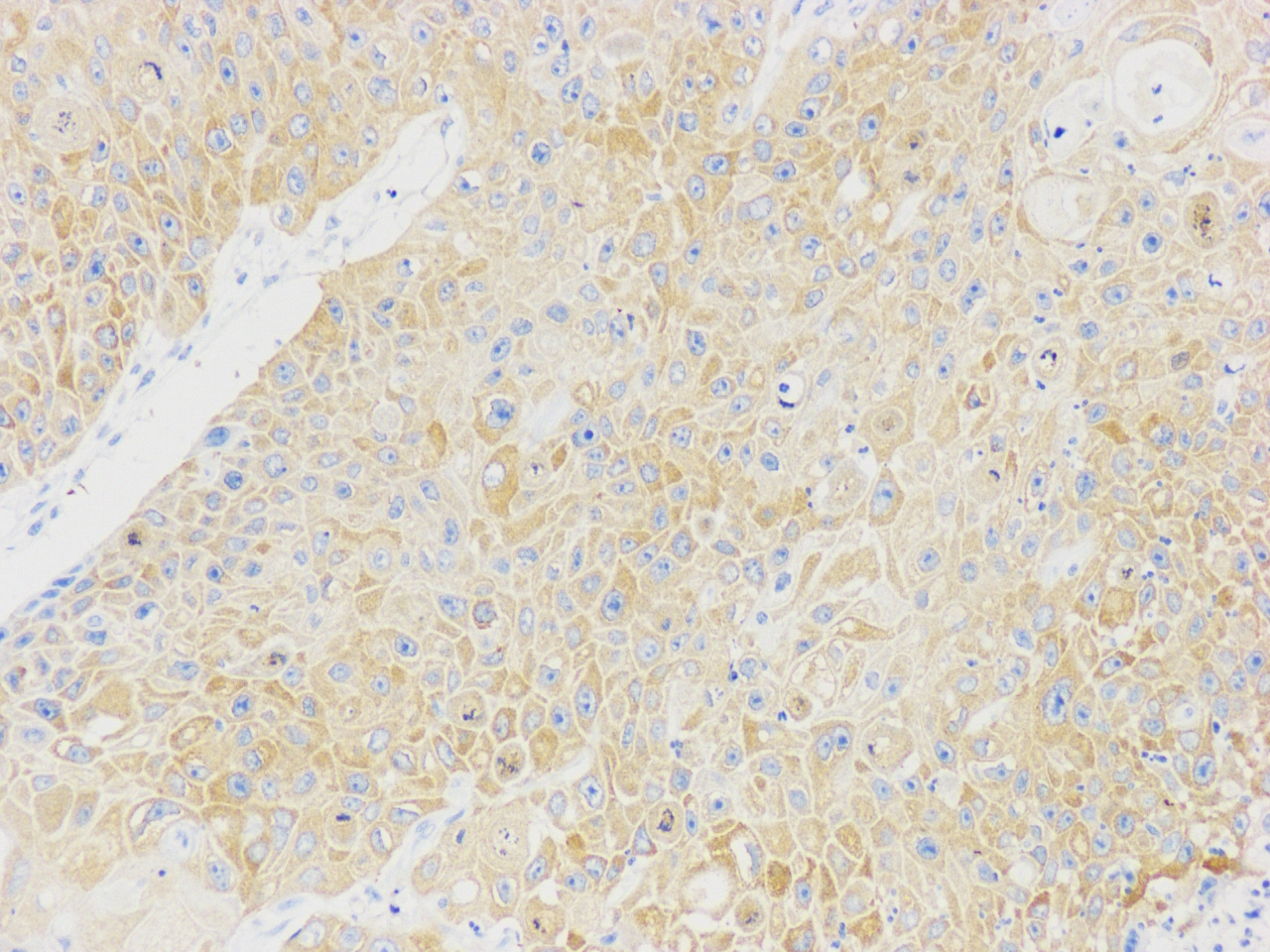

Supplement: Supplemental Information 1 — The data is showing that ACC expression in laryngocarcinoma by immunohistochemistry and westernblots. The 12-L-C-1 is represented by 12- laryngocarcinoma-cancer-1 while 12-L-N-1 is represented by 12-laryngocarcinoma-normal tissue-1. [file peerj-07-7037-s001.zip › Raw data 1/immunohistochemical images/12-L-C6 ACC.jpg]

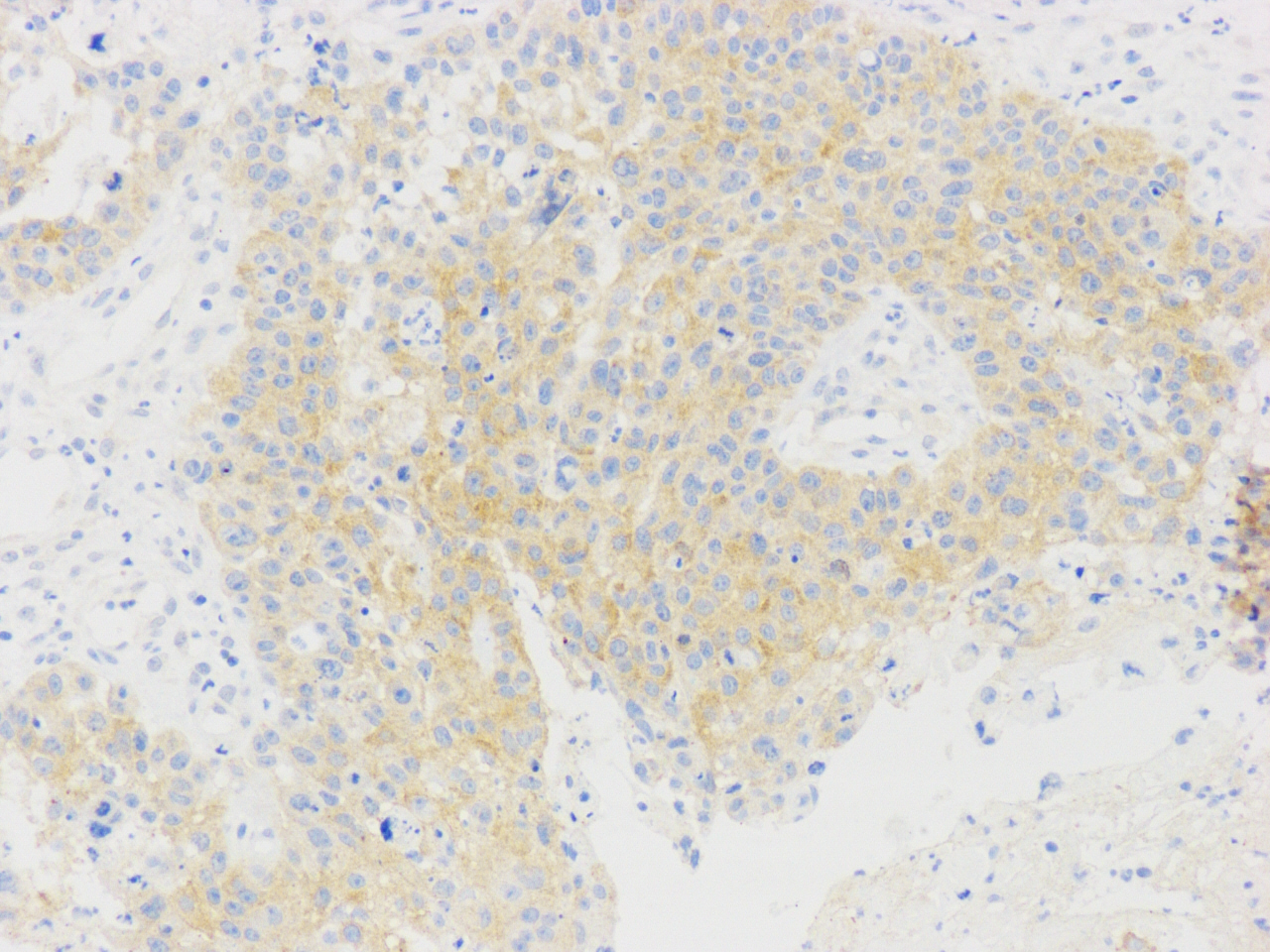

Supplement: Supplemental Information 1 — The data is showing that ACC expression in laryngocarcinoma by immunohistochemistry and westernblots. The 12-L-C-1 is represented by 12- laryngocarcinoma-cancer-1 while 12-L-N-1 is represented by 12-laryngocarcinoma-normal tissue-1. [file peerj-07-7037-s001.zip › Raw data 1/immunohistochemical images/12-L-C7 ACC.jpg]

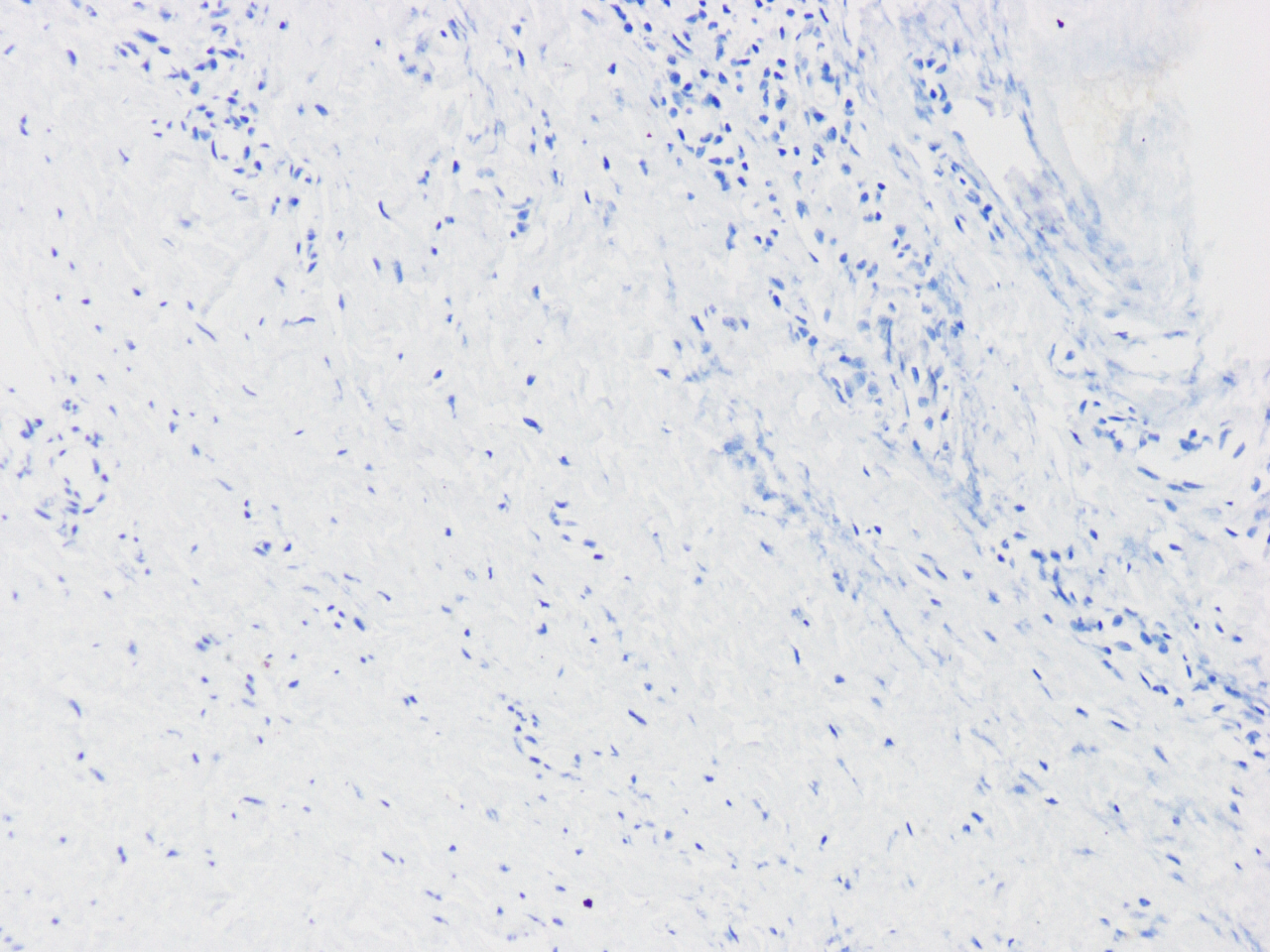

Supplement: Supplemental Information 1 — The data is showing that ACC expression in laryngocarcinoma by immunohistochemistry and westernblots. The 12-L-C-1 is represented by 12- laryngocarcinoma-cancer-1 while 12-L-N-1 is represented by 12-laryngocarcinoma-normal tissue-1. [file peerj-07-7037-s001.zip › Raw data 1/immunohistochemical images/12-L-N1 ACC.jpg]

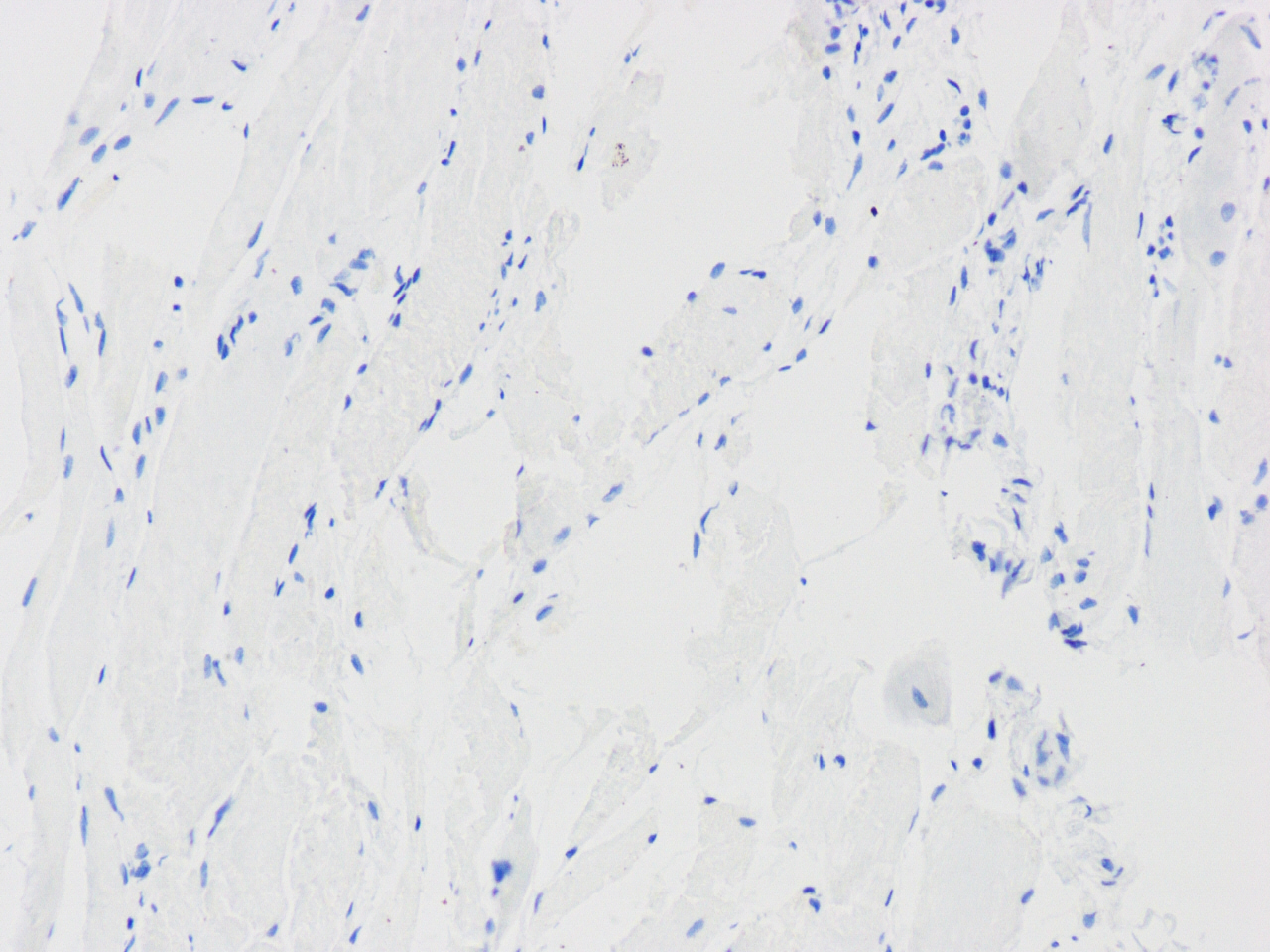

Supplement: Supplemental Information 1 — The data is showing that ACC expression in laryngocarcinoma by immunohistochemistry and westernblots. The 12-L-C-1 is represented by 12- laryngocarcinoma-cancer-1 while 12-L-N-1 is represented by 12-laryngocarcinoma-normal tissue-1. [file peerj-07-7037-s001.zip › Raw data 1/immunohistochemical images/12-L-N2 ACC.jpg]

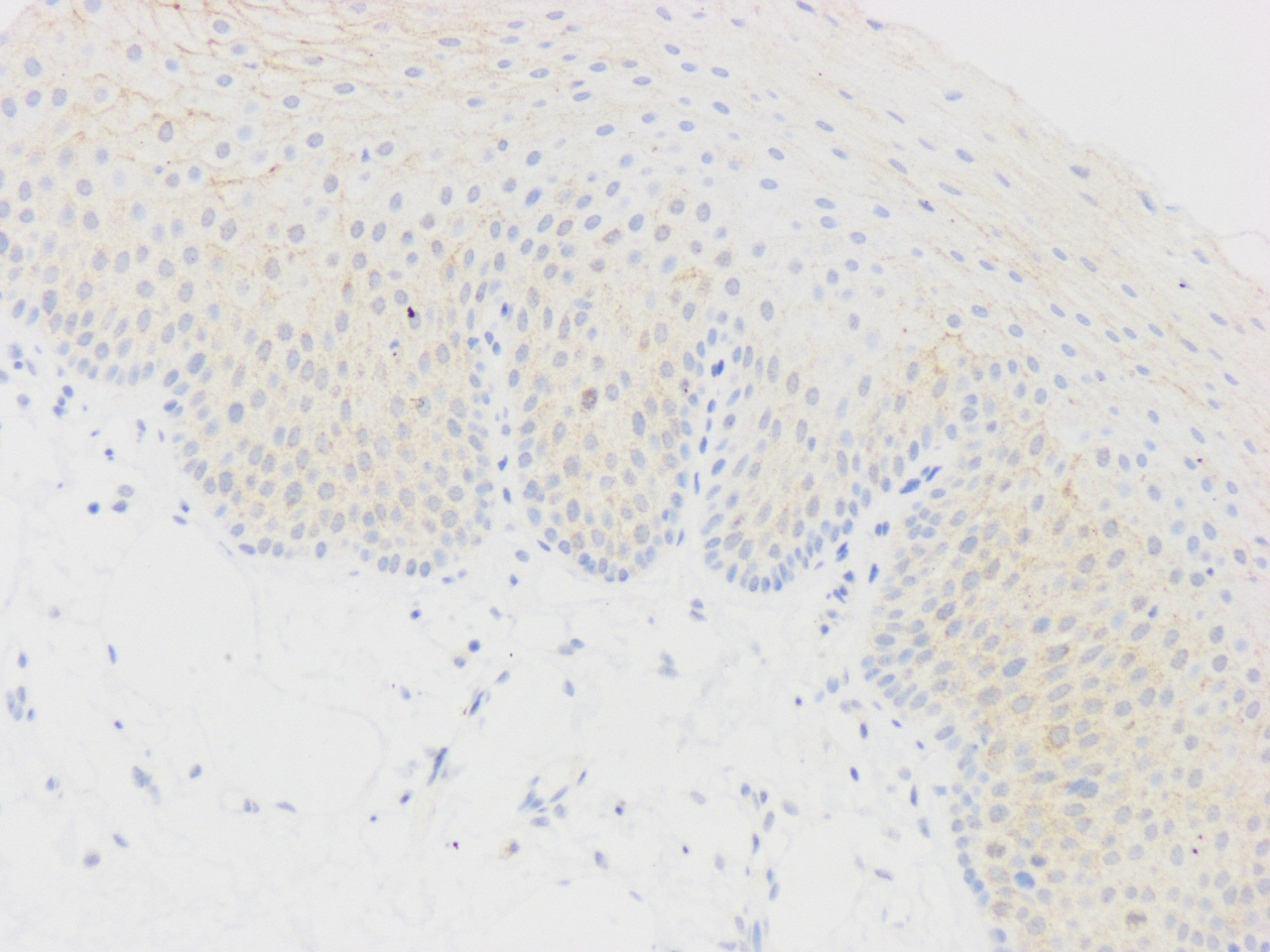

Supplement: Supplemental Information 1 — The data is showing that ACC expression in laryngocarcinoma by immunohistochemistry and westernblots. The 12-L-C-1 is represented by 12- laryngocarcinoma-cancer-1 while 12-L-N-1 is represented by 12-laryngocarcinoma-normal tissue-1. [file peerj-07-7037-s001.zip › Raw data 1/immunohistochemical images/12-L-N3 ACC.jpg]

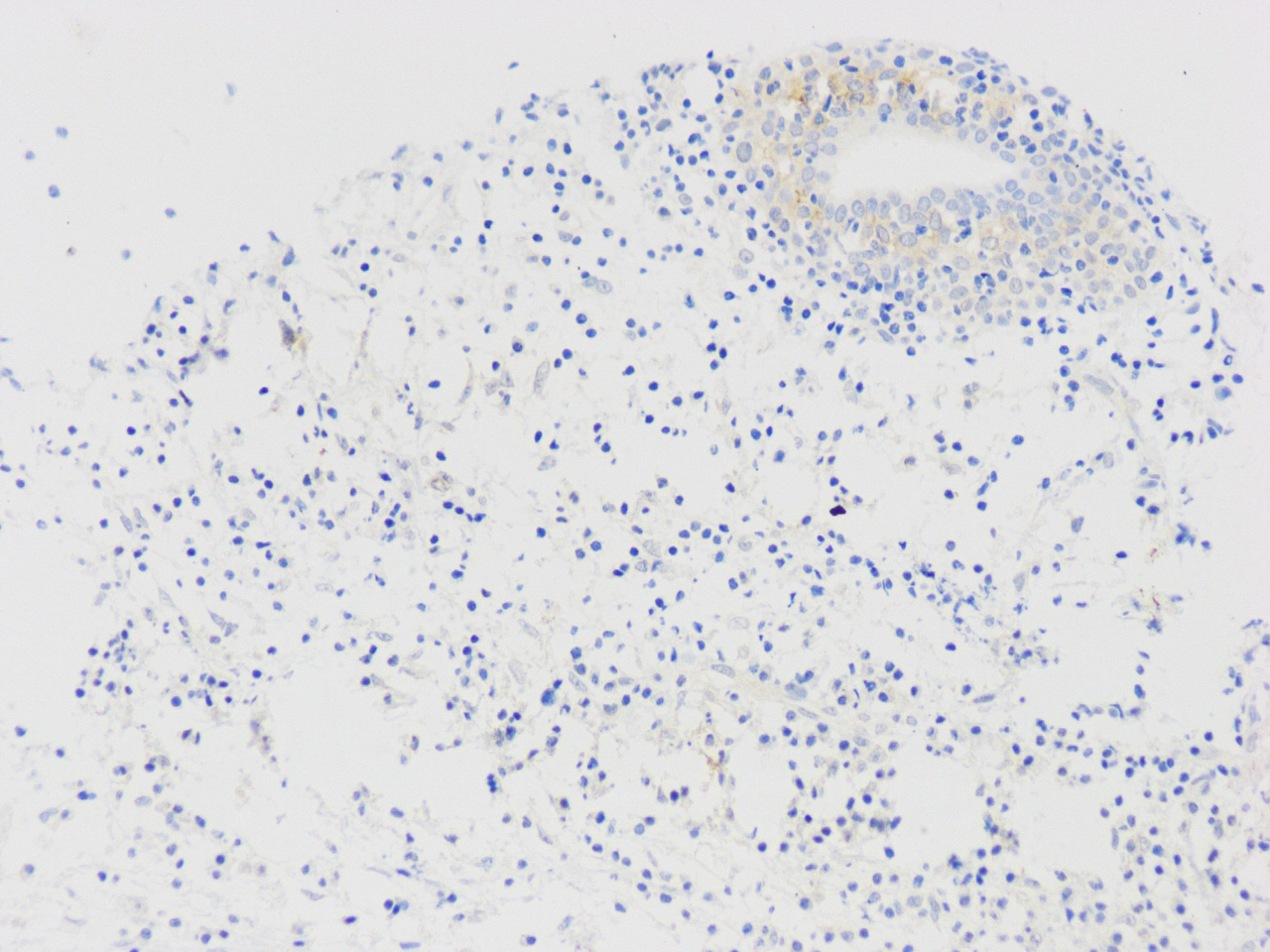

Supplement: Supplemental Information 1 — The data is showing that ACC expression in laryngocarcinoma by immunohistochemistry and westernblots. The 12-L-C-1 is represented by 12- laryngocarcinoma-cancer-1 while 12-L-N-1 is represented by 12-laryngocarcinoma-normal tissue-1. [file peerj-07-7037-s001.zip › Raw data 1/immunohistochemical images/12-L-N4 ACC.jpg]

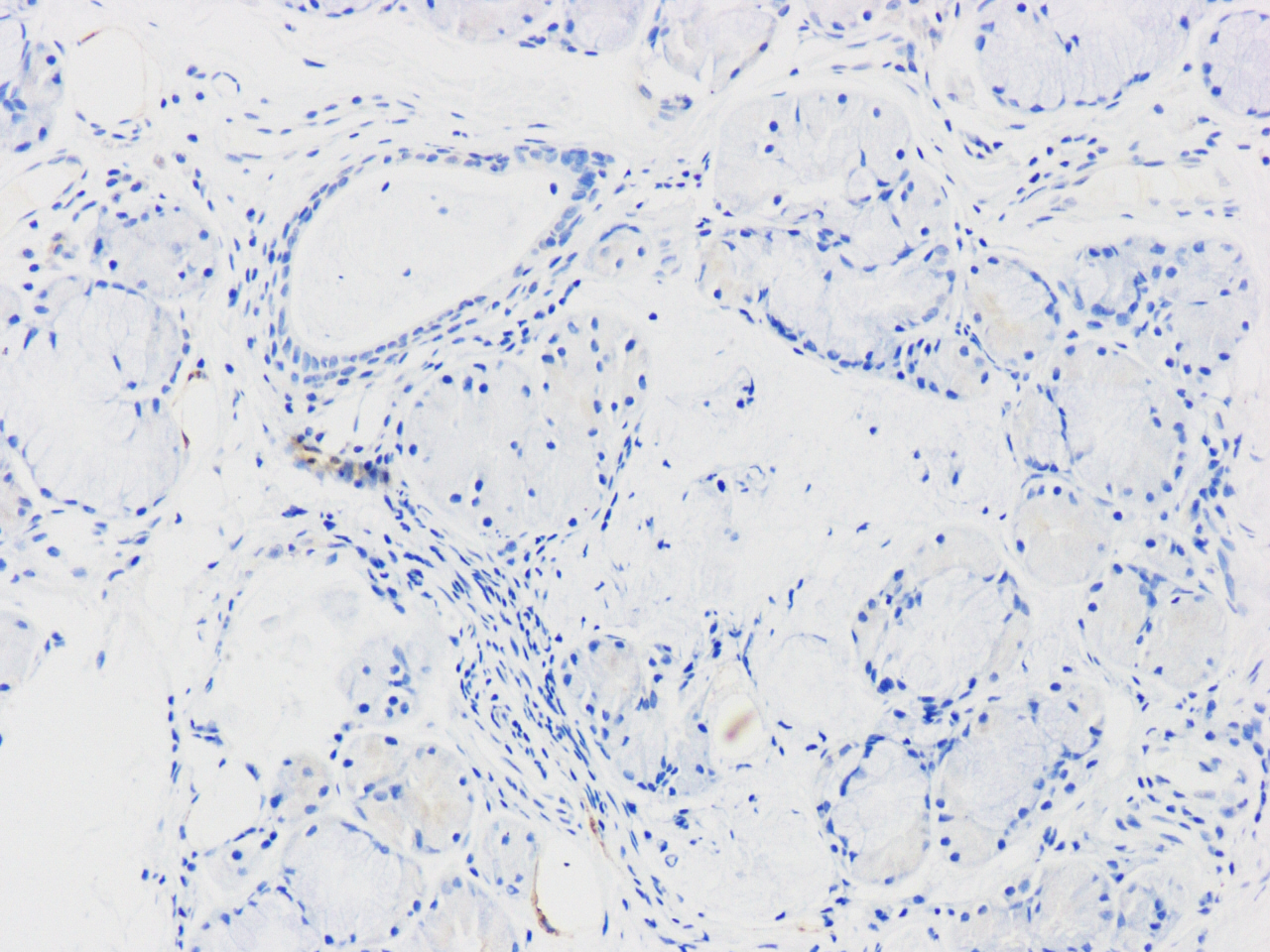

Supplement: Supplemental Information 1 — The data is showing that ACC expression in laryngocarcinoma by immunohistochemistry and westernblots. The 12-L-C-1 is represented by 12- laryngocarcinoma-cancer-1 while 12-L-N-1 is represented by 12-laryngocarcinoma-normal tissue-1. [file peerj-07-7037-s001.zip › Raw data 1/immunohistochemical images/12-L-N5 ACC.jpg]

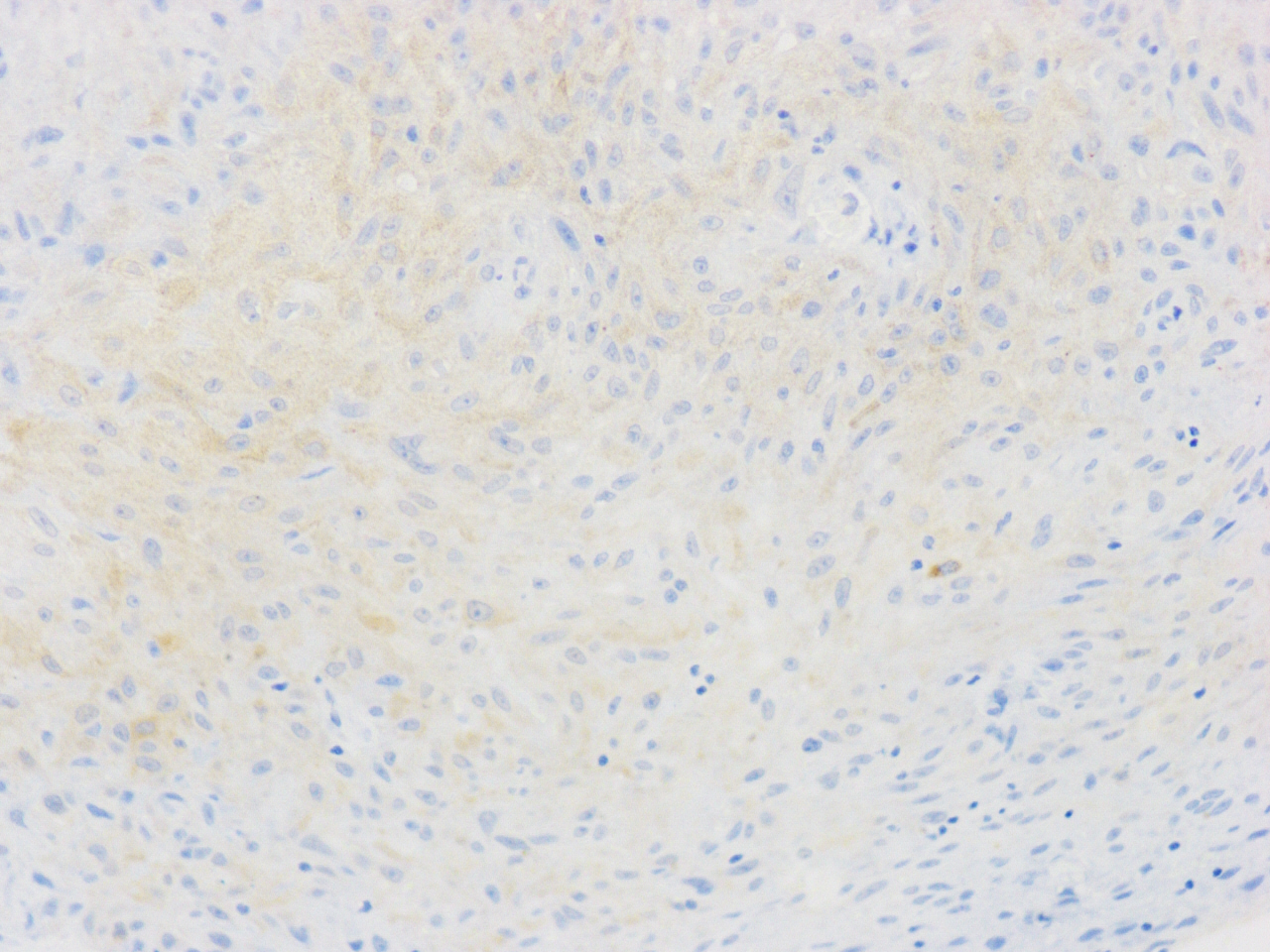

Supplement: Supplemental Information 1 — The data is showing that ACC expression in laryngocarcinoma by immunohistochemistry and westernblots. The 12-L-C-1 is represented by 12- laryngocarcinoma-cancer-1 while 12-L-N-1 is represented by 12-laryngocarcinoma-normal tissue-1. [file peerj-07-7037-s001.zip › Raw data 1/immunohistochemical images/12-L-N6 ACC.jpg]

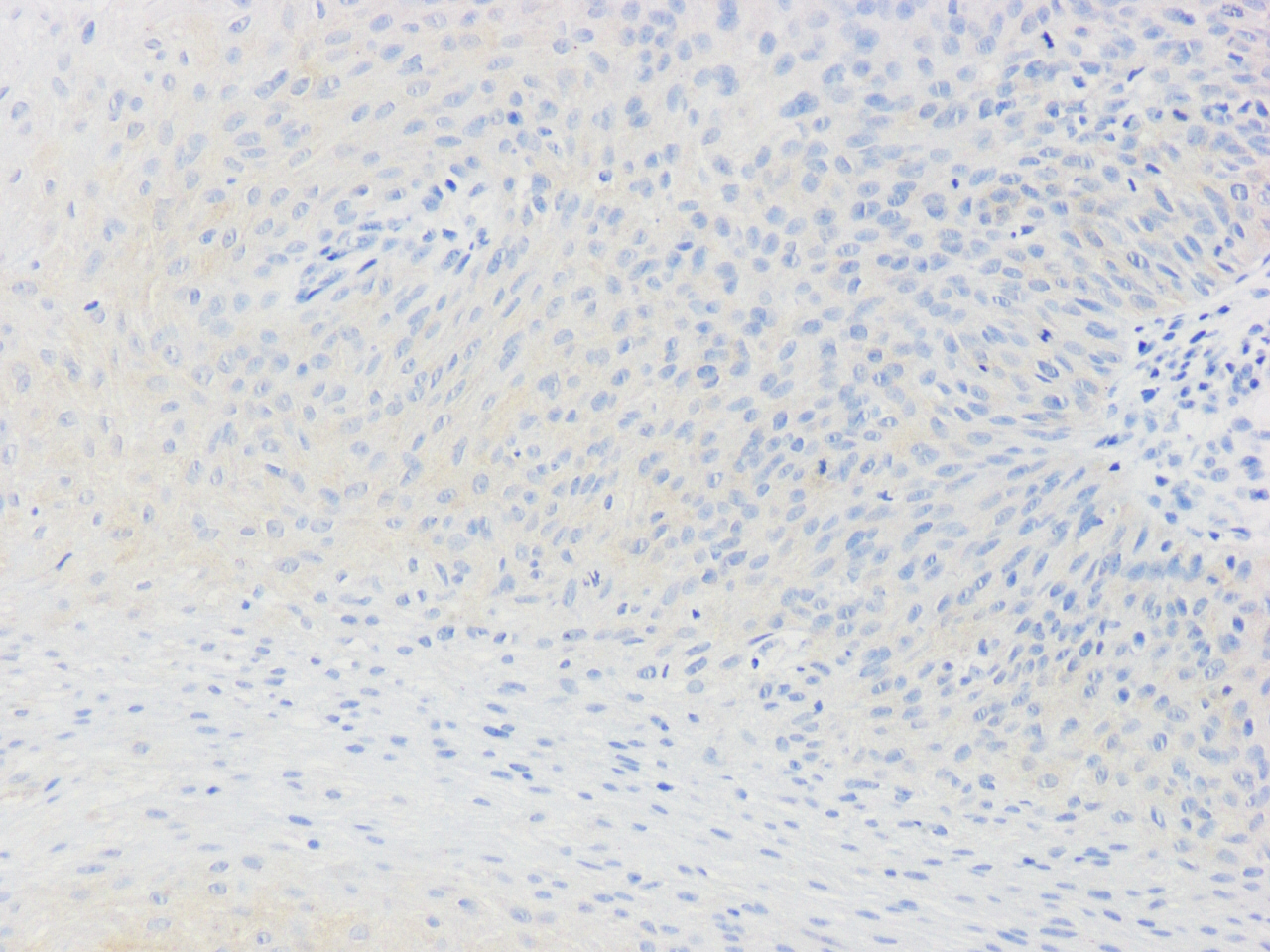

Supplement: Supplemental Information 1 — The data is showing that ACC expression in laryngocarcinoma by immunohistochemistry and westernblots. The 12-L-C-1 is represented by 12- laryngocarcinoma-cancer-1 while 12-L-N-1 is represented by 12-laryngocarcinoma-normal tissue-1. [file peerj-07-7037-s001.zip › Raw data 1/immunohistochemical images/12-L-N7 ACC.jpg]

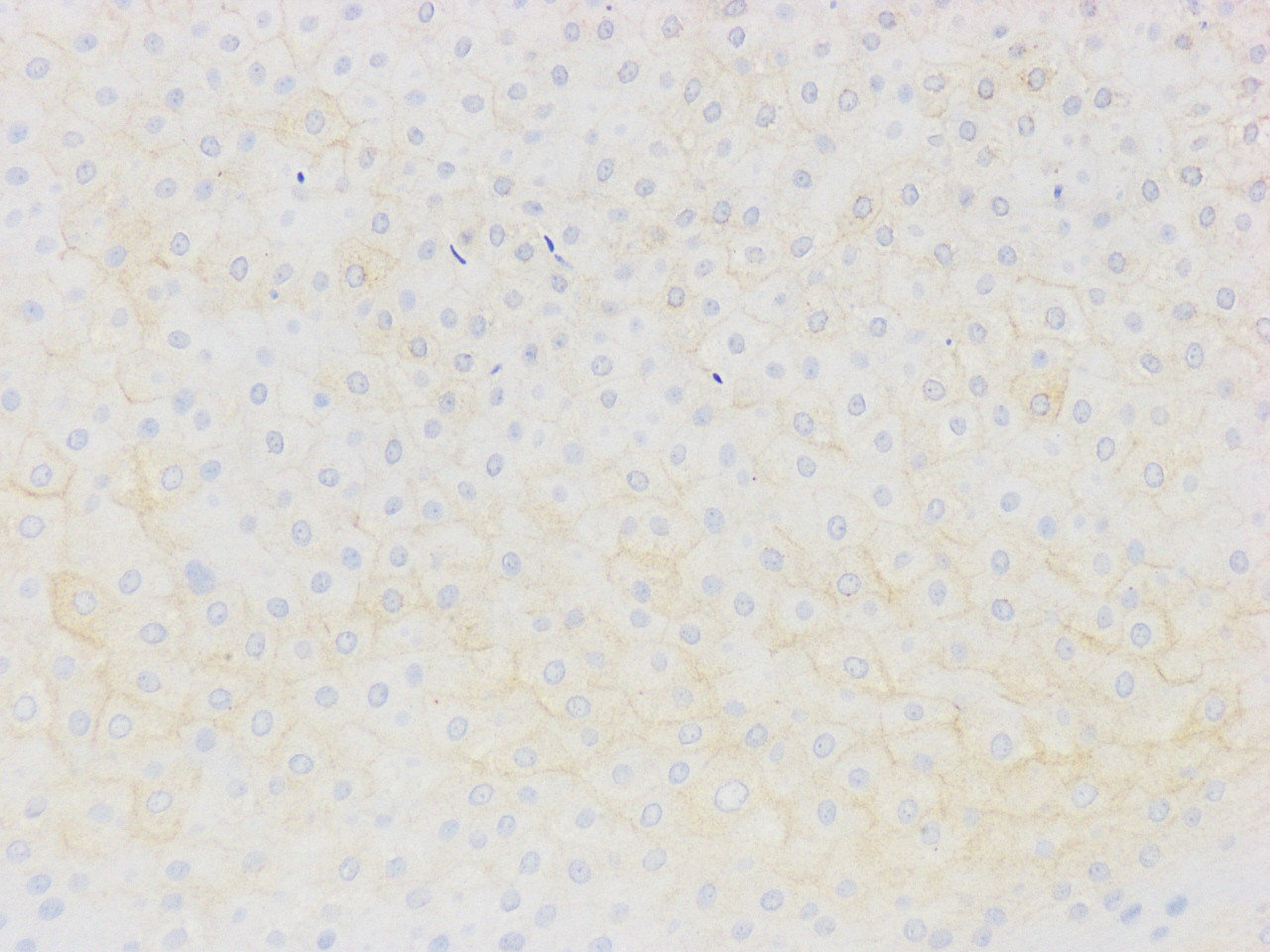

Supplement: Supplemental Information 1 — The data is showing that ACC expression in laryngocarcinoma by immunohistochemistry and westernblots. The 12-L-C-1 is represented by 12- laryngocarcinoma-cancer-1 while 12-L-N-1 is represented by 12-laryngocarcinoma-normal tissue-1. [file peerj-07-7037-s001.zip › Raw data 1/immunohistochemical images/12-L-N9 ACC.jpg]

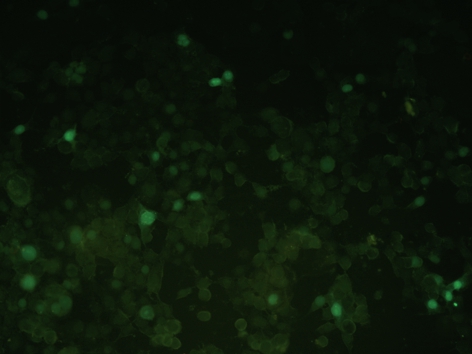

Supplement: Supplemental Information 2 — The data is showing full-length uncropped blots (Figure 3, Figure 4) and apoptosis in FADU by TUNEL assays. [file peerj-07-7037-s002.zip › Raw data 2/Tunel analysis/ACC siRNA 1.jpg]

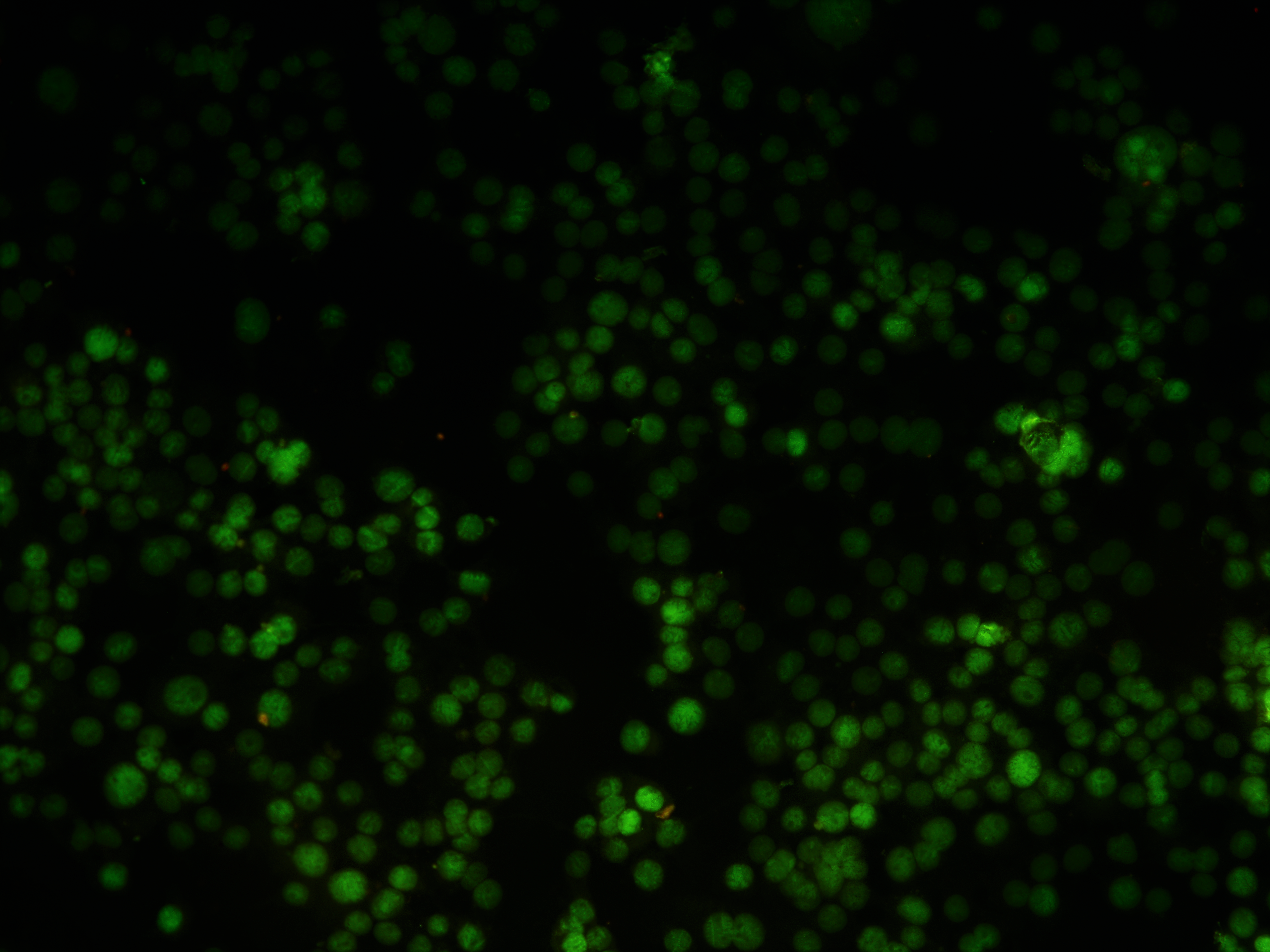

Supplement: Supplemental Information 2 — The data is showing full-length uncropped blots (Figure 3, Figure 4) and apoptosis in FADU by TUNEL assays. [file peerj-07-7037-s002.zip › Raw data 2/Tunel analysis/ACC siRNA 2.tif]

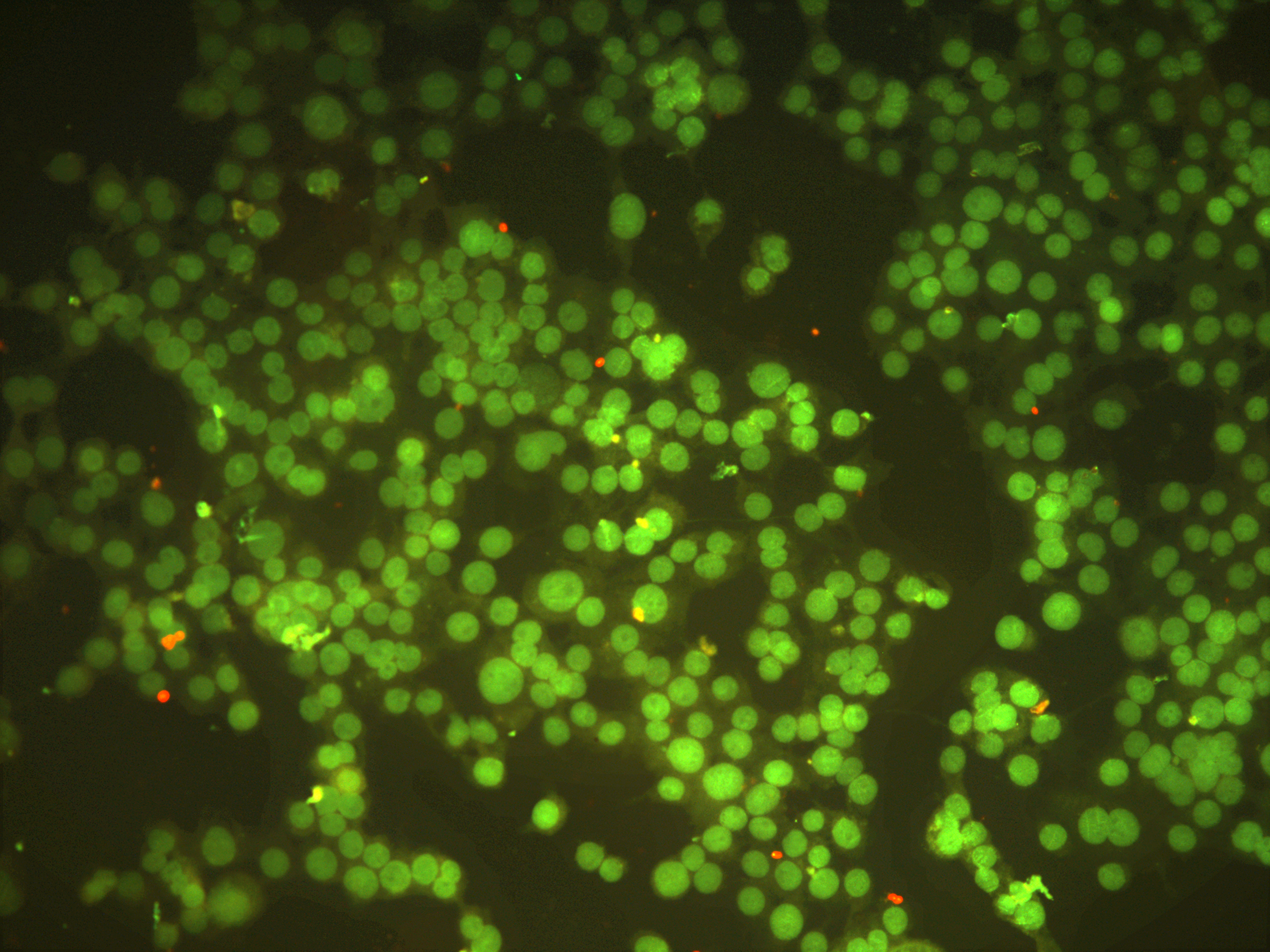

Supplement: Supplemental Information 2 — The data is showing full-length uncropped blots (Figure 3, Figure 4) and apoptosis in FADU by TUNEL assays. [file peerj-07-7037-s002.zip › Raw data 2/Tunel analysis/Scrambled siRNA 2.tif]

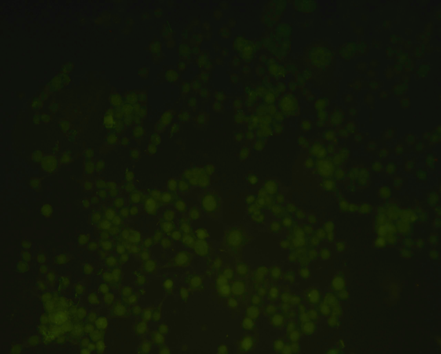

Supplement: Supplemental Information 2 — The data is showing full-length uncropped blots (Figure 3, Figure 4) and apoptosis in FADU by TUNEL assays. [file peerj-07-7037-s002.zip › Raw data 2/Tunel analysis/Scrambled siRNA1.tif]

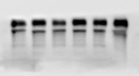

Supplement: Supplemental Information 2 — The data is showing full-length uncropped blots (Figure 3, Figure 4) and apoptosis in FADU by TUNEL assays. [file peerj-07-7037-s002.zip › Raw data 2/Westernblot images/ACC expression in NP69 FADU/Acc(NC- ACCsiRNA).tif]

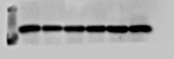

Supplement: Supplemental Information 2 — The data is showing full-length uncropped blots (Figure 3, Figure 4) and apoptosis in FADU by TUNEL assays. [file peerj-07-7037-s002.zip › Raw data 2/Westernblot images/ACC expression in NP69 FADU/β-tubulin(NC- ACCsiRNA).tif]

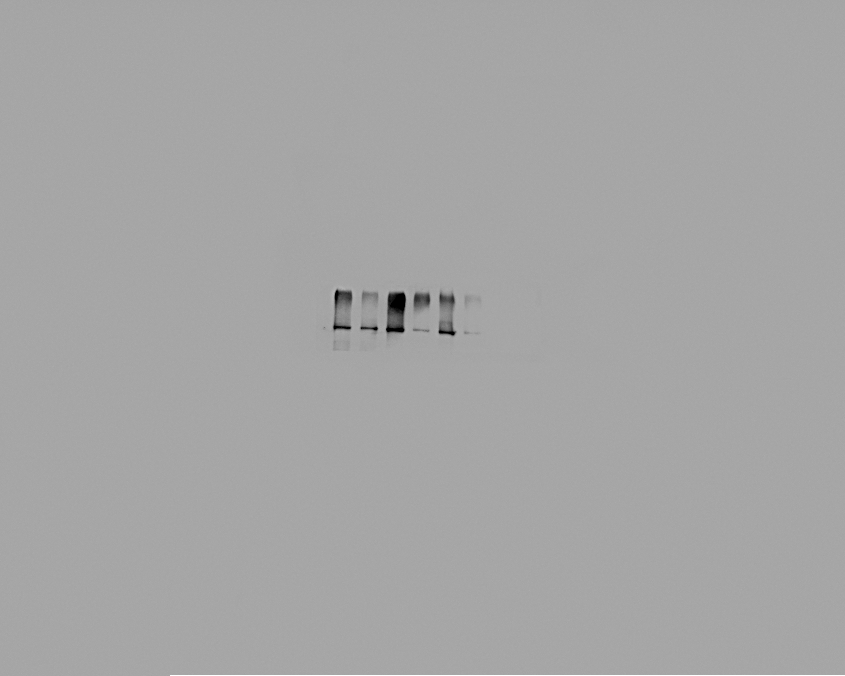

Supplement: Supplemental Information 2 — The data is showing full-length uncropped blots (Figure 3, Figure 4) and apoptosis in FADU by TUNEL assays. [file peerj-07-7037-s002.zip › Raw data 2/Westernblot images/ACC Knockdown in FADU/ACC(NC- ACCsiRNA).tif]

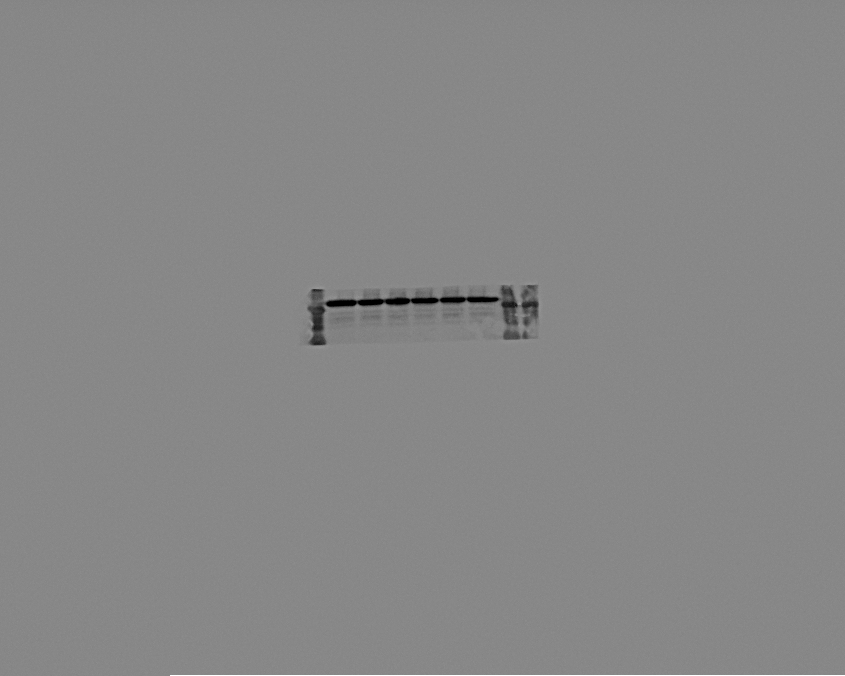

Supplement: Supplemental Information 2 — The data is showing full-length uncropped blots (Figure 3, Figure 4) and apoptosis in FADU by TUNEL assays. [file peerj-07-7037-s002.zip › Raw data 2/Westernblot images/ACC Knockdown in FADU/β-tubulin(NC-ACCsiRNA).tif]

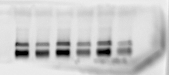

Supplement: Supplemental Information 2 — The data is showing full-length uncropped blots (Figure 3, Figure 4) and apoptosis in FADU by TUNEL assays. [file peerj-07-7037-s002.zip › Raw data 2/Westernblot images/ACC Knockdown in NP69/ACC(NC- ACCsiRNA).tif]

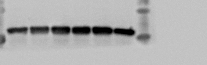

Supplement: Supplemental Information 2 — The data is showing full-length uncropped blots (Figure 3, Figure 4) and apoptosis in FADU by TUNEL assays. [file peerj-07-7037-s002.zip › Raw data 2/Westernblot images/ACC Knockdown in NP69/GAPDH(NC- ACCsiRNA ).tif]

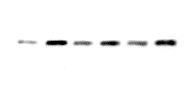

Supplement: Supplemental Information 2 — The data is showing full-length uncropped blots (Figure 3, Figure 4) and apoptosis in FADU by TUNEL assays. [file peerj-07-7037-s002.zip › Raw data 2/Westernblot images/Apoptosis proteins in FADU/bax(NC- ACCsiRNA).tif]

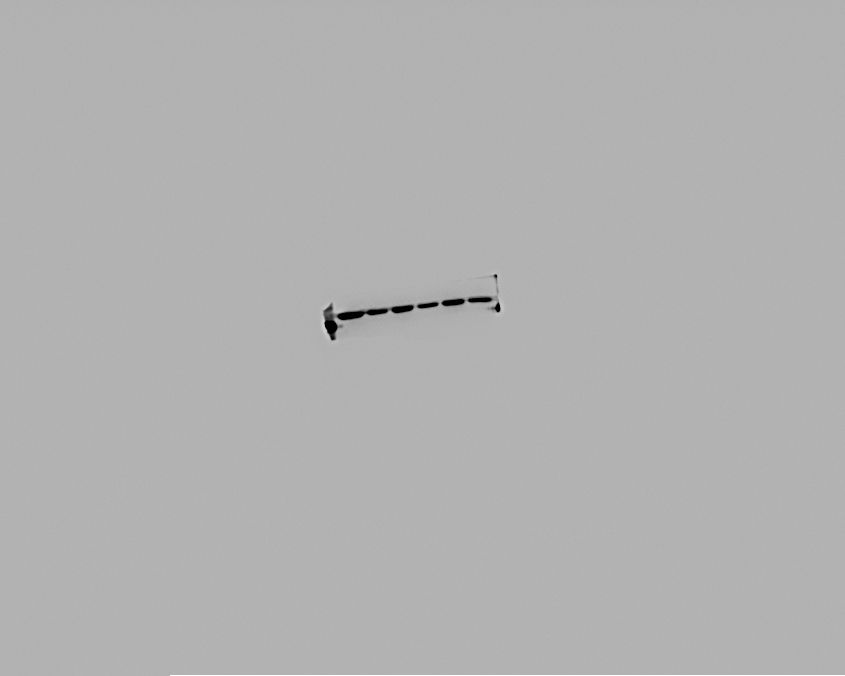

Supplement: Supplemental Information 2 — The data is showing full-length uncropped blots (Figure 3, Figure 4) and apoptosis in FADU by TUNEL assays. [file peerj-07-7037-s002.zip › Raw data 2/Westernblot images/Apoptosis proteins in FADU/bcl-2(NC- ACCsiRNA).tif]

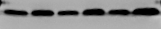

Supplement: Supplemental Information 2 — The data is showing full-length uncropped blots (Figure 3, Figure 4) and apoptosis in FADU by TUNEL assays. [file peerj-07-7037-s002.zip › Raw data 2/Westernblot images/Apoptosis proteins in FADU/casepase3(NC- ACCsiRNA).tif]

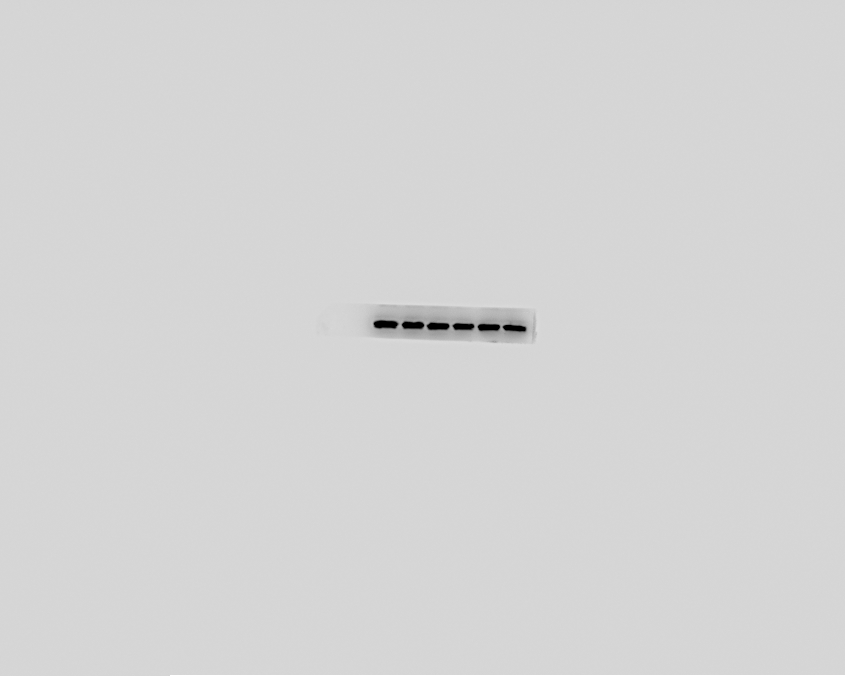

Supplement: Supplemental Information 2 — The data is showing full-length uncropped blots (Figure 3, Figure 4) and apoptosis in FADU by TUNEL assays. [file peerj-07-7037-s002.zip › Raw data 2/Westernblot images/Apoptosis proteins in FADU/β-tubulin(NC- ACCsiRNA).tif]

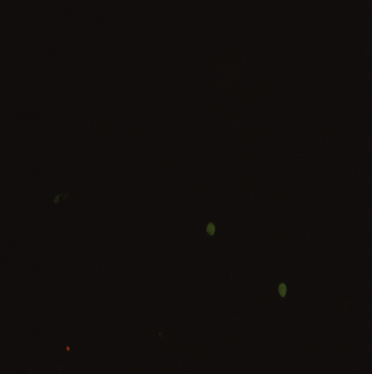

Supplement: Supplemental Information 3 — Images showing apoptotic NP69 cells transfected with scrambled siRNA or ACC2 siRNA. [file peerj-07-7037-s003.zip › Raw data-3/NP69 cell apoptosis results/NC 1.png]

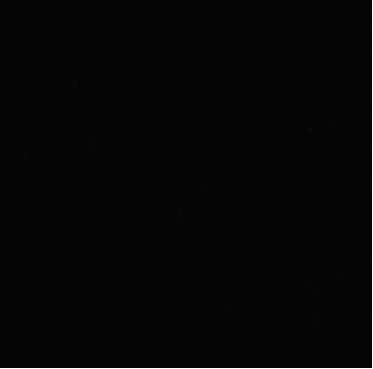

Supplement: Supplemental Information 3 — Images showing apoptotic NP69 cells transfected with scrambled siRNA or ACC2 siRNA. [file peerj-07-7037-s003.zip › Raw data-3/NP69 cell apoptosis results/NC 2.png]

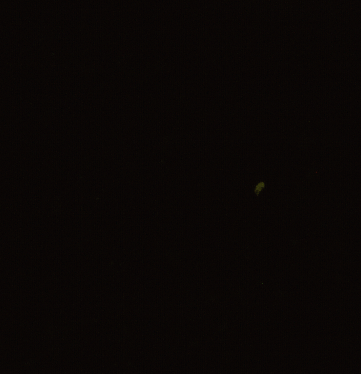

Supplement: Supplemental Information 3 — Images showing apoptotic NP69 cells transfected with scrambled siRNA or ACC2 siRNA. [file peerj-07-7037-s003.zip › Raw data-3/NP69 cell apoptosis results/NC 3.png]

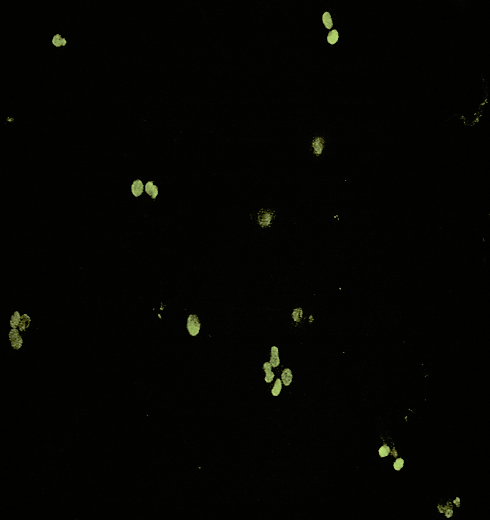

Supplement: Supplemental Information 3 — Images showing apoptotic NP69 cells transfected with scrambled siRNA or ACC2 siRNA. [file peerj-07-7037-s003.zip › Raw data-3/NP69 cell apoptosis results/NP69 apoptosis cell 1.png]

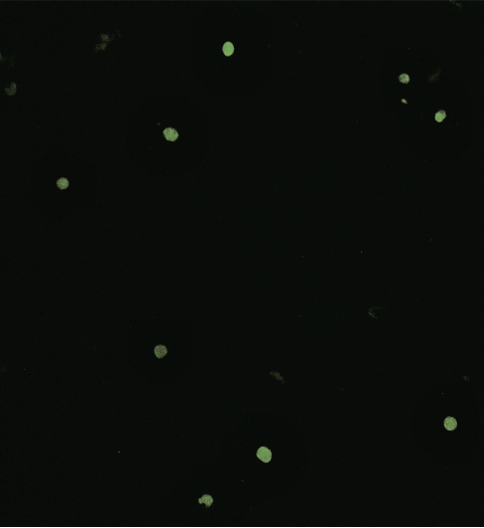

Supplement: Supplemental Information 3 — Images showing apoptotic NP69 cells transfected with scrambled siRNA or ACC2 siRNA. [file peerj-07-7037-s003.zip › Raw data-3/NP69 cell apoptosis results/NP69 apoptosis cell 2.png]

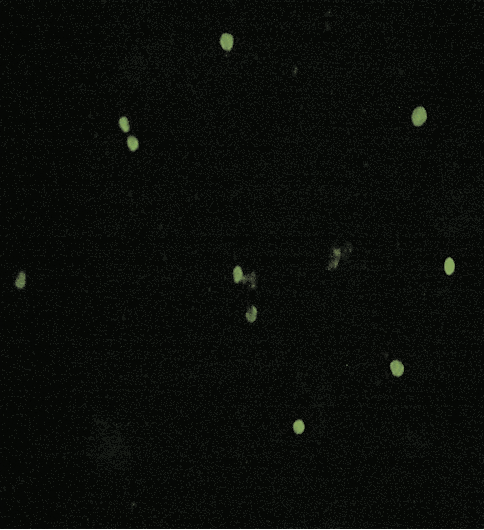

Supplement: Supplemental Information 3 — Images showing apoptotic NP69 cells transfected with scrambled siRNA or ACC2 siRNA. [file peerj-07-7037-s003.zip › Raw data-3/NP69 cell apoptosis results/NP69 apoptosis cell 3.png]

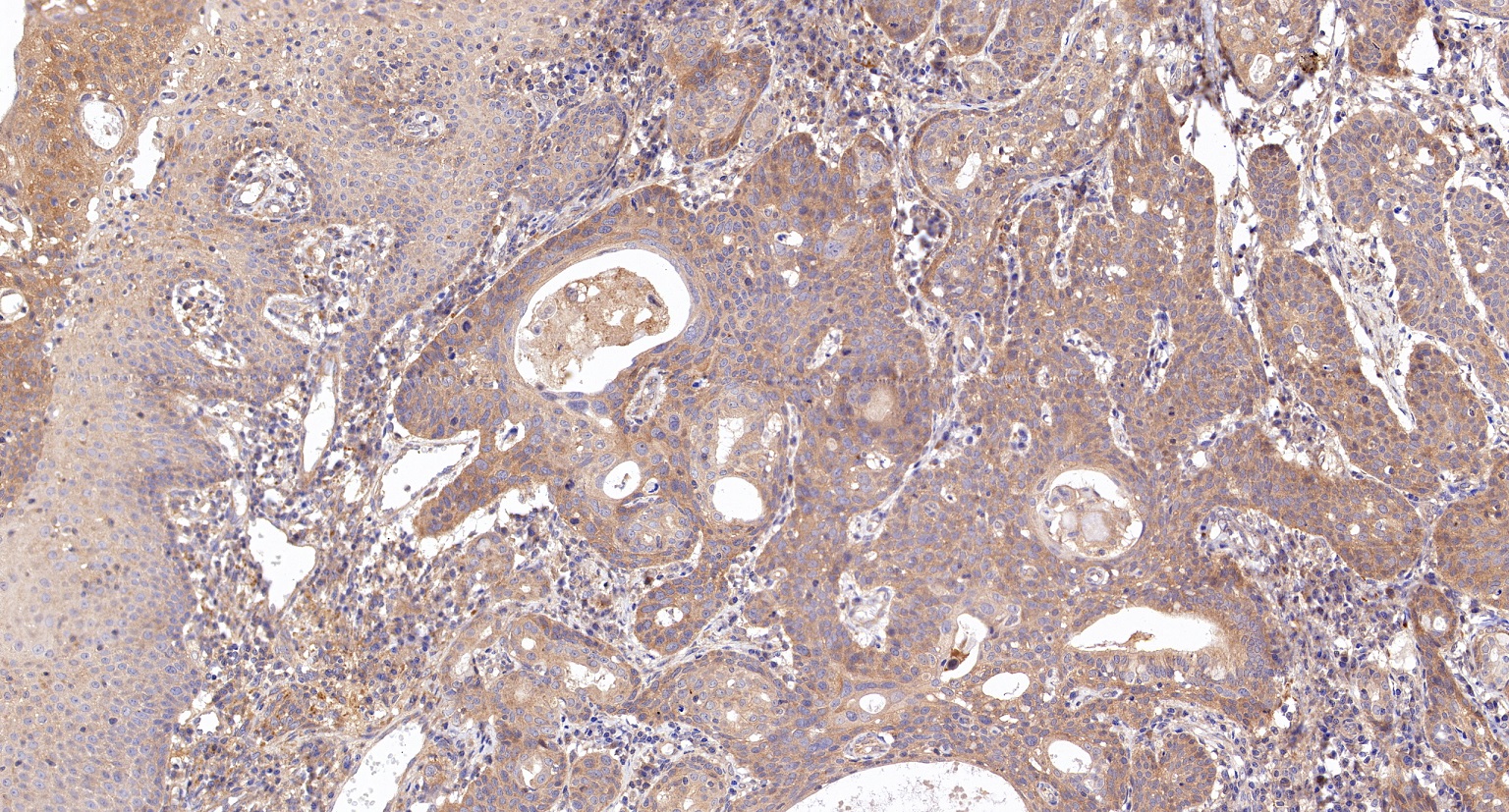

Supplement: Supplemental Information 4 — Immunohistochemical images showing p-ACC immunostaining in the normal tissue adjacent to laryngocarcinoma tissue. [file peerj-07-7037-s004.zip › Raw data-4/p-ACC expression in HNSCC (immunohistochemical analysis)/12-L-C20-pACC.jpg]

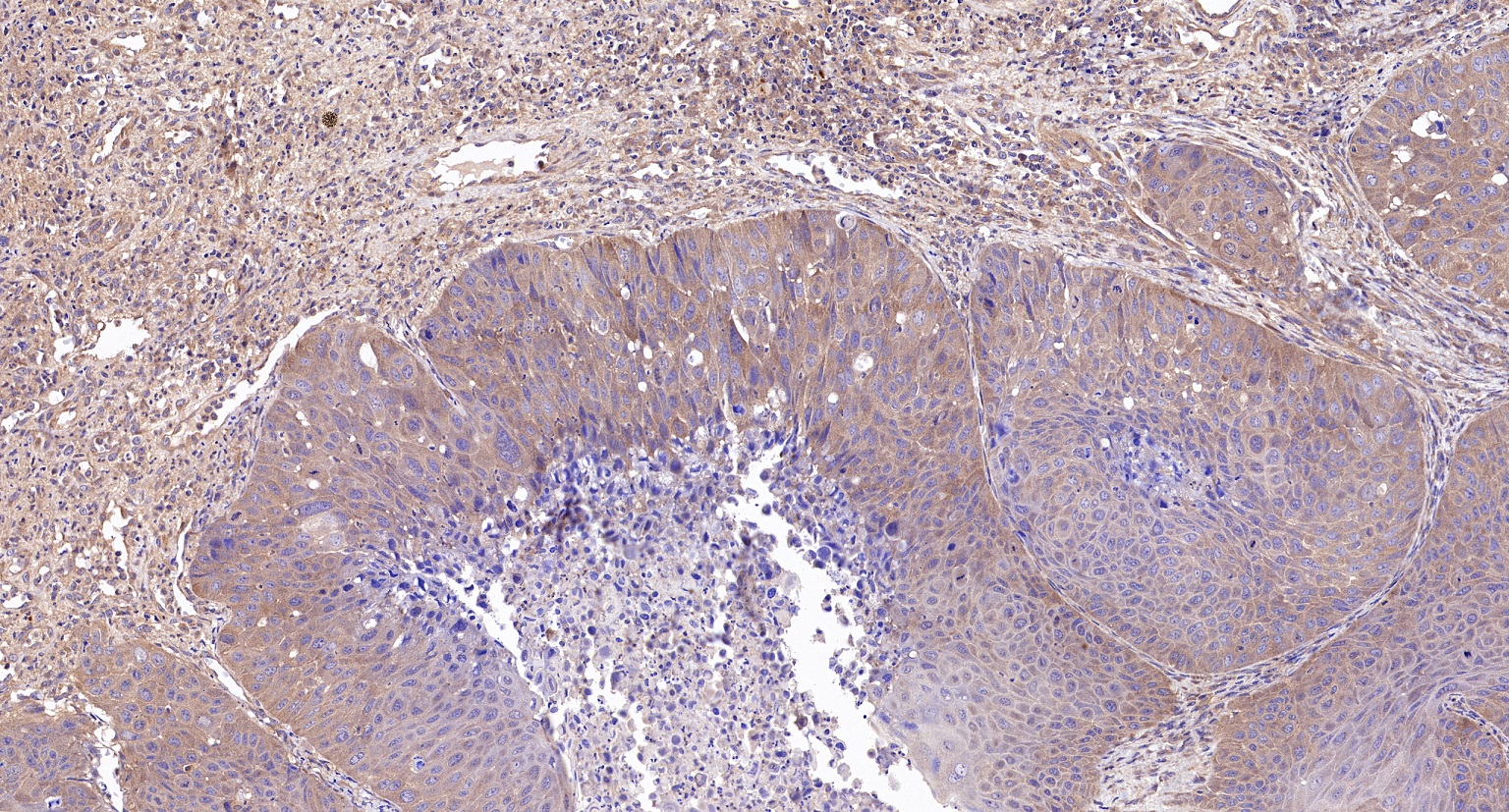

Supplement: Supplemental Information 4 — Immunohistochemical images showing p-ACC immunostaining in the normal tissue adjacent to laryngocarcinoma tissue. [file peerj-07-7037-s004.zip › Raw data-4/p-ACC expression in HNSCC (immunohistochemical analysis)/12-L-C22-pACC.jpg]

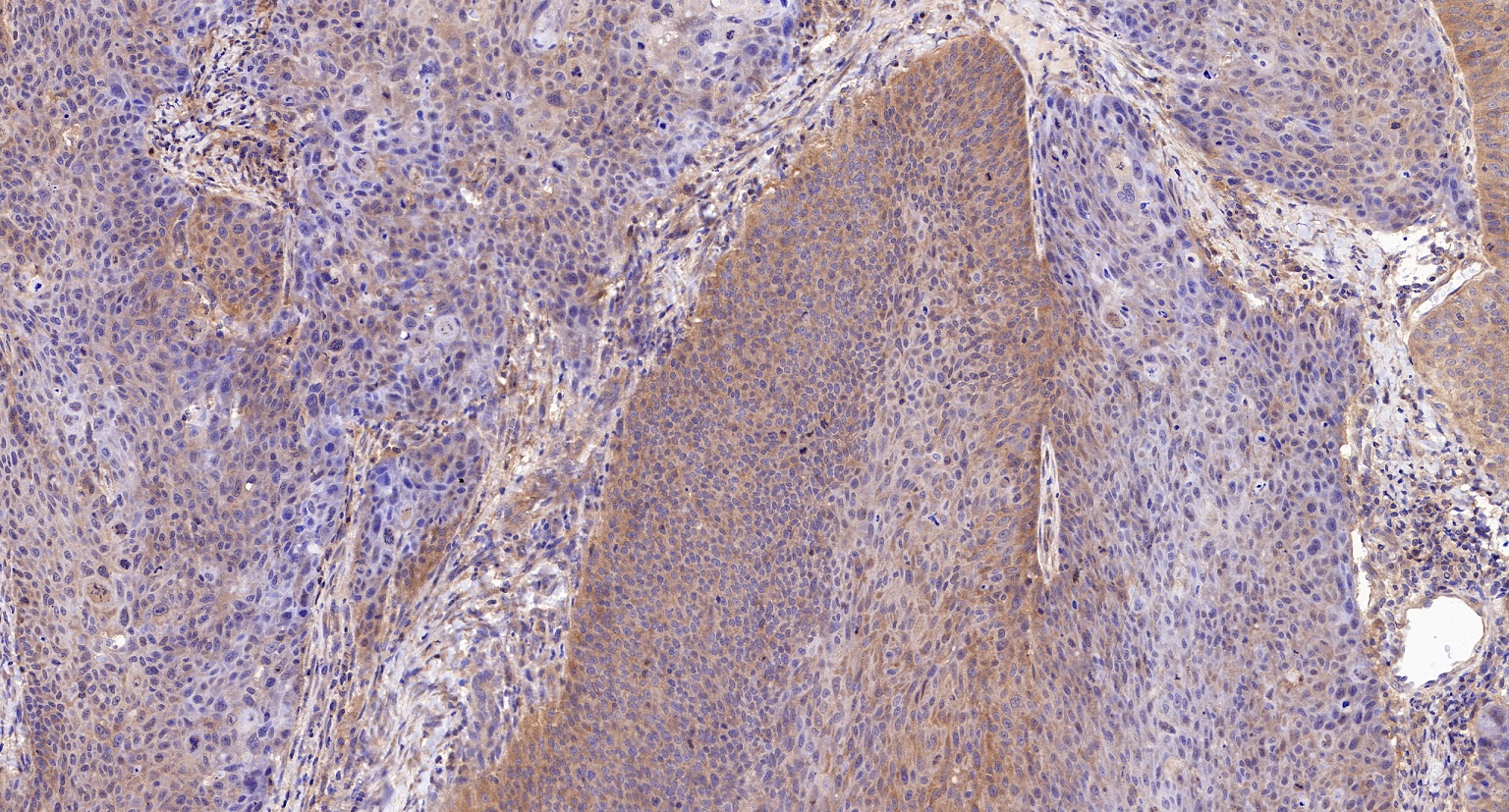

Supplement: Supplemental Information 4 — Immunohistochemical images showing p-ACC immunostaining in the normal tissue adjacent to laryngocarcinoma tissue. [file peerj-07-7037-s004.zip › Raw data-4/p-ACC expression in HNSCC (immunohistochemical analysis)/12-L-C30-pACC.jpg]

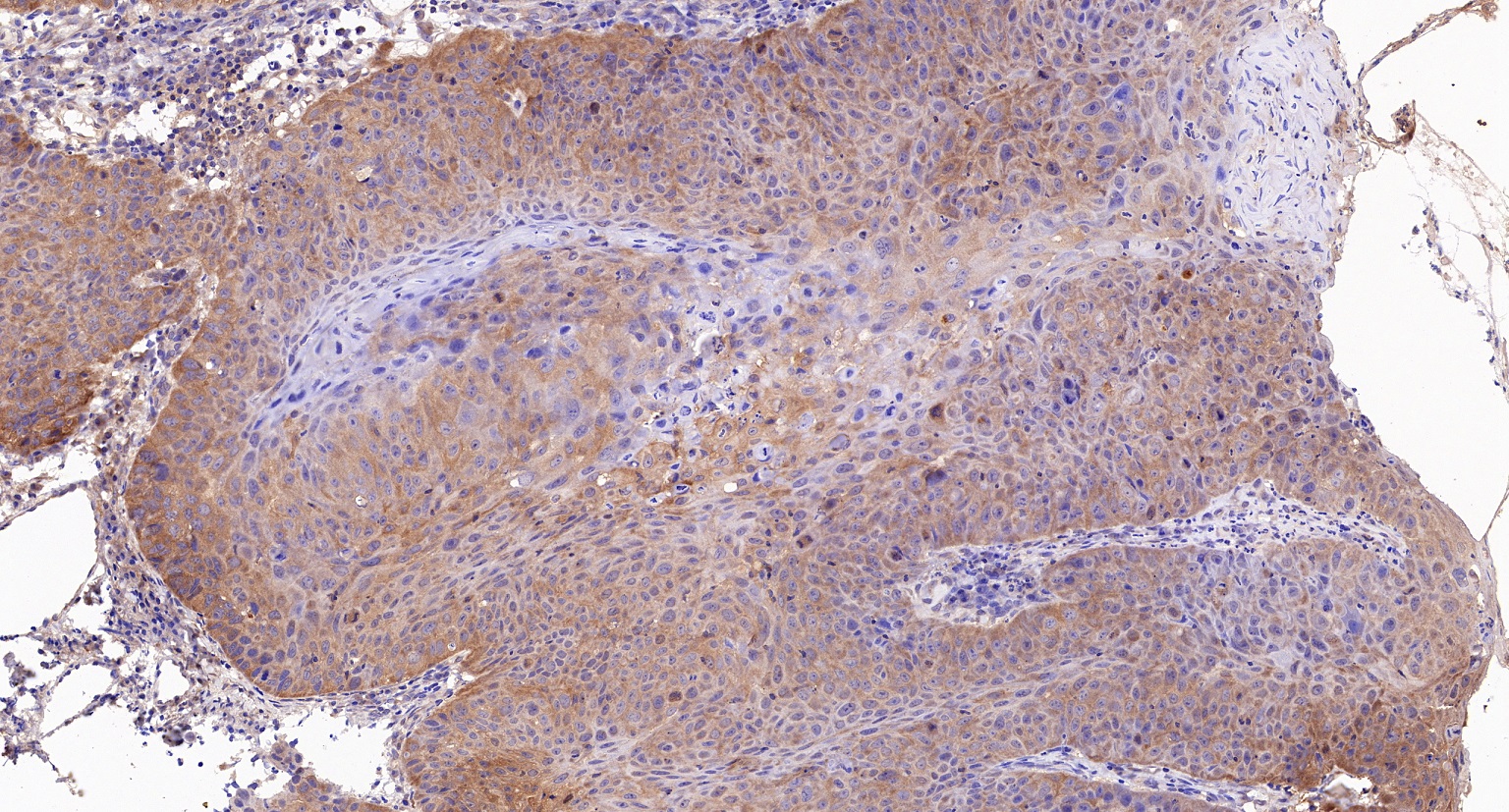

Supplement: Supplemental Information 4 — Immunohistochemical images showing p-ACC immunostaining in the normal tissue adjacent to laryngocarcinoma tissue. [file peerj-07-7037-s004.zip › Raw data-4/p-ACC expression in HNSCC (immunohistochemical analysis)/12-L-C34-pACC.jpg]

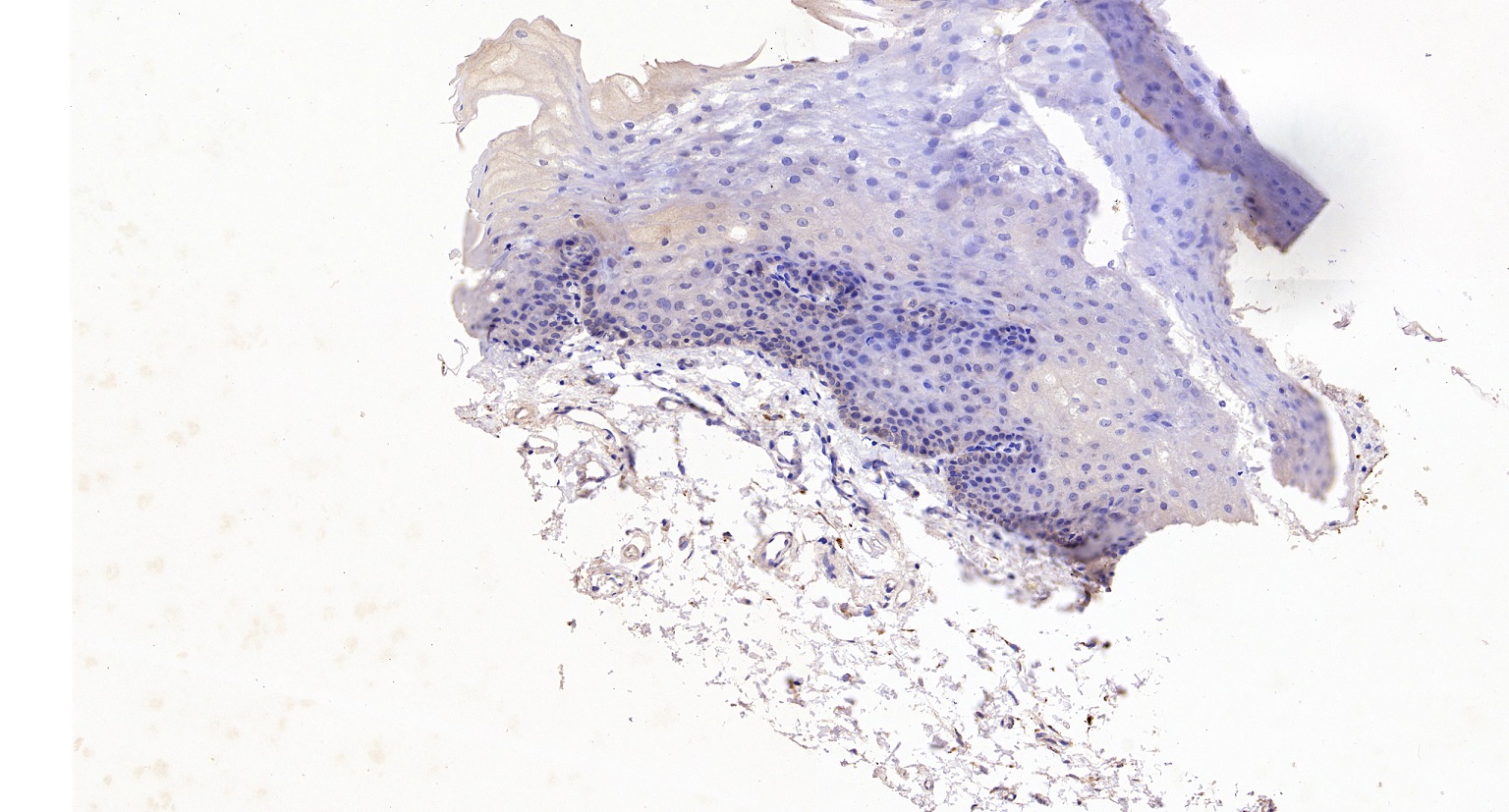

Supplement: Supplemental Information 4 — Immunohistochemical images showing p-ACC immunostaining in the normal tissue adjacent to laryngocarcinoma tissue. [file peerj-07-7037-s004.zip › Raw data-4/p-ACC expression in HNSCC (immunohistochemical analysis)/12-L-N20-pACC.jpg]

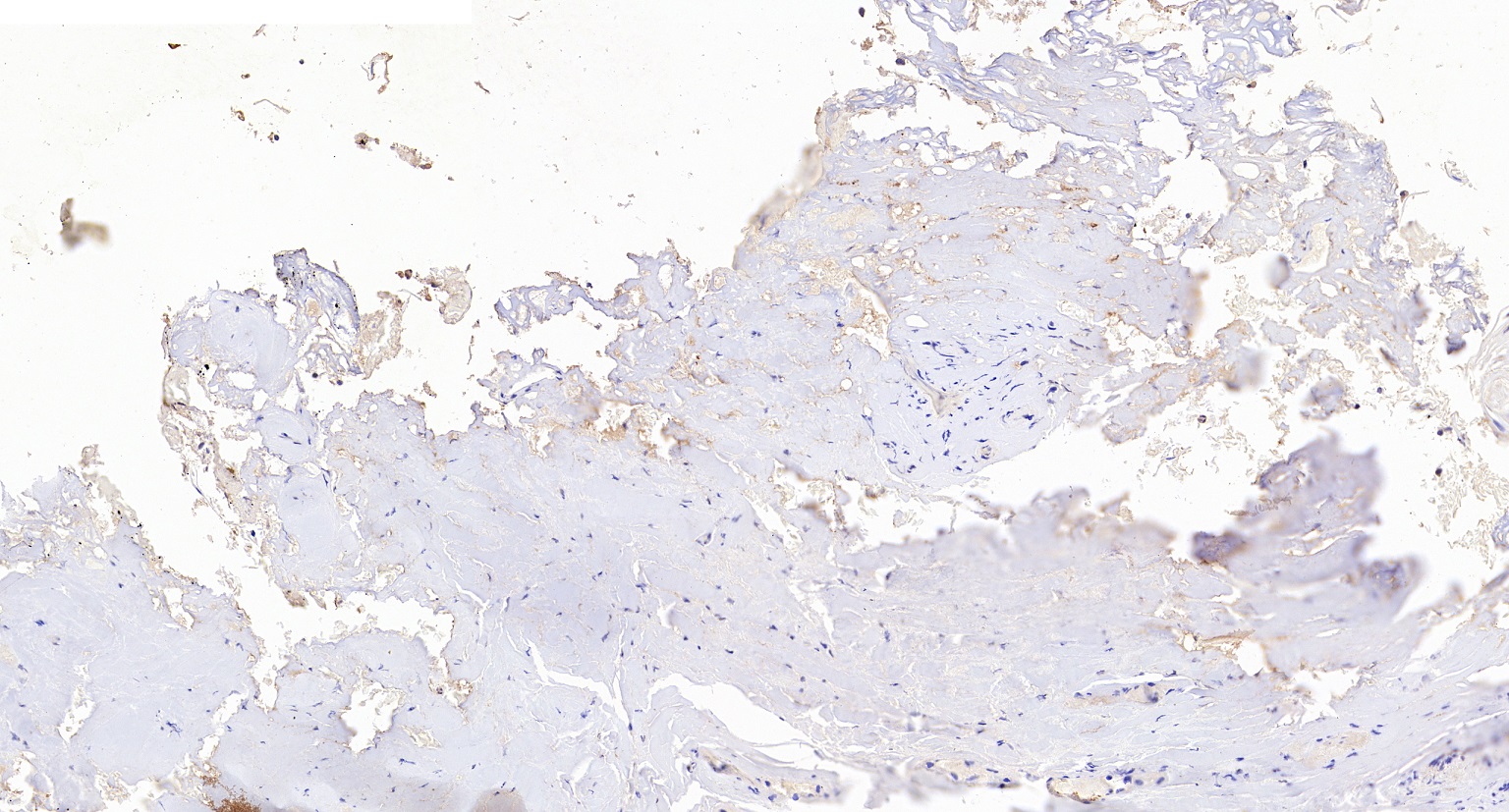

Supplement: Supplemental Information 4 — Immunohistochemical images showing p-ACC immunostaining in the normal tissue adjacent to laryngocarcinoma tissue. [file peerj-07-7037-s004.zip › Raw data-4/p-ACC expression in HNSCC (immunohistochemical analysis)/12-L-N22-pACC.jpg]

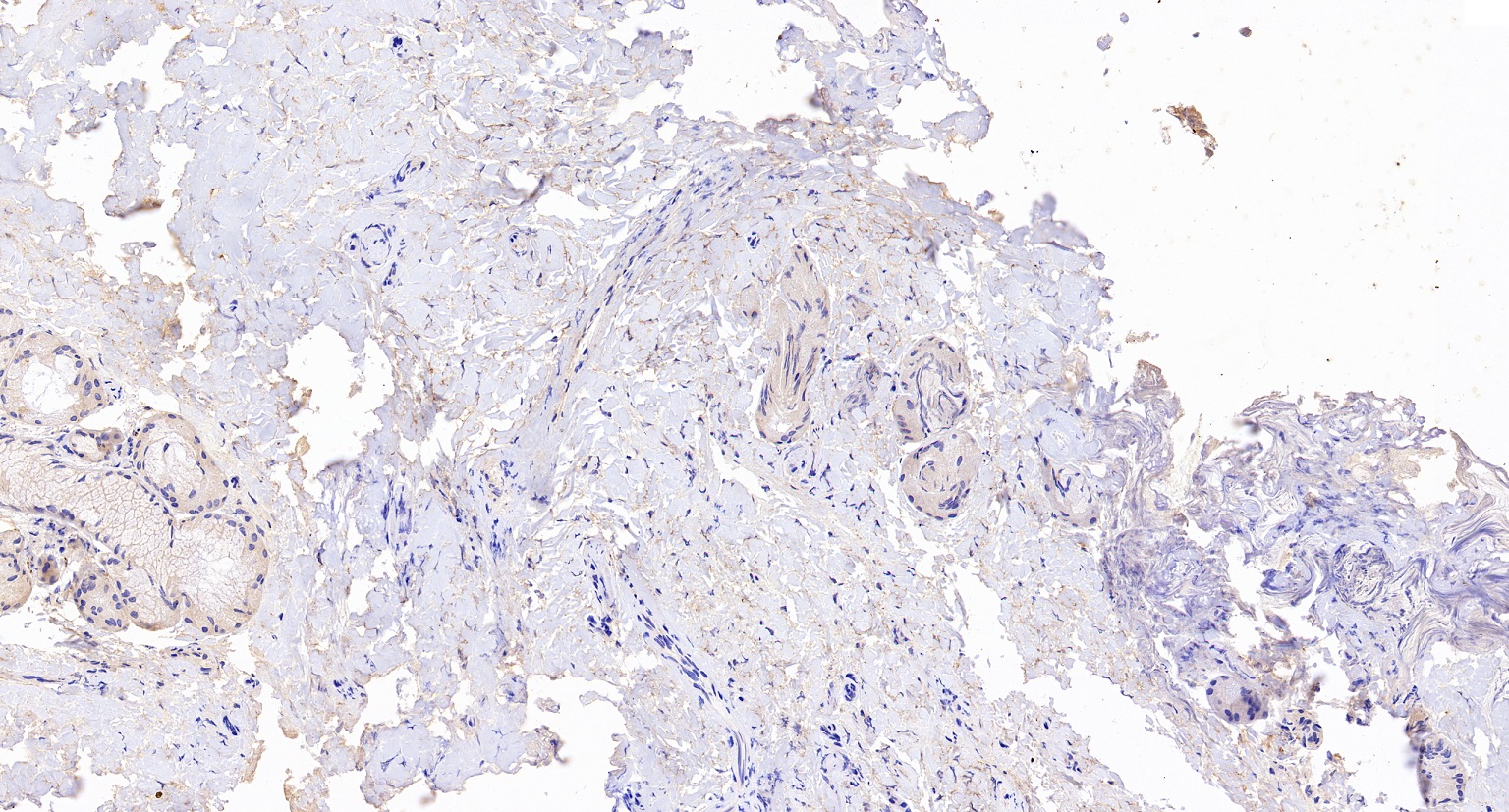

Supplement: Supplemental Information 4 — Immunohistochemical images showing p-ACC immunostaining in the normal tissue adjacent to laryngocarcinoma tissue. [file peerj-07-7037-s004.zip › Raw data-4/p-ACC expression in HNSCC (immunohistochemical analysis)/12-L-N30-pACC.jpg]

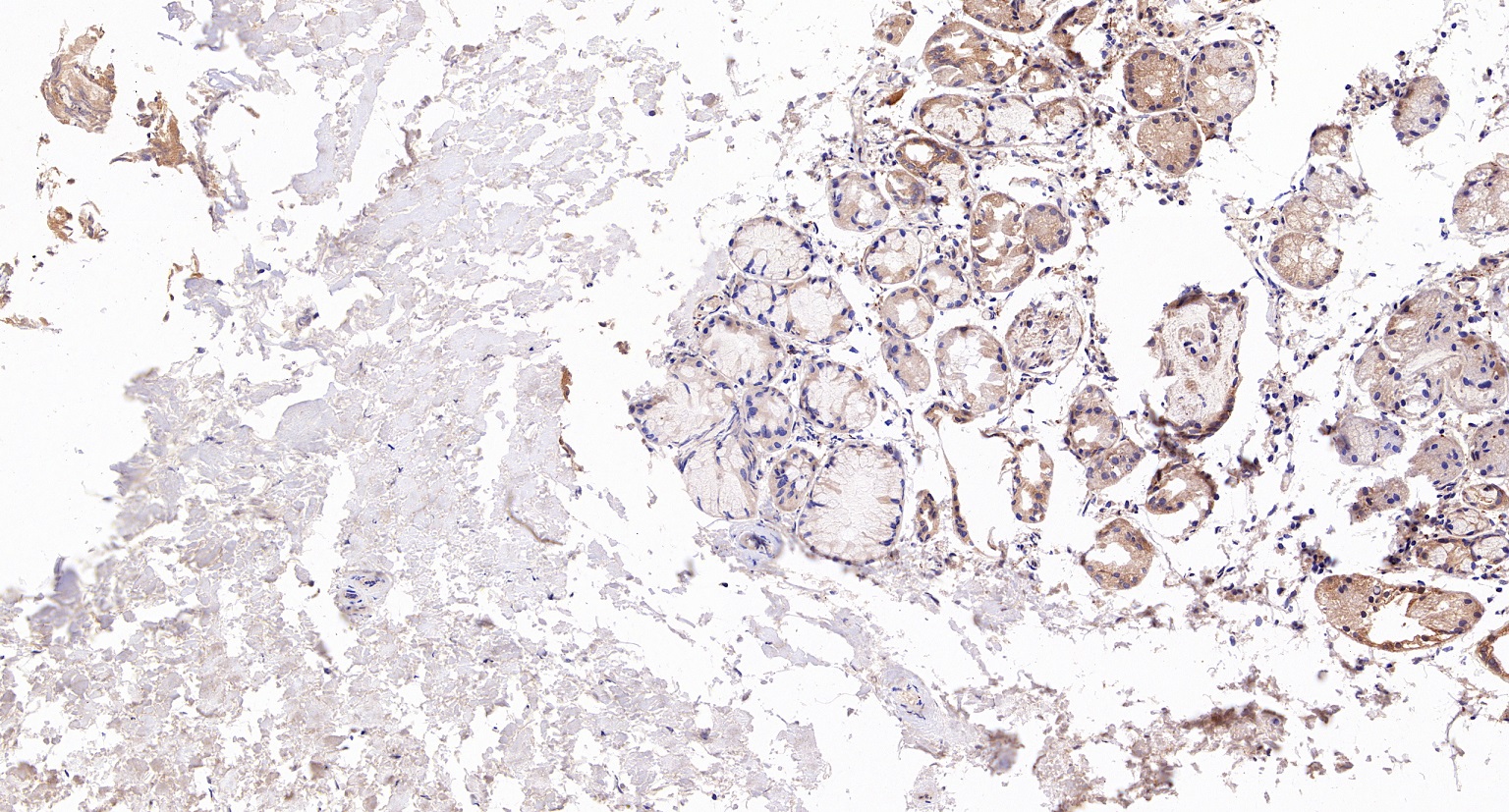

Supplement: Supplemental Information 4 — Immunohistochemical images showing p-ACC immunostaining in the normal tissue adjacent to laryngocarcinoma tissue. [file peerj-07-7037-s004.zip › Raw data-4/p-ACC expression in HNSCC (immunohistochemical analysis)/12-L-N34-pACC.jpg]

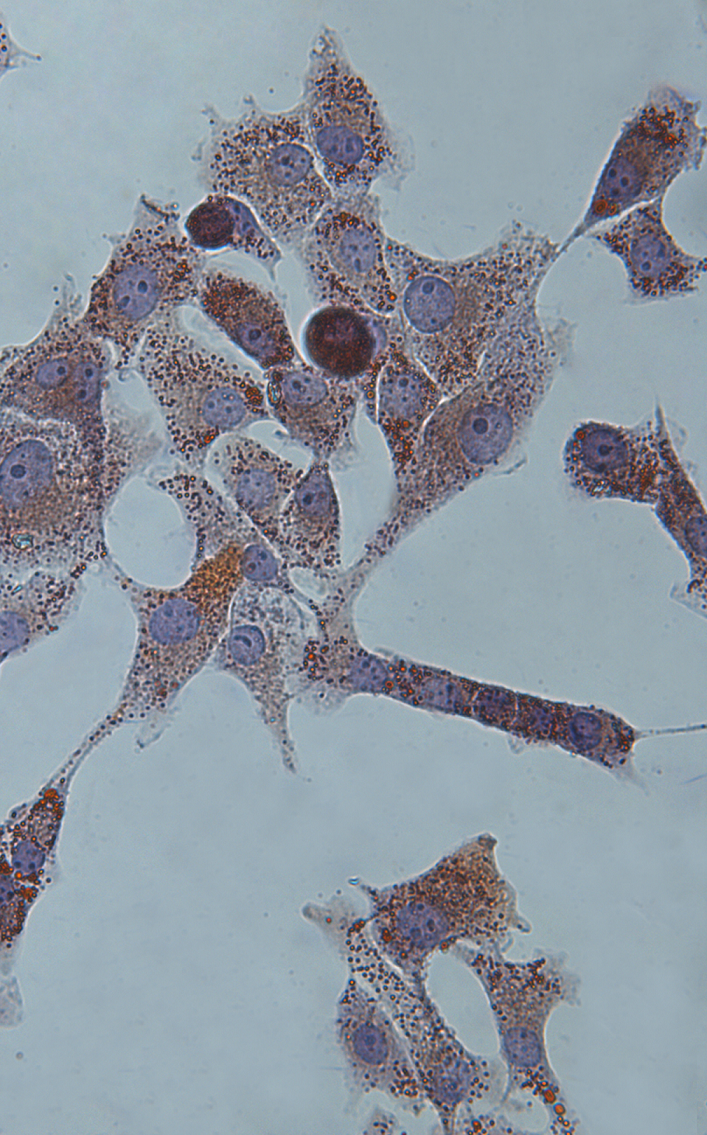

Supplement: Supplemental Information 5 — Images showing the level of lipids in FaDu cells transfected with scrambled siRNA and ACC2 siRNA. [file peerj-07-7037-s005.zip › Raw data-5/Oil red lipid stain results/NC 1.png]

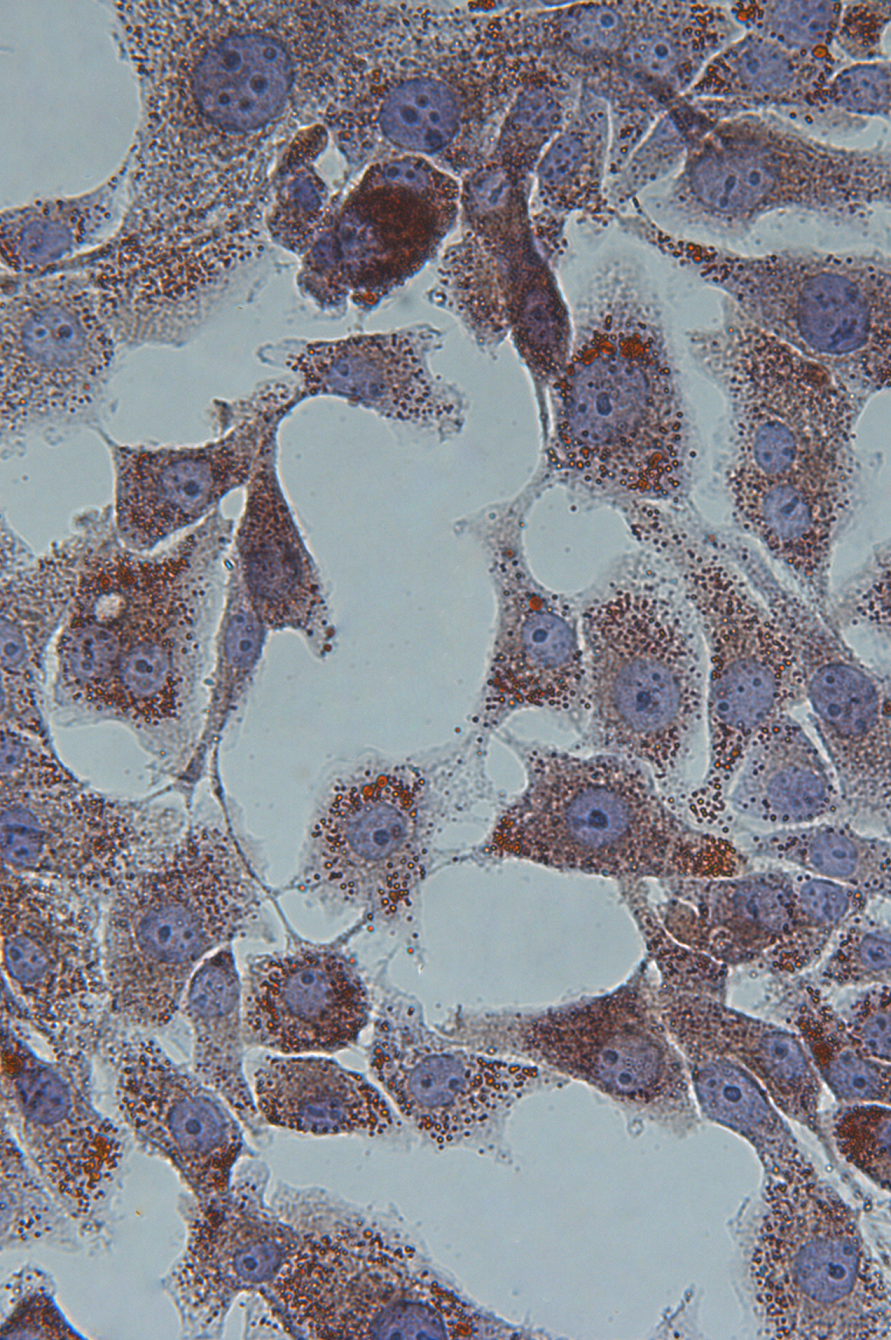

Supplement: Supplemental Information 5 — Images showing the level of lipids in FaDu cells transfected with scrambled siRNA and ACC2 siRNA. [file peerj-07-7037-s005.zip › Raw data-5/Oil red lipid stain results/NC 2.png]

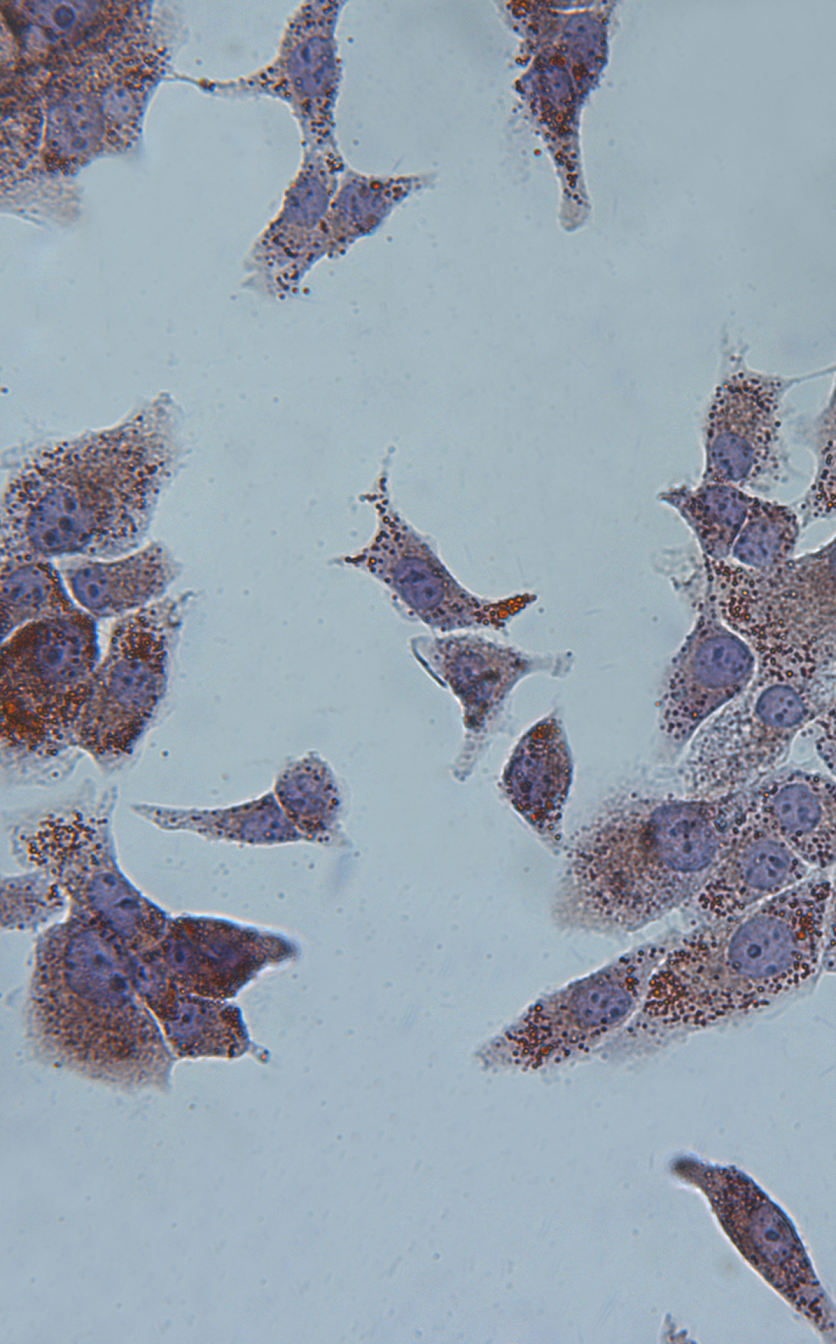

Supplement: Supplemental Information 5 — Images showing the level of lipids in FaDu cells transfected with scrambled siRNA and ACC2 siRNA. [file peerj-07-7037-s005.zip › Raw data-5/Oil red lipid stain results/NC 3.png]

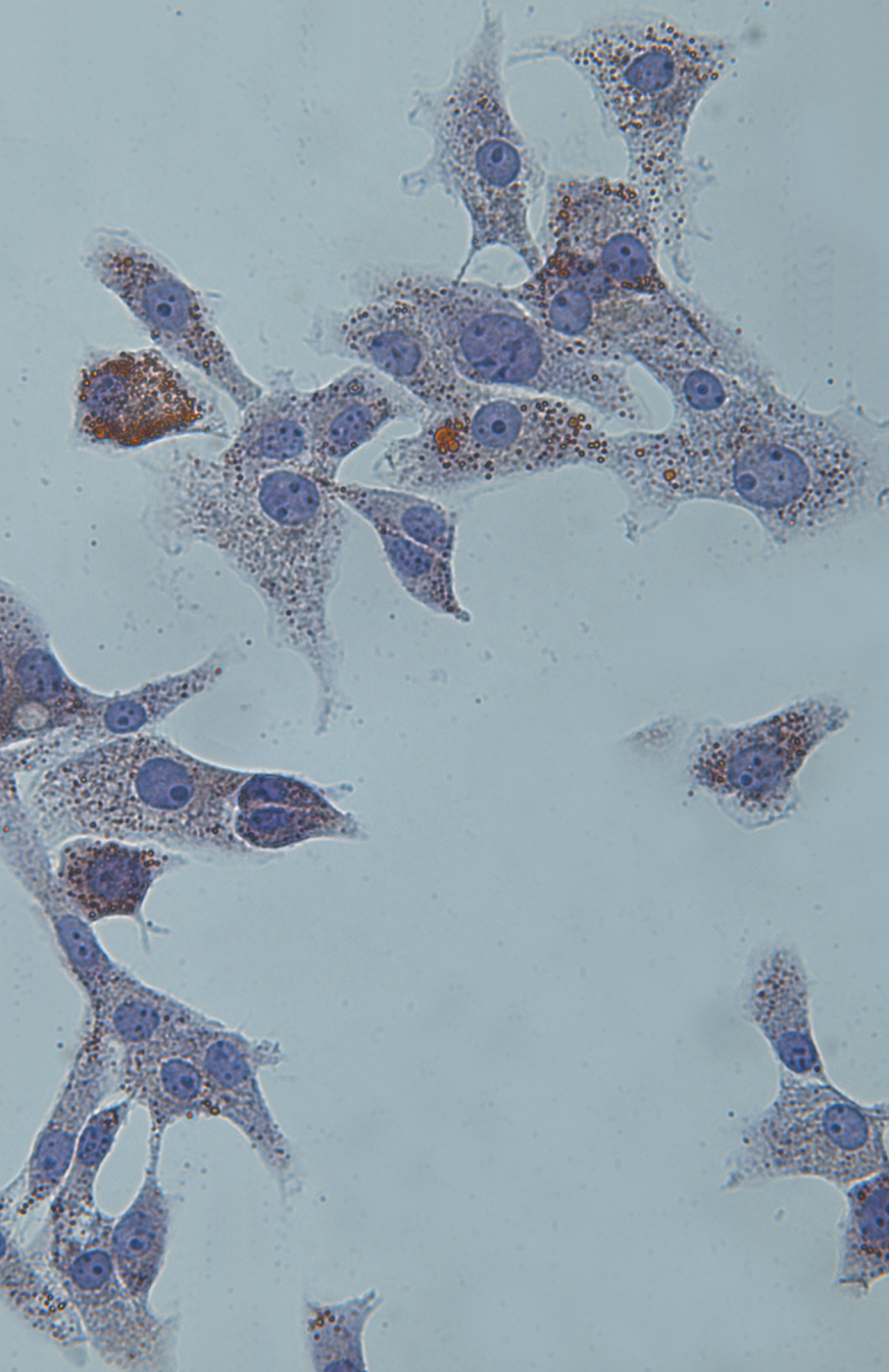

Supplement: Supplemental Information 5 — Images showing the level of lipids in FaDu cells transfected with scrambled siRNA and ACC2 siRNA. [file peerj-07-7037-s005.zip › Raw data-5/Oil red lipid stain results/Transfected with ACC2 siRNA 1.png]

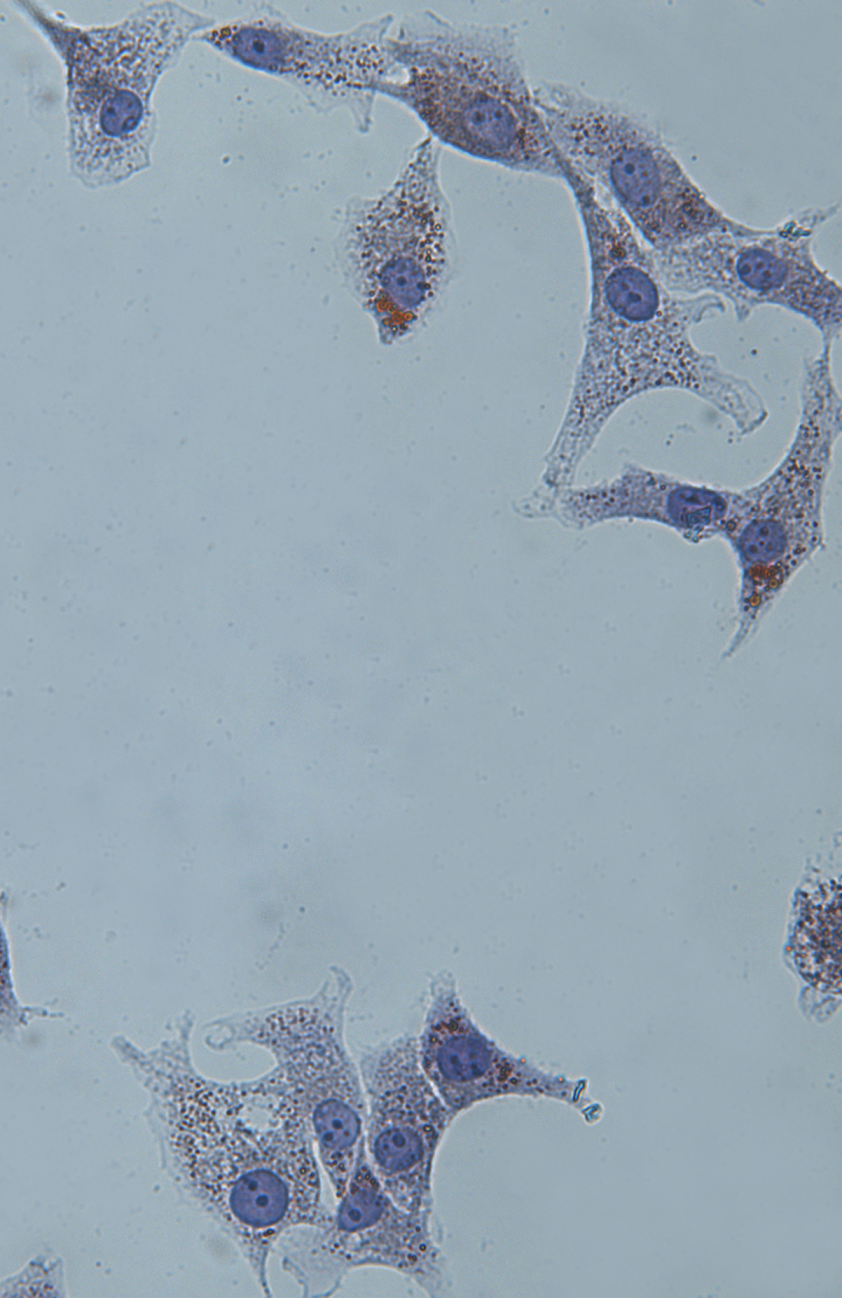

Supplement: Supplemental Information 5 — Images showing the level of lipids in FaDu cells transfected with scrambled siRNA and ACC2 siRNA. [file peerj-07-7037-s005.zip › Raw data-5/Oil red lipid stain results/Transfected with ACC2 siRNA 2.png]

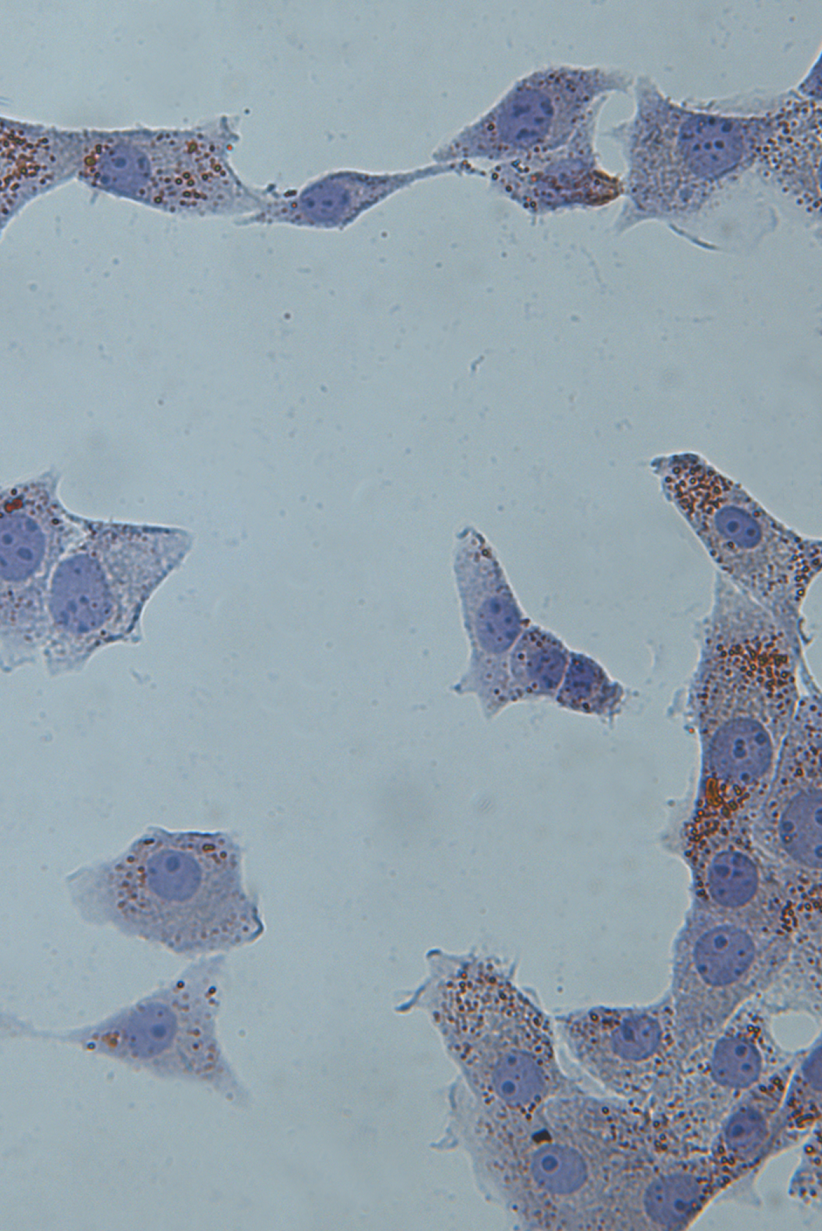

Supplement: Supplemental Information 5 — Images showing the level of lipids in FaDu cells transfected with scrambled siRNA and ACC2 siRNA. [file peerj-07-7037-s005.zip › Raw data-5/Oil red lipid stain results/Transfected with ACC2 siRNA 3.png]

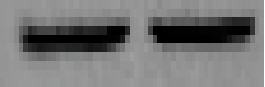

Supplement: Supplemental Information 6 — Western blot images showing p-ACC expression in laryngocarcinoma tissue and the adjacent normal tissue. [file peerj-07-7037-s006.zip › Raw data-6/p-ACC expression in HNSCC (WB analysis)/GAPDH(adjacent tissue-laryngocarcinoma)-1.png]

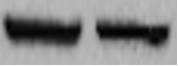

Supplement: Supplemental Information 6 — Western blot images showing p-ACC expression in laryngocarcinoma tissue and the adjacent normal tissue. [file peerj-07-7037-s006.zip › Raw data-6/p-ACC expression in HNSCC (WB analysis)/GAPDH(adjacent tissue-laryngocarcinoma)-2.png]

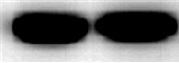

Supplement: Supplemental Information 6 — Western blot images showing p-ACC expression in laryngocarcinoma tissue and the adjacent normal tissue. [file peerj-07-7037-s006.zip › Raw data-6/p-ACC expression in HNSCC (WB analysis)/GAPDH(adjacent tissue-laryngocarcinoma)-3.png]

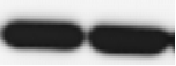

Supplement: Supplemental Information 6 — Western blot images showing p-ACC expression in laryngocarcinoma tissue and the adjacent normal tissue. [file peerj-07-7037-s006.zip › Raw data-6/p-ACC expression in HNSCC (WB analysis)/GAPDH(adjacent tissue-laryngocarcinoma)-4.png]

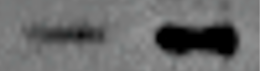

Supplement: Supplemental Information 6 — Western blot images showing p-ACC expression in laryngocarcinoma tissue and the adjacent normal tissue. [file peerj-07-7037-s006.zip › Raw data-6/p-ACC expression in HNSCC (WB analysis)/p-ACC(adjacent tissue-laryngocarcinoma)-1.png]

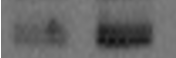

Supplement: Supplemental Information 6 — Western blot images showing p-ACC expression in laryngocarcinoma tissue and the adjacent normal tissue. [file peerj-07-7037-s006.zip › Raw data-6/p-ACC expression in HNSCC (WB analysis)/p-ACC(adjacent tissue-laryngocarcinoma)-2.png]

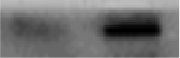

Supplement: Supplemental Information 6 — Western blot images showing p-ACC expression in laryngocarcinoma tissue and the adjacent normal tissue. [file peerj-07-7037-s006.zip › Raw data-6/p-ACC expression in HNSCC (WB analysis)/p-ACC(adjacent tissue-laryngocarcinoma)-3.png]

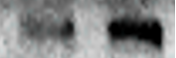

Supplement: Supplemental Information 6 — Western blot images showing p-ACC expression in laryngocarcinoma tissue and the adjacent normal tissue. [file peerj-07-7037-s006.zip › Raw data-6/p-ACC expression in HNSCC (WB analysis)/p-ACC(adjacent tissue-laryngocarcinoma)-4.png]

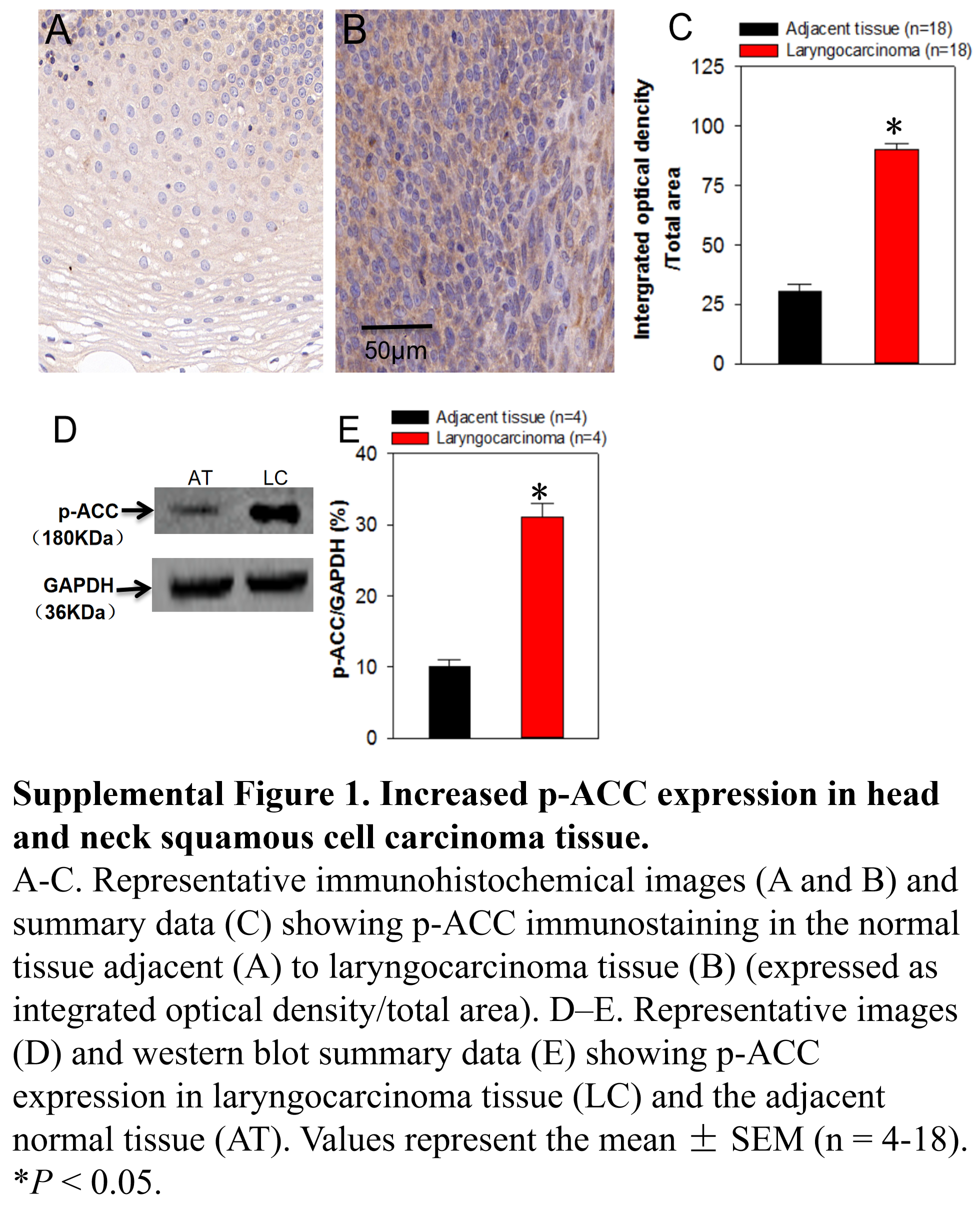

Supplement: Supplemental Information 9 — (A-C) Representative immunohistochemical images (A and B) and summary data (C) showing p-ACC immunostaining in the normal tissue adjacent (A) to laryngocarcinoma tissue (B) (expressed as integrated optical density/total area). D–E. Representative images (D) and western blot summary data (E) showing p-ACC expression in laryngocarcinoma tissue (LC) and the adjacent normal tissue (AT). Values represent the mean ± SEM (n = 4-18). *P < 0.05. [file peerj-07-7037-s009.png]

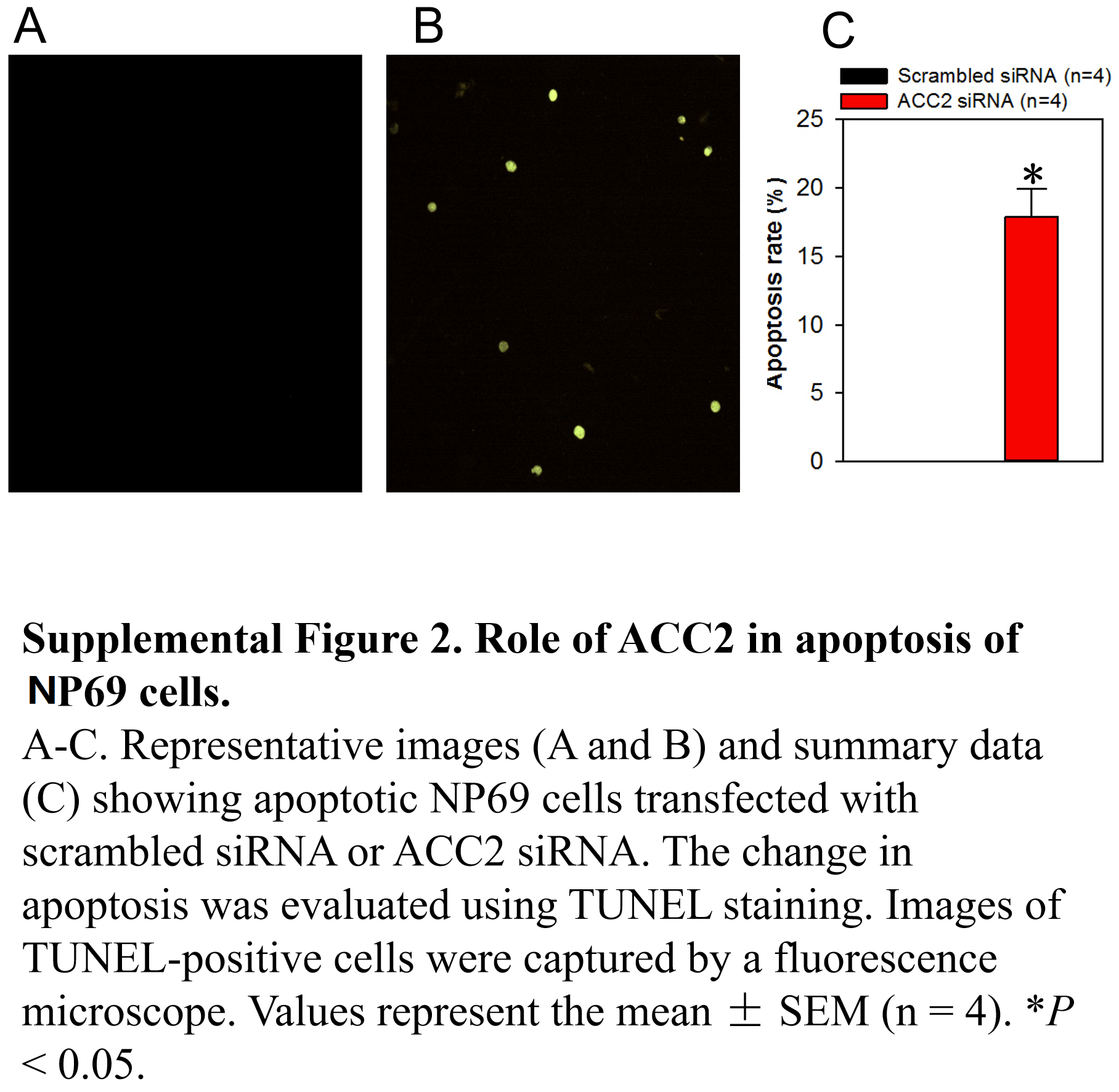

Supplement: Supplemental Information 10 — (A-C) Representative images (A and B) and summary data (C) showing apoptotic NP69 cells transfected with scrambled siRNA or ACC2 siRNA. The change in apoptosis was evaluated using TUNEL staining. Images of TUNEL-positive cells were captured by a fluorescence microscope. Values represent the mean ± SEM (n = 4). *P < 0.05.. [file peerj-07-7037-s010.png]
