# Supplementary material for: The role of acetyl-coA carboxylase2 in head and neck squamous cell carcinoma
Source: PeerJ. 2019 Jun 11;7:e7037. doi: 10.7717/peerj.7037 (PMC6568254; doi:10.7717/peerj.7037)
Supplement: Supplemental Information 7 — Numerical data including immunohistochemistry, westernblot, CCK8, apoptosis rate, and survival data. [file peerj-07-7037-s007.zip › Numerical data/Survival analysis.doc]

KM 月 BY 分析值分级
  /STATUS=生存状态(1)
  /PRINT TABLE MEAN
  /PLOT SURVIVAL
  /TEST LOGRANK
  /COMPARE OVERALL POOLED
  /SAVE SURVIVAL.


Kaplan-Meier


附注	
创建的输出	24-APR-2018 21:24:42	
注释		
输入	活动的数据集	数据集1	
	过滤器	<none>	
	权重	<none>	
	拆分文件	<none>	
	工作数据文件中的 N 行	51	
缺失值处理	缺失的定义	用户自定义缺失值被视为缺失。	
	使用的个案	对于分析中的所有变量而言，统计量以带有有效数据的所有个案为基础。	
语法	KM 月 BY 分析值分级
  /STATUS=生存状态(1)
  /PRINT TABLE MEAN
  /PLOT SURVIVAL
  /TEST LOGRANK
  /COMPARE OVERALL POOLED
  /SAVE SURVIVAL.	
资源	处理器时间	00:00:00.62	
	已用时间	00:00:00.61	
已创建或修改的变量	SUR_1	生存函数	


[数据集1] 


个案处理摘要	
分析值分级	总数	事件数	删失	
			N	百分比	
high ACC	19	15	4	21.1%	
low ACC	32	5	27	84.4%	
整体	51	20	31	60.8%	


生存表	
分析值分级	时间	状态	此时生存的累积比例	累积事件数	剩余个案数	
			估计	标准误			
high ACC	1	5.000	1	.	.	1	18	
	2	5.000	1	.895	.070	2	17	
	3	8.000	1	.842	.084	3	16	
	4	11.000	1	.789	.094	4	15	
	5	16.000	1	.737	.101	5	14	
	6	17.000	1	.	.	6	13	
	7	17.000	1	.632	.111	7	12	
	8	20.000	1	.579	.113	8	11	
	9	23.000	1	.526	.115	9	10	
	10	26.000	1	.474	.115	10	9	
	11	28.000	1	.	.	11	8	
	12	28.000	1	.368	.111	12	7	
	13	34.000	1	.316	.107	13	6	
	14	55.000	1	.263	.101	14	5	
	15	59.000	1	.211	.094	15	4	
	16	61.000	0	.	.	15	3	
	17	61.000	0	.	.	15	2	
	18	61.000	0	.	.	15	1	
	19	61.000	0	.	.	15	0	
low ACC	1	16.000	1	.969	.031	1	31	
	2	27.000	1	.938	.043	2	30	
	3	38.000	1	.906	.052	3	29	
	4	40.000	1	.875	.058	4	28	
	5	60.000	1	.844	.064	5	27	
	6	61.000	0	.	.	5	26	
	7	61.000	0	.	.	5	25	
	8	61.000	0	.	.	5	24	
	9	61.000	0	.	.	5	23	
	10	61.000	0	.	.	5	22	
	11	61.000	0	.	.	5	21	
	12	61.000	0	.	.	5	20	
	13	61.000	0	.	.	5	19	
	14	61.000	0	.	.	5	18	
	15	61.000	0	.	.	5	17	
	16	61.000	0	.	.	5	16	

生存表	
分析值分级	时间	状态	此时生存的累积比例	累积事件数	剩余个案数	
			估计	标准误			
low ACC	17	61.000	0	.	.	5	15	
	18	61.000	0	.	.	5	14	
	19	61.000	0	.	.	5	13	
	20	61.000	0	.	.	5	12	
	21	61.000	0	.	.	5	11	
	22	61.000	0	.	.	5	10	
	23	61.000	0	.	.	5	9	
	24	61.000	0	.	.	5	8	
	25	61.000	0	.	.	5	7	
	26	61.000	0	.	.	5	6	
	27	61.000	0	.	.	5	5	
	28	61.000	0	.	.	5	4	
	29	61.000	0	.	.	5	3	
	30	61.000	0	.	.	5	2	
	31	61.000	0	.	.	5	1	
	32	61.000	0	.	.	5	0	


生存表的均值和中位数	
分析值分级	均值a	中位数	
	估计	标准误	95% 置信区间	估计	标准误	95% 置信区间	
			下限	上限			下限	
high ACC	31.368	4.735	22.089	40.648	26.000	4.353	17.468	
low ACC	57.125	1.894	53.414	60.836	.	.	.	
整体	47.529	2.750	42.139	52.920	.	.	.	

生存表的均值和中位数	
分析值分级	中位数a	
	95% 置信区间	
	上限	
high ACC	34.532	
low ACC	.	
整体	.	

a. 如果估计值已删失，那么它将限制为最长的生存时间。	


整体比较	
	卡方	df	Sig.	
Log Rank (Mantel-Cox)	26.521	1	.000	

为 分析值分级 的不同水平检验生存分布等同性。	
